# Real-time exploration of the billion-scale human repertoire for precursor frequency analysis and rational antibody design

**DOI:** 10.3389/fimmu.2026.1958625

**Published:** 2026-10-16

**Authors:** Tom U. Schlegel, Jannis von Blanckenburg de Riz, Jakob R. Riccabona, Franz Dietzmeyer, Julia Koehler Leman, Jens Meiler, Clara T. Schoeder, Torben Schiffner

**Affiliations:** 1 Institute for Drug Discovery, Faculty of Medicine, Leipzig University, Leipzig, Germany; 2 Center for Scalable Data Analytics and Artificial Intelligence ScaDS.AI, Dresden/Leipzig, Germany; 3 Open Molecular Software Foundation, Berkeley, CA, United States; 4 School of Embedded Composite Artificial Intelligence SECAI, Dresden/Leipzig, Germany; 5 Institute for Computer Science, Wilhelm Ostwald Institute for Physical and Theoretical Chemistry, University Leipzig, Leipzig, Germany; 6 Department of Chemistry, Department of Pharmacology, Center for Structural Biology, Institute of Chemical Biology, Center for Applied Artificial Intelligence in Protein Dynamics, Vanderbilt University, Nashville, TN, United States; 7 Department of Immunology and Microbiology, The Scripps Research Institute, La Jolla, CA, United States; 8 Vaccine and Immunotherapy Center, The Wistar Institute, Philadelphia, PA, United States

**Keywords:** antibody repertoire analysis, broadly neutralizing antibodies, germline targeting, observed antibody space, rational antibody design, webtool

## Abstract

The success of germline-targeting vaccines and rational antibody design depends on quantifying the population-level frequencies of specific antibody features, such as CDR lengths, sequence motifs, germline usage or preferred chain pairings, across the natural human repertoire. The maturation of high-throughput sequencing has generated large database repertoires containing billions of sequences, allowing researchers to assess naïve B-cell precursor frequencies and evaluate biologically plausible chain combinations. However, leveraging this scale of data remains a significant bottleneck, as current databases trap scientists between computationally demanding bulk downloads, restrictive, single-purpose web interfaces or outdated datasets. To address this gap, we introduce ABHunter, an automatically updated, open-access framework designed to explore and filter the healthy-human subset of the Observed Antibody Space (OAS) database, which currently stands at ~1.86-billion-sequence as of January 2026. Accessible via any web browser, the platform executes searches with median times ranging from 3.7 seconds for simple gene usage profiles to 20.8 seconds for complex paratope motifs, with full sequence retrieval completed in 118 seconds for 1 million hits. The interface allows users to quantify antibody feature frequencies to assess the natural abundance of targeted B-cells across various isotypes (e.g., utilizing IgM for precursor B-cell analysis), as well as map native repertoire contexts to scaffold isolated features. Additionally, it predicts biologically plausible inter-chain pairings for therapeutic design by using a statistical co-occurrence model that extrapolates pairing probabilities from the limited natively paired subset comprising 0.03% of the data across six donors. Furthermore, every search generates a comprehensive statistical landscape, detailing underlying complementarity-determining regions length distributions, amino acid compositions, and gene coupling patterns. For custom analysis and integration into bioinformatic pipelines, the optimized data structure reduces the storage footprint to 106 GB, allowing the entire tool to run locally on a standard laptop. ABHunter eliminates the compromise between accessibility and flexibility, transforming large-scale sequencing data into a universally accessible resource for next-generation therapeutic discovery. The platform is freely available at abhunter.org.

## Graphical Abstract

**Figure:**
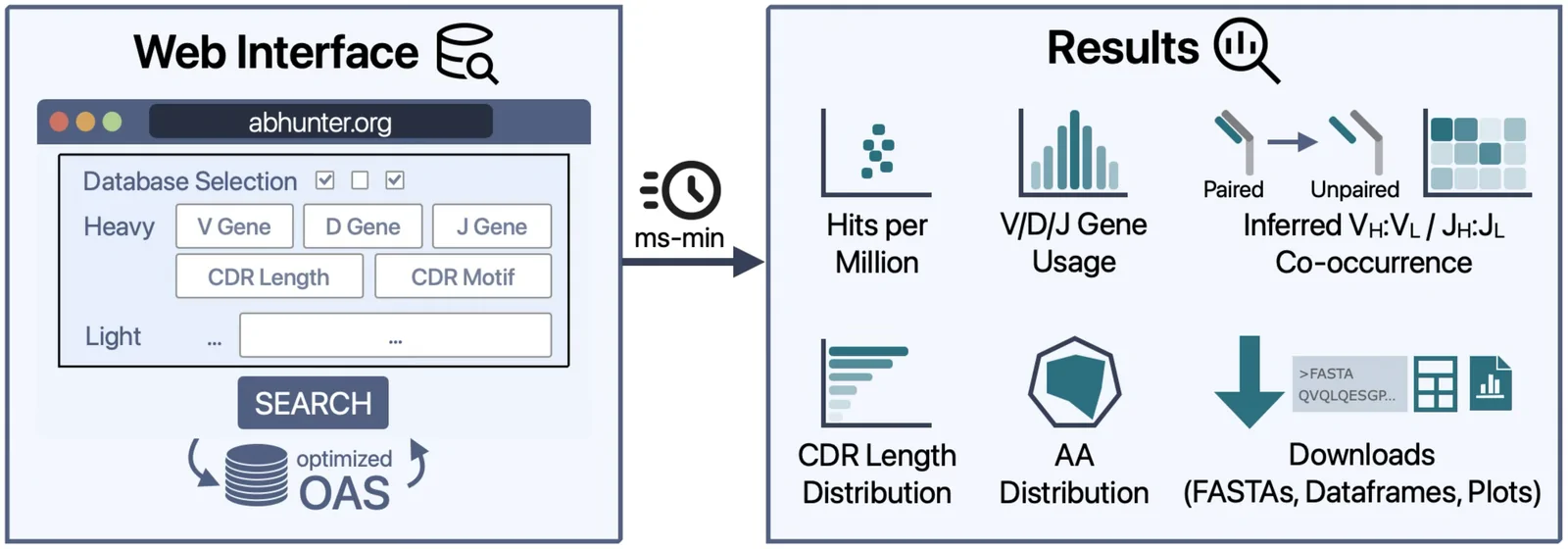

## 1 Introduction

The adaptive immune system relies on a diverse repertoire of B-cell receptors and their secreted antibody counterparts to recognize, bind to, and neutralize a vast and changing array of pathogenic antigens (1–4). While an individual human body contains approximately 10^11^ B cells at any given time, the theoretical sequence space is much greater (5–9). Driven by variable (V), diversity (D) and joining (J) gene segment recombination during development, imprecise junctional joining and antigen-driven somatic hypermutation within the six highly variable complementarity-determining regions (CDRs), combinatorial calculations predict a theoretical repertoire of 10^13^ to 10^18^ distinct sequences (4–8, 10–16). However, experimentally capturing this large genetic diversity and the native physiological pairing of heavy and light chains at high throughput has remained a major technical challenge.

Over the past decade, the maturation of high-throughput next-generation sequencing methods, ranging from unpaired bulk approaches (17–20) to heavy-light chain-preserving techniques (6, 9, 21–35), has overcome this limitation, enabling profiling of human antibody repertoires at large scale across diverse individuals and populations (36, 37). While these methods provide detailed insights into the mechanics of V(D)J recombination and the baseline architecture of human immunity, they have also introduced a new challenge by generating vast, highly complex datasets. To manage this massive output, the Adaptive Immune Receptor Repertoire (AIRR) Community of The Antibody Society (38) developed critical standardization initiatives for storing, curating and sharing immune repertoire sequence data (39–42). These guidelines established the foundation for centralized immunological databases that aggregate data across independent studies and have since become cornerstones of the field (43–46).

A critical prerequisite for advanced therapeutic discovery is the ability to identify the frequencies and distributions of antibodies with defined properties, such as shared germline origins, specific CDR lengths and motifs, or preferred heavy-light chain pairings (20, 47, 48). The emerging technique of germline-targeting vaccine design relies on engineered immunogens to specifically activate B-cell precursors with pre-defined genetic and structural features and subsequently guide their maturation into broadly neutralizing antibodies (bnAbs). It has previously been shown that activation and the competitive fitness of B cells in germinal centers rely on both their affinity for the immunogen and their frequency, with high-frequency B cells being able to dominate the germinal center even at lower affinity. This finding makes determining the frequency of precursor lineages in the population critical for germline-targeting vaccine design (49–63). Evaluating these distributions is also important for rational antibody design where, identifying complementary chain partners enables the rescue of isolated single-chain leads and facilitates the rational scaffolding of isolated binding motifs by grafting them into natively validated frameworks. Rather than forcing arbitrary or functionally incompatible combinations of features, relying on these naturally occurring structural partners allows researchers to design viable biotherapeutics with a reduced risk of biophysical instability or poor expression (19, 63–69). Simultaneously, mapping the underlying structural and genetic constraints provides insights into the deterministic rules governing natural antibody assembly within the human immune system (1, 4, 20, 21, 36, 37, 48, 70–77). Ultimately, whether answering these targeted biological questions or analyzing broader architectural patterns of human immunity, researchers need to be able to perform statistical analyses across the entire available global repertoire.

Extracting biological insights from available large datasets remains a severe bottleneck, as existing platforms force researchers to compromise between analytical flexibility and ease of use. Many established repositories restrict users to bulk data downloads, yielding thousands of curated yet raw sequence files (45, 46, 70, 71, 78). Although serving as a crucial foundation for machine learning applications (79–81), locally processing and analyzing these files requires specialized bioinformatic expertise, large storage architectures, and hours or days of compute time, making a full local database installation practically impossible for bench scientists on consumer hardware. While recent tools overcome this speed bottleneck by enabling rapid sequence mining across billions of sequences on personal computers (82, 83), they lack graphical user interfaces and have limited search capabilities, typically restricted to sequence similarity or isolated CDR3 matching. On the other hand, platforms that offer web interfaces often rely on rigid, single-purpose workflows hosted on remote servers mostly with no option for local deployment (43, 44, 84–89). Some of these tools are hindered by unintuitive interfaces, insufficient documentation, a lack of active support, and outdated data that fails to keep pace with the steadily growing volume of newly published repertoire studies (**Supplementary Table 1**). Thus, a critical unmet need remains for a computationally optimized framework that allows researchers to execute custom, multi-parametric queries in real-time, operating on an automatically updated billion-scale dataset that can easily be hosted both as an intuitive web interface and as a local database designed to fit entirely on standard personal computers.

We hypothesize that by utilizing a column-oriented, storage-optimized database architecture and pre-computing key immunological metrics, we can enable real-time, multi-parametric repertoire searches and statistical profiling on standard personal computers, while keeping the database automatically updated and easily accessible via either an intuitive web browser or a local installation where the entire dataset fits locally. To address this, our objective was to develop and validate ABHunter, a scalable framework that makes the ~1.86-billion-sequence healthy-human subset of the Observed Antibody Space (OAS) universally accessible. By combining deep analytical freedom with low-latency execution, ABHunter empowers researchers to perform critical biological analyses that could not easily be executed before without writing custom, computationally demanding pipelines: 1. What are the population-level precursor frequencies of therapeutic or vaccine-elicited antibodies under defined structural and genetic constraints? 2. How do specific, defined antibody features constrain other properties in the native repertoire context? 3. How can the vast antibody repertoire landscape of the dataset be explored on custom terms without being restricted to fixed query rules?

In this work, we developed ABHunter, a framework designed for the exploration and filtering of the ~1.86-billion-sequence healthy-human subset of the Observed Antibody Space (OAS) in a matter of seconds. Our tool is accessible through any standard web browser (abhunter.org) and supports deeper, custom analysis through local installation that was previously impractical, making the datasets curated by Olsen et al. (45) and Kovaltsuk et al. (46) broadly accessible. After demonstrating the performance and storage advantages of ABHunter, we analyze heavy-light chain coupling within the paired repertoire to establish a statistically inferred pairing model. Finally, we demonstrate the framework’s versatility through specific use cases, including quantifying precursor frequencies for germline-targeting vaccine strategies, guiding rational antibody design, and conducting high-dimensional statistical analysis of the global healthy-human OAS.

## 2 Results

### 2.1 ABHunter: A scalable framework for discovery and analysis of the Observed Antibody Space (OAS) in humans

#### 2.1.1 The ABHunter web application enables high-level exploration and filtering of the human antibody repertoire via standard web browsers

To allow users to explore and filter the ~1.86 billion-sequence antibody repertoire of healthy humans from the OAS database without any programming expertise, we developed the ABHunter web application (**Figure 1A**). Deployed as a cloud-hosted web application (http://abhunter.org/), the interactive interface provides a multi-parametric search mask (**Figure 2A**), supports complex antibody profiling across unpaired and paired datasets using V(D)J germline filtering (family, subfamily, or allele) and allows users to set precise CDR1–3 length constraints following IMGT definitions. To isolate specific chemical signatures or binding motifs, the system implements a simplified regular expression (regex) search syntax for amino acid sequences, including wildcards, placeholders, and residue ranges. For locating rare precursors with specific epitope-binding signatures, the interface further supports the search for similar amino acids, grouped by physicochemical properties, within a CDR motif. Users can also define a substitution threshold to identify sequence variants with conserved properties. For sequence-based input, an integrated NCBI IgBLAST module automates the annotation of raw nucleotide data and populates the search mask with the corresponding gene segments and motifs.

**Figure 1:**
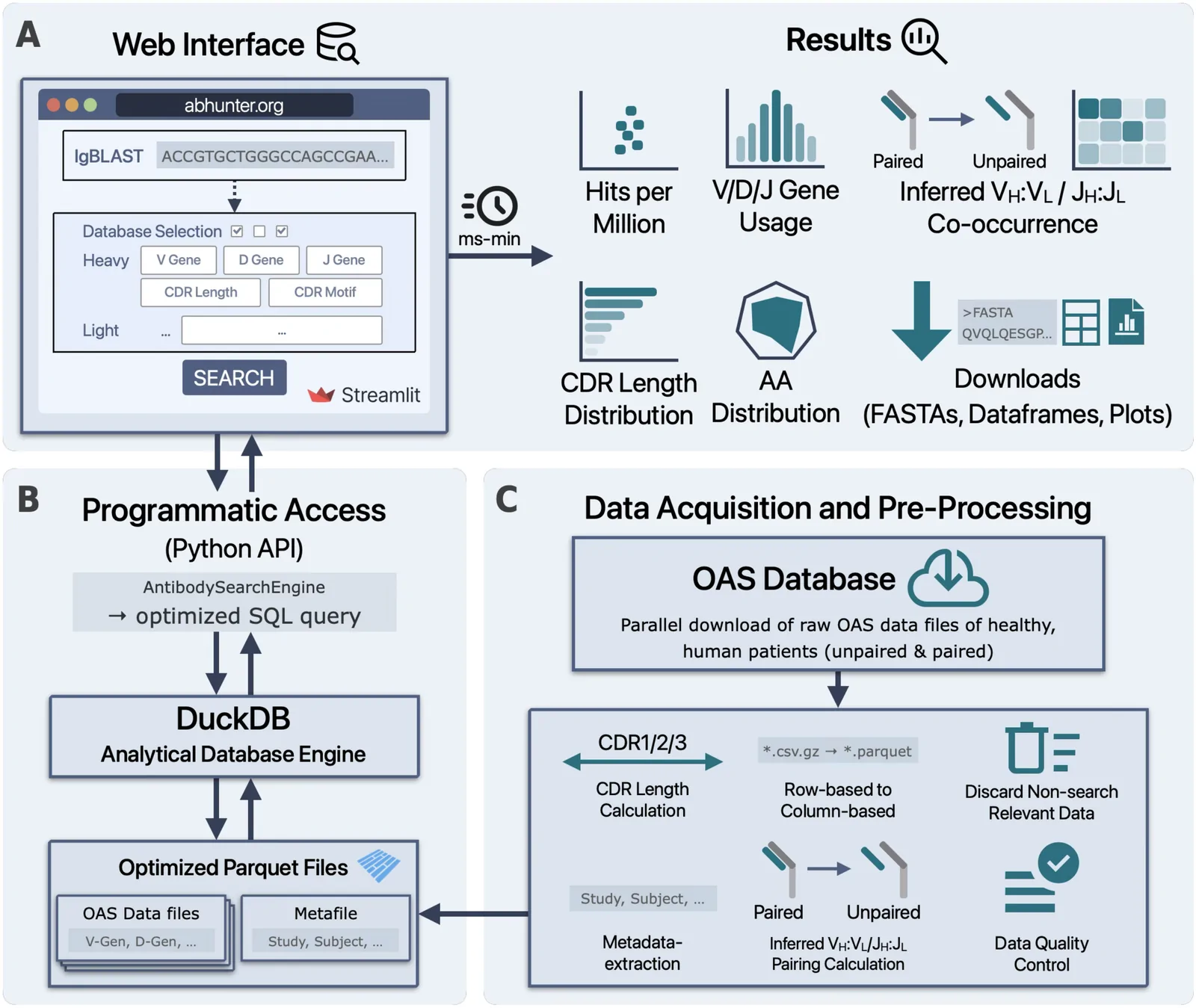
Web user interface and backend of the ABHunter framework. ABHunter is a framework for analyzing the healthy-human Observed Antibody Space (OAS) at the population level. **(A)** Streamlit Web Interface: The web application provides a multi-parametric search mask interface that allows users to define target populations by filtering for specific germline genes, CDR length ranges, and amino acid motifs. For sequence-based queries, an automated annotation of raw nucleotide sequences and their conversion into search parameters is facilitated by an integrated NCBI IgBLAST module. Once configured, the interface provides interactive visualizations of the search results, including precursor frequency plots, gene usage distributions, estimated IGHV : IG(K/L)V and IGHJ : IG(K/L)J pairing matrices and more. To facilitate downstream analysis, the framework allows the direct export of both processed datasets and generated plots in standardized format for further bioinformatic evaluation. **(B)** Search engine: To scan billions of sequences in seconds without memory overhead, the search inputs are first translated into SQL queries by the AntibodySearchEngine, which are then executed as vectorized in-memory SQL queries on the optimized parquet data files [see (**C**)] using DuckDB. Conversely, the AntibodySearchEngine supplies search results in a format compatible with the web interface. **(C)** Data acquisition and pre-processing: To reduce storage requirements and enable efficient data access, the raw data files from healthy humans from the OAS were streamlined into optimized Apache Parquet files. This pre-processing step included focusing the corpus on only essential immunological features, metadata-extraction, and the pre-calculation of certain metrics, such as the CDR length or the statistically inferred pairing model.

**Figure 2:**
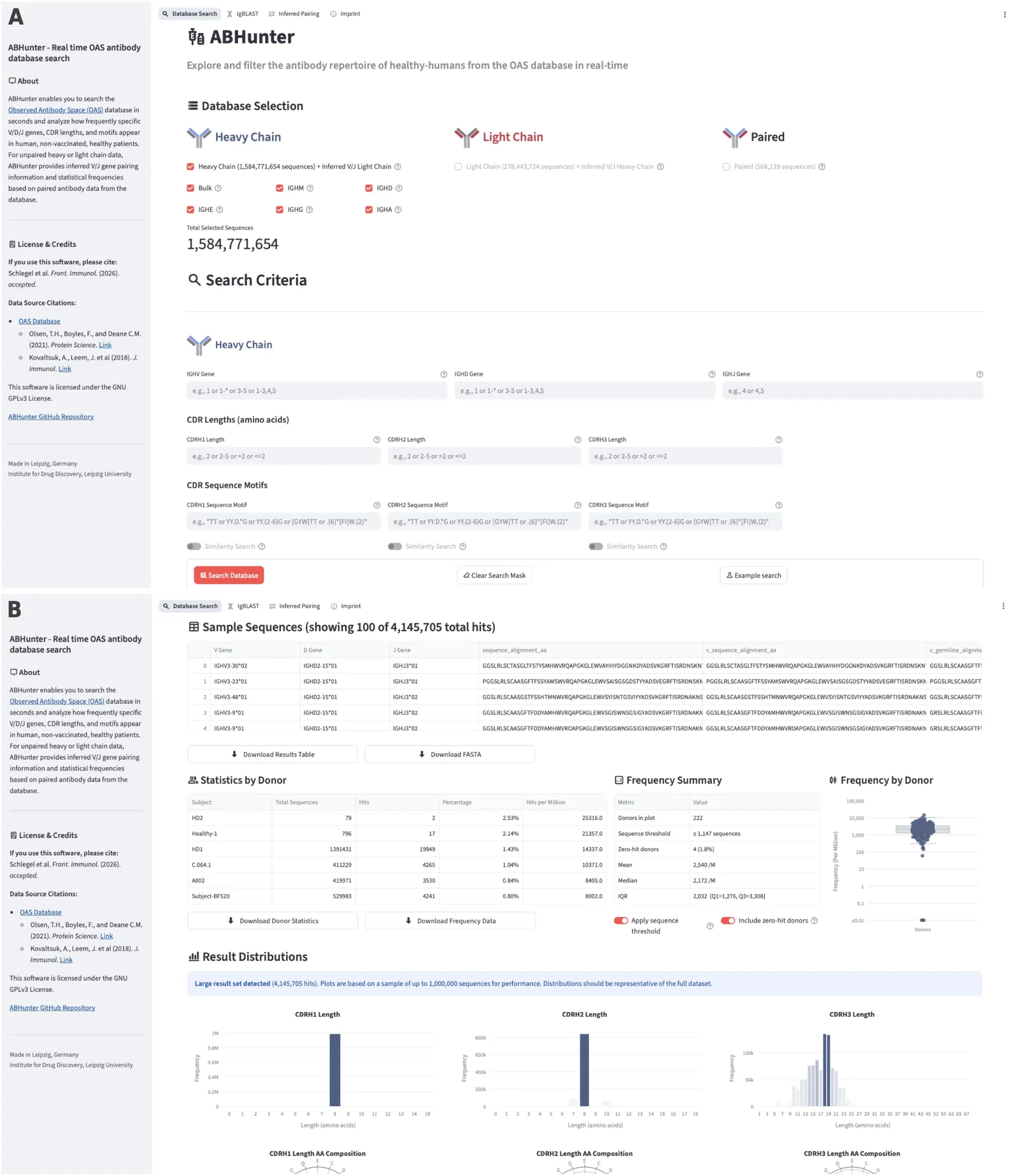
ABHunter web interface. **(A)** Multi-parametric search mask. The graphical user interface of ABHunter allows navigation through the datasets without requiring prior programming knowledge or extensive computational expertise. Database & isotype selection: Users can toggle between the datasets “Unpaired Heavy Chain,” “Unpaired Light Chain,” or “Paired” and further refine their search by selecting specific antibody isotypes. Search Criteria: The interface provides a multi-parametric search mask to filter for specific V(D)J gene segments for heavy and/or light chains via free text input, define CDR1/2/3 length ranges, and input complex amino acid motifs using simplified regular expression (regex) syntax. For sequence-based queries, an automated annotation of raw nucleotide sequences and their conversion into search parameters is facilitated by an integrated NCBI IgBLAST module, which is accessible via the navigation bar at the top. **(B)** Interactive results dashboard. Upon search execution, the interface generates interactive visualizations to convert the identified sequence population into interpretable immunological data. These include normalized hits-per-million plots for standardized prevalence comparisons across individual donor cohorts, histograms for V(D)/J gene usage and CDR length, and radar charts of amino acid compositions.

To ensure search integrity, the interface provides real-time query validation, flagging non-existent genes or invalid parameters before execution. Upon query execution, the framework generates interactive visualizations that convert the raw sequence hits into interpretable immunological data. These include normalized frequency plots for standardized prevalence comparisons, histograms for V(D)J gene usage and CDR length, and radar plots of amino acid compositions (**Figure 2B**). Based on observed biological biases from the paired dataset, a statistically inferred pairing model visualizes the most likely IGHV: IG(K/L)V ​ and IGHJ: IG(K/L)J gene family associations for given V and/or J genes. To facilitate downstream analysis and further independent bioinformatic evaluation, all analytical outputs, including full sequence tables and all generated plots, are exportable in a standardized format (see methods). To ensure seamless deployment and full environmental reproducibility across cloud environments, local servers and personal computers, we provide a Docker container of the full ABHunter framework (https://github.com/SchiffnerLab/abhunter).

#### 2.1.2 A unified search engine architecture facilitates both web-based and advanced local command line access for custom analysis

To standardize data access and encapsulate the underlying database complexity, we developed an AntibodySearchEngine, which translates user-defined search parameters into optimized queries that are fed into the analytical database DuckDB engine (90). This ensures a unified entry point for the framework and allows for search requests through the full dataset without memory overhead. Once the query is completed, the AntibodySearchEngine formats the retrieved data for subsequent analysis tasks and visualization within the ABHunter web interface. For researchers requiring deeper customization, a local installation provides direct command-line access to this engine. This allows raw SQL queries, enabling custom repertoire investigations that go beyond the standardized search filters (**Figure 1B**). Representative command-line examples for AntibodySearchEngine calls and equivalent DuckDB SQL queries are provided in **Supplementary Tables 2** and **3**, respectively.

#### 2.1.3 Optimized data infrastructure and pre-computed metrics facilitate billion-sequence repertoire analysis

To minimize storage and ensure performance, the underlying corpus of ~1.86 billion data points, comprising both natively paired and unpaired sequences, was organized into an optimized data architecture. To maximize computational efficiency, the sequences were converted into a compressed columnar-based parquet format and restricted to essential immunological features, such as amino acid sequences, V(D)J gene calls, CDR sequences and associated study metadata. This format allows for reduced I/O latency, allowing the framework to retrieve specific subsets without the overhead of loading full record-datasets. To further optimize filtering and analysis, sequence-derived features (CDR lengths) and the statistically inferred pairing model parameters were pre-computed during preprocessing (**Figure 1C**).

### 2.2 Evaluating speed and resource efficiency of the ABHunter framework

#### 2.2.1 Minimal storage requirements enable whole-repertoire analysis on standard personal computers

Storage requirements were compared across the entire corpus for raw CSV, Gzip-compressed OAS CSVs, a full-column ZSTD-Parquet representation, and the optimized feature-filtered ZSTD-Parquet format (**Figure 3**; **Supplementary Table 4**). Conversion of the full OAS dataset (all original columns and metadata retained) to a column-based Parquet format reduced the storage footprint from 8476 GB for raw CSVs to 768 GB. By subsequently retaining only essential immunological features (see methods), we achieved a 98.8% reduction in storage footprint compared to the raw CSVs, corresponding to a final size of 106 GB. Against the existing 862 GB OAS Gzip distribution, the 106 GB ABHunter database represents an 87.7% efficiency gain while maintaining the full ~1.86-billion-sequence corpus.

**Figure 3:**
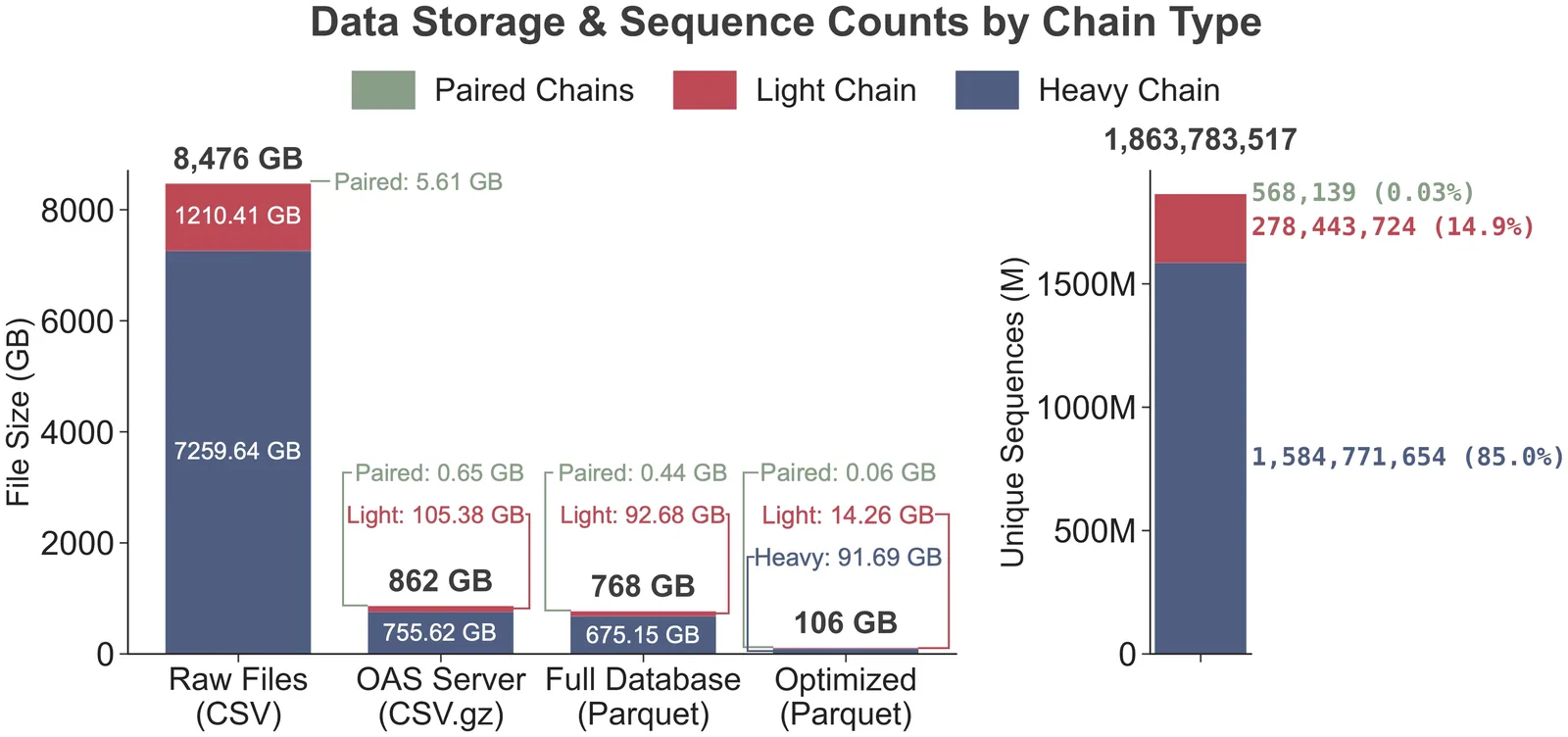
Optimized file formatting and strategic data curation enable the storage of the ~1.86-billion-sequence corpus on consumer-grade hardware. (Left) Storage footprints across four representations. A storage efficiency gain of 87.7% over the original compressed distribution and 98.8% over raw CSVs was achieved through conversion to the optimized DuckDB-compatible ZSTD-Parquet format. Conversion of the full dataset (all original columns and metadata retained) first yielded a 768 GB database, which was reduced to a final size of 106 GB by retaining only essential immunological features. (Right) Unique sequence counts by chain type. The stacked bar plot quantifies the total number of unique sequences by chain type, highlighting that the dataset consists mainly of unpaired heavy-chain sequences, while paired sequences account for only 0.03%.

#### 2.2.2 Search performance overcomes the scaling limitations of traditional file-based workflows

We benchmarked execution time to compare ABHunter’s AntibodySearchEngine against a non-database, in-memory Pandas baseline (**Figure 4A**; **Supplementary Table 5**). Across subsets ranging from ~38,000 to ~217,000 sequences (0.002-0.014% of the full unpaired heavy corpus), ABHunter’s latency increased from 0.031 ± 0.001 seconds to 0.072 ± 0.005 seconds. In contrast, traditional CSV-based workflows scaled much more steeply. Uncompressed CSV latency rose from 1.5 ± 0.03 seconds to 7.9 ± 0.28 seconds, while Gzip compressed files reached 9.2 ± 0.20 seconds at the largest subset, reflecting decompression overhead. This performance gap widened progressively with data volume. At the maximum tested subset size, ABHunter achieved a 110x speedup over raw CSV and a 127x speedup over Gzip.

**Figure 4:**
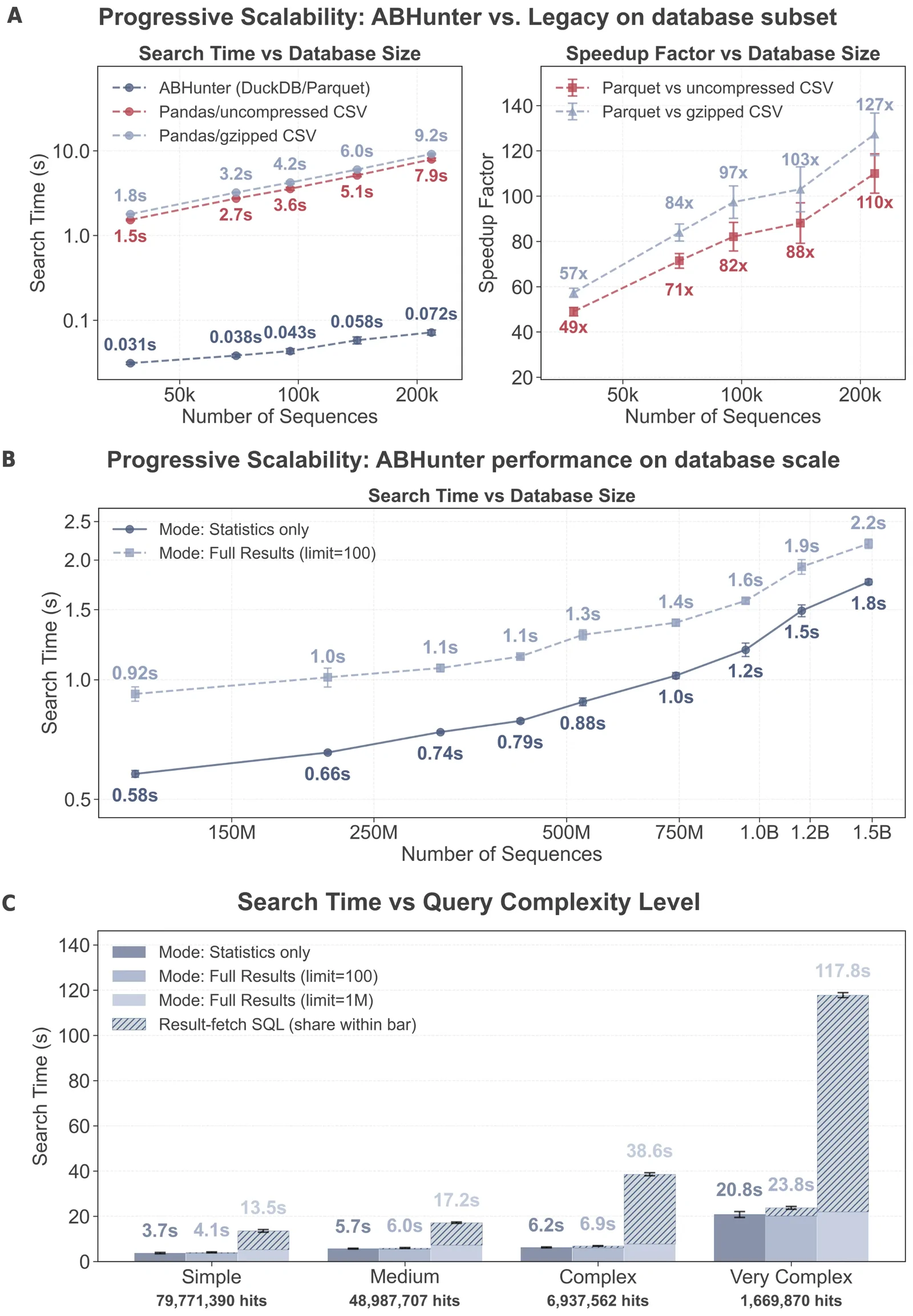
Computational Performance and Scalability of ABHunter. **(A)** Performance advantage over traditional row-based legacy workflows. ABHunter maintains sub-second search time across increasing dataset sizes (~38,000 to ~217,000 sequences), whereas conventional Pandas/CSV workflows experience increased latency with larger data volumes (0.072 ± 0.005 vs. 9.2 ± 0.20 seconds). The resulting speedup factor increases progressively, reaching a 127-fold speedup compared to compressed CSV formats at the tested limit *(Search parameters: Full Results (no limit), IGHV: 3-30)*. **(B)** Sub-linear scaling across the billion-sequence Unpaired Heavy Chain corpus. To evaluate the system’s performance at larger scale, search times up to the limit of ~1.48-billion-sequence were measured, showing that the total query time remains under 1.8 ± 0.03 seconds for statistical summaries and 2.2 ± 0.06 seconds for full result retrieval (100 sequences) for the tested queries, while the number of retrieved hits increases to over 76 million. This disproportionately small increase in latency relative to database growth demonstrates sub-linear scaling efficiency of the framework *(Search parameters: Statistics Only & Full Results (limit=100), IGHV: 3-30)*. **(C)** Search time is primarily driven by retrieved result volume rather than search complexity. ABHunter maintains highly responsive searches even as query specificity increases, with latency scaling primarily according to retrieved result volume and motif complexity. This was assessed by measuring latency across four levels of complexity (from “Simple” to “Very Complex”) under different retrieval modes. For statistical summaries, a “Simple” search was completed in 3.7 ± 0.29 seconds, while a “Very Complex” search took 20.8 ± 1.28 seconds. Phase timing shows that this increase is dominated by result-fetch SQL (materializing rows from disk), while subject-stats SQL remains comparatively stable across retrieval limits *(Search parameters: Simple: V gene, Medium: V gene + J gene, Complex: V gene + J gene + CDRH3 length, Very Complex: V gene + J gene + CDRH3 length + CDRH3 motif, see methods for further information)*.

#### 2.2.3 Sub-linear scaling enables real-time statistical profiling across billions of sequences

To evaluate ABHunter’s AntibodySearchEngine at larger scale, performance was benchmarked across subsets of the unpaired heavy-chain dataset up to ~1.48-billion-sequences (**Figure 4B**; **Supplementary Table 6**). At the largest measured size, the engine generated statistical summaries in 1.8 ± 0.03 seconds and retrieved a representative set of 100 filtered records in 2.2 ± 0.06 seconds. Query time increased with database size, but sub-linearly over the measured range (~106 million to ~1.48 billion sequences, power-law exponent b ≈ 0.45). A standard Pandas/CSV workflow completed the same gene-usage search on a small subset (~380,000 sequences) in 16.7 ± 0.58 seconds, with peak memory of ~1.8 GB. Further naive in-memory Pandas materialization could not be extended towards full-corpus scale on our tested hardware. Progressive loading was terminated at ~120 million sequences when process resident set size reaches a 6 GB safety ceiling (measured failure mode) (**Supplementary Table 7**). By using projection pushdown on disk-backed Parquet files, ABHunter avoids the prohibitive memory overhead of traditional row-oriented formats.

#### 2.2.4 Search speed maintains second-scale responsiveness as query complexity increases

The influence of query specificity and materialization depth was assessed on the full unpaired heavy chain corpus of ~1.58 billion sequences (**Figure 4C**; **Supplementary Table 8**). As search parameters transition from “Simple” gene filters to “Very Complex” multi-parameter queries (see methods for detailed search parameters), latency for statistical summaries increases from 3.7 ± 0.29 to 20.8 ± 1.28 seconds, reflecting the computational cost of intensive regular expression matching and string-pattern processing. The results distinguish between computational search speed and data materialization overhead. Retrieving 100-record subsets adds negligible latency relative to statistics-only mode (e.g., Simple: 4.1 ± 0.21 seconds vs. Very Complex: 23.8 ± 0.63 seconds). Large-scale exports are constrained by result materialization from disk: reconstructing one million records requires 13.5 ± 0.60 seconds for “Simple” queries and 117.8 ± 1.10 seconds for “Very Complex” queries, with the increase dominated by result-fetch SQL rather than subject-stats aggregation.

#### 2.2.5 Cold-start and concurrency behavior remain practical on a single workstation

To test whether reported latencies depend on a pre-warmed cache, we compared cold-start searches (fresh AntibodySearchEngine/DuckDB session) with warm-cache repeats on the full unpaired heavy-chain corpus. For Simple and Complex queries in statistics-only and limit=100 modes, cold and warm times remained within the same order of magnitude (e.g., Simple statistics-only: 8.2 ± 0.72 seconds cold vs 7.2 ± 0.38 seconds warm and Complex limit=100: 14.1 ± 0.06 seconds vs 14.3 ± 0.16 seconds), corresponding to cold/warm ratios of 1.0-1.13. Thus first-query overhead is modest relative to total search cost. Supplementary concurrency tests further show near-linear DuckDB thread speedup from 1 to 4 threads and nearly doubled throughput when two independent search processes run concurrently, with only a mild increase in per-query latency (**Supplementary Table 9**).

### 2.3 Statistically inferred pairing model enables the projection of pairing probabilities onto the unpaired dataset

Since natively paired sequences represent only 0.03% of the dataset (**Figure 3**) and originate from a limited cohort of six donors (**Supplementary Figure 1**), we developed a statistically inferred pairing model to transform the pairing observations into an estimated functional repertoire map for the ~1.86-billion unpaired sequences. To assess whether the paired subset can serve as a statistical proxy for this inference, we compared gene usage, intra-chain pairing, and CDR3 length distributions with the unpaired subset and with published healthy-donor baselines.

Both independent gene usage and intra-chain V(D)J pairing frequencies (IGHV×IGHD, IGHV×IGHJ, IG(K/L)V×IG(K/L)J and IGHD×IGHJ) exhibited consistency between the paired and unpaired datasets. Independent gene usage was assessed both at family level (e.g., IGHV3) and family-gene level (e.g., IGHV3-1). At the broader family level, usage (Pearson r≥0.91, all *p* < 0.001) and ranking order (Spearman ρ≥0.93, all *p ≤* 0.005) were highly correlated (**Figure 5A**). This agreement was maintained at the family-gene resolution across all heavy V, D and J genes (Pearson r≥0.92, all *p* < 0.001, Spearman ρ≥0.90, all *p ≤* 0.005) and for light V and J genes (Pearson r≥0.88, all *p* < 0.001, Spearman ρ≥0.92, all *p ≤* 0.001) (**Supplementary Figure 2**; **Supplementary Table 10**). Cosine similarity and Jensen-Shannon Divergence confirmed the structural concordance of the V(D)J pairings (**Supplementary Figure 3**; **Supplementary Table 11**). CDRH3 and CDRL3 length distributions showed the same paired-unpaired alignment, both dataset-wide and across V-gene families with the paired CDRH3 shifted by one amino acid (see use-case 3 and **Supplementary Figure 4**). The observed family-level gene usage mirrors previously reported healthy human repertoire analyses. For the heavy chain, IGHV3 was found to be the most frequent IGHV gene family, followed in prevalence by IGHV4 and IGHV1 (21, 67, 91, 92), alongside dominant usage of IGHD3 (93) and IGHJ4 (4, 91). Similarly, IGKV1 and IGKV3, along with IGLV1 and IGLV2, emerged as the most commonly used variable gene families across the light chain repertoires, followed by the usage of IGKV2 and IGLV3, respectively (21, 67, 68). These comparisons, together with their alignment with published healthy-donor gene-usage baselines, indicate that the paired and unpaired datasets share gene-usage and intra-chain pairing structure. Concordant CDR3 length distributions further support this similarity but are not used by the inferred pairing model. This provides support for the use of the paired subset as a statistical proxy in the inferred pairing model, while still being limited by the six-donor paired cohort.

**Figure 5:**
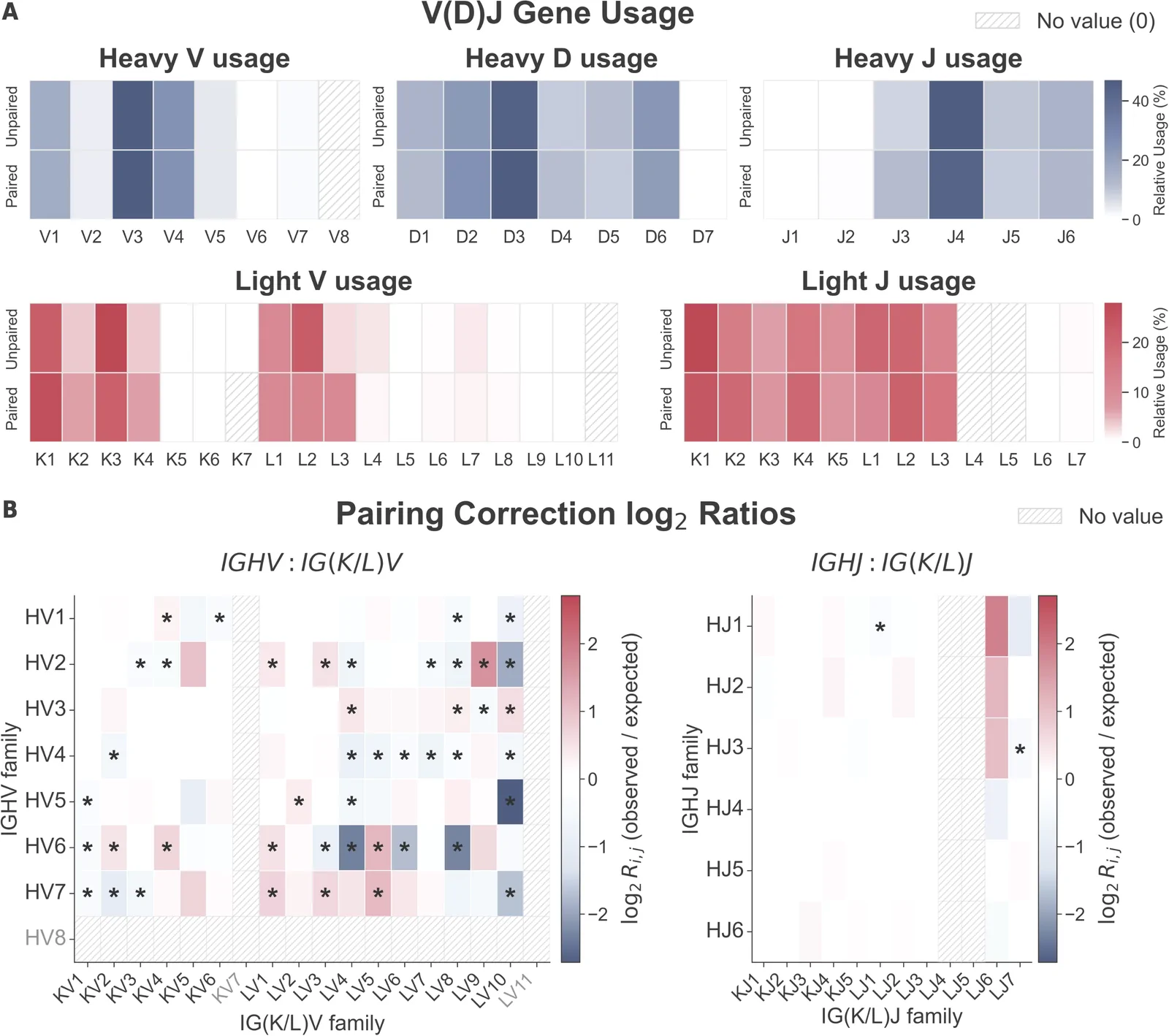
Statistical validation and normalization revealed non-random pairing preferences across the human antibody repertoire. **(A)**: Genetic Correlation across Paired and Unpaired dataset. Relative V(D)J gene family usage shows high correlation between the paired and unpaired datasets across both gene segment usage (Pearson r≥0.91, *p* < 0.001) and rank order (Spearman ρ≥0.93, *p ≤* 0.005). **(B)** Pairing Correction Ratios reveal significant non-random associations towards certain heavy-light chain pairings. Pairing Correction Ratio (R_i,j_​) for heavy-light chain V (**(B)**, left) and J [**(B)**, right] genes, displaying the relative deviation from random pairing. Significant individual pairings are marked with an asterisk [* Bonferroni-corrected *p* < 0.05 and fold-change threshold (see Methods)].

Despite this representativeness, the raw pairing frequencies (P_obs_) are inherently influenced by the overall abundance of individual segments (**Supplementary Figure 5**). By reflecting the relative deviation from random pairing rather than absolute frequency, the calculated Laplace-smoothed correction ratio (R_i,j_) reduced sparse-cell zero artifacts and normalized these frequency effects to quantify the degree of preference in antibody assembly (**Figure 5B**; see Methods and **Supplementary Method 1**). This provides a computational basis for predicting heavy- or light-chain pairing preferences for given V and/or J genes within the ABHunter web interface. The derived pairing correction ratios indicated that immunoglobulin assembly exhibits distinct pairing biases across both V and J gene segments. Specific IGHV​:IG(K/L)V and IGHJ : IG(K/L)J combinations exhibit marked positive bias (R_i,j_​>1), while other combinations show negative bias (R_i,j_​<1) despite high individual gene abundance (**Figure 5B**).

To determine if these observed deviations represent true biological phenomena, we performed a Pearson chi-square (χ^2^) test of independence. While this test yielded mathematically significant p-values for these deviations (IGHV×IG(K/L)V: *p*≪0.001; IGHJ​×IG(K/L)J​: *p*≪0.001), such metrics are inherently inflated by the large dataset size. To evaluate true biological magnitude of this non-randomness, we quantified the overall effect sizes, which were notably small (Cramér’s V: 0.053 for V, 0.025 for J; Total Variation Distance: 0.044 for V, 0.021 for J, **Supplementary Table 12**). This indicated that while global chain pairing remains predominantly stochastic to maximize antibody diversity, specific inter-chain combinations are driven by localized, deterministic pairing biases (64, 70, 94–96).

To assess the robustness of these pairing biases, we performed a leave-one-donor out cross-validation (**Supplementary Tables 13**, **14**). Recalculating the correction ratios (R_i,j_) from five-donor subsets demonstrated high agreement with the full six-donor matrix (mean Spearman ρ = 0.95 for V families, ρ = 0.89 for J families), which demonstrates that the global pairing biases are stable and not disproportionately driven by any single donor. However, predicting a single held-out donor’s specific R_i,j_ matrix yielded moderate agreement among the most deeply sequenced donors (ρ = 0.54-0.55 for V, ρ = 0.29-0.35 for J) and weaker agreement across all donors (mean ρ = 0.34 for V, ρ = 0.26 for J), reflecting the high inter-individual variability inherent to isolated human repertoires. These R_i,j_ matrices provide the computational basis for predicting likely heavy- or light-chain partner candidates for given V and/or J genes within the ABHunter web interface.

### 2.4 Use cases

Using ABHunter, we demonstrate that the selected OAS subset can be treated as a granularly searchable database for specific genetic and structural features, as described above. While the framework offers a baseline for repertoire characterization through standardized plots for every search, the following use cases demonstrate how real-time accessibility enables researchers to address a wide range of different targeted immunological questions: The quantification of bnAb precursor frequencies to evaluate potential targets for germline-targeting vaccine design (2.4.1), rational antibody design using the native repertoire context to scaffold isolated features and rescue orphan chains (2.4.2) and mapping of global structural and genetic landscapes of the human antibody space through billion-scale repertoire analysis (2.4.3).

#### 2.4.1 Use Case I - germline-targeting vaccine design: evaluating bnAb precursor frequencies to identify viable immunogen targets

The development of successful germline-targeting vaccines against highly genetically diverse pathogens relies fundamentally on targeted naïve B-cell precursors existing at viable baseline frequencies within the target population (49–63). Traditionally, transitioning from an identified bnAb sequence to a population-wide census required piecing together disparate computational steps, ranging from extracting genetic and structural features to writing custom scripts to execute searches across datasets like the OAS. ABHunter eliminates this bioinformatic barrier, allowing users to determine precursor frequencies in real-time either by directly searching known antibody features or utilizing the integrated NCBI IgBLAST module to extract V(D)J genes, CDR lengths, and motifs directly from raw nucleotide sequences. The search enables researchers to quickly model different levels of stringency to quantify their impact on population accessibility.

To validate our framework, we investigated the precursor frequency of well-characterized HIV-1 bnAbs, which are established germline-targeting vaccine design targets (**Figure 6A** HIV panel) (54, 60–62). By searching their genetic key features as previously reported (**Supplementary Table 15**), ABHunter determined precursor frequencies (normalized as the number of hits per million total sequences) that closely align with established literature values, demonstrating a median absolute log_10_ difference of 0.08 (a 1.20-fold difference) and maintaining a maximum difference of 0.23 (a 1.71-fold difference) across all evaluated targets (**Supplementary Tables 16**, **17**). This was further validated through Spearman rank correlation (ρ=1, *p ≤* 0.001) and Bland-Altman analysis (**Supplementary Figure 6**), which revealed a mean bias of +0.05 log_10_ hits per million, with 95% limits of agreement ranging from -0.21 to 0.32 log_10_. All evaluated targets fell within these limits of agreement, confirming that ABHunter-derived precursor frequencies exhibit minimal systematic bias when reproducing established repertoire frequencies. As our validation matched the reported statistical measures and zero-hit inclusion criteria of each reference study, the minor remaining quantitative discrepancies primarily reflect underlying structural differences, such as overall database scale and the handling of inter-study donor duplicates. By successfully reproducing these population-level metrics, ABHunter demonstrated its ability to accurately and reliably quantify precursor frequencies within the human antibody repertoire.

**Figure 6:**
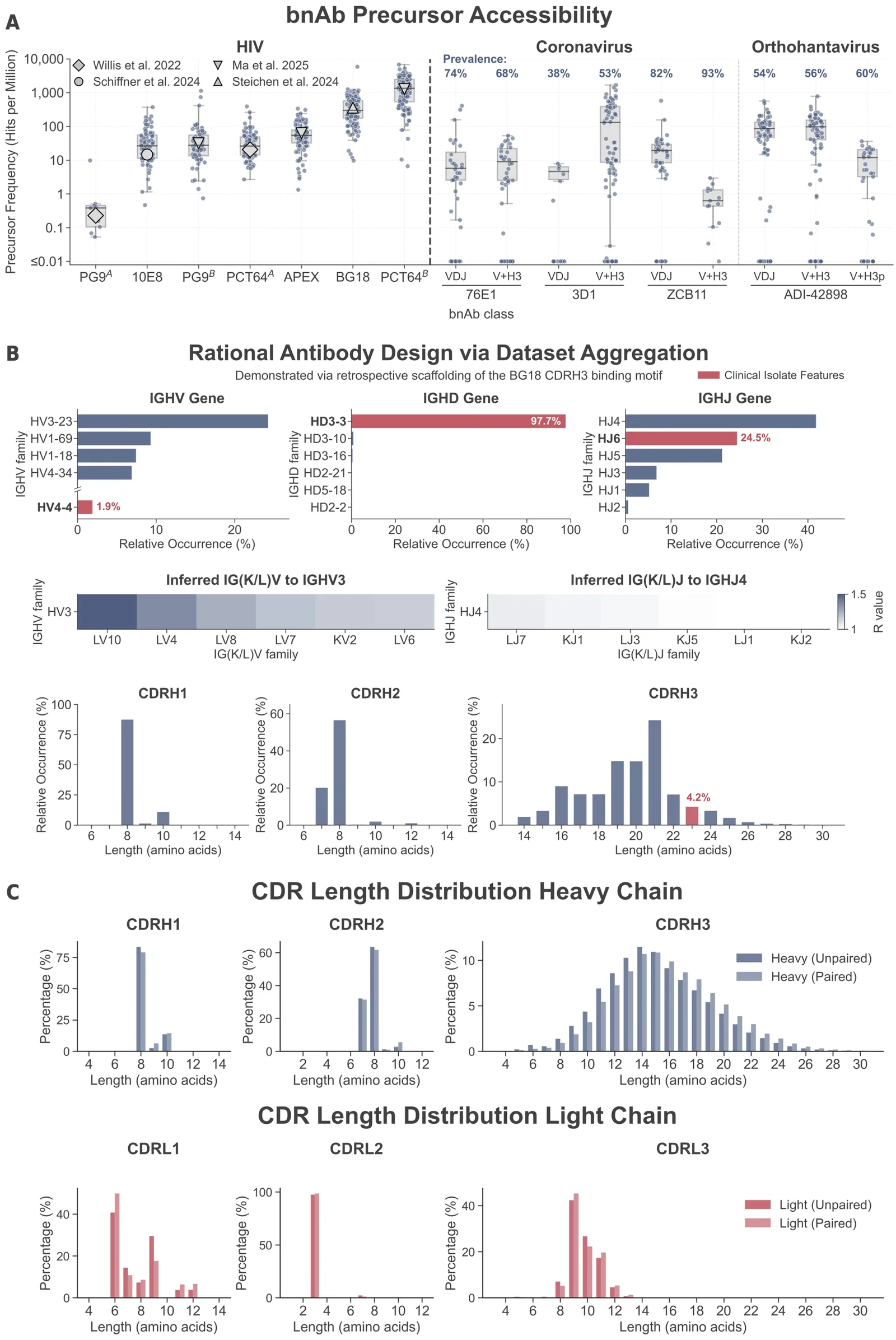
Results for use cases: bnAb Precursor Frequency Analysis, Rational Antibody Design, and Global Repertoire Characterization using ABHunter. **(A)** Reproducing established HIV-1 bnAbs precursor frequencies and identifying potential targets for germline-targeting vaccines. Precursor frequencies shown as hits per million (log_10_ scale) for individual donors. Boxes show interquartile ranges with median-line. HIV panel: Well-characterized HIV-1 bnAb classes, with superimposed symbols indicating previously reported values *[Diamond: Willis 2022* (60)*, Circle: Schiffner 2024* (54)*, Downward triangle: Ma 2025* (61)*, Upward triangle: Steichen 2024* (62)*].* Coronavirus/Orthohantavirus panel: Precursor frequency for selected Coronavirus and Orthohantavirus bnAbs under varying search stringency parameters *(VDJ: V, D and J genes, V+H3: V gene and CDRH3 motif without precise positional placement, V+H3p: V gene and CDRH3 motif with positional placement).* **(B)** Identifying biologically favored baseline scaffolds for the BG18 binding motif by aggregating dataset-wide feature distributions. Aggregated distributions resulting from the BG18 CDRH3 motif search, displaying the relevant feature of the clinical isolate in red alongside their occurrence frequencies as percentages. Across the retrieved population, the plots display the relative usage of IGHV, IGHD, and IGHJ genes (top), the corresponding IG(K/L)V and IG(K/L)J genes predicted by the statistically inferred pairing model (middle), and the length distributions for CDRH1-3 (bottom). **(C)** Defining intrinsic structural boundaries by extracting dataset-wide CDR length profiles. Normalized frequency plots for CDR1, CDR2, and CDR3 lengths for both heavy (top) and light (bottom) chains across the paired and unpaired datasets.

We next applied our platform to evaluate several Coronavirus (97–99) and Orthohantavirus (100) bnAb classes, quantifying their precursor frequency and assessing their viability as potential germline-targeting vaccine targets for the first time. Unlike the HIV-1 validation benchmarking, where no sequence-depth filter was applied to facilitate direct comparison to literature values, the evaluation of these novel targets utilized a Poisson-based limit of detection filter to account for donor-specific sequencing-depth (see Methods) and included zero-hit donors to report prevalence (**Figure 6A** Corona/Orthohantavirus panel, **Supplementary Table 18**). To evaluate the statistical sensitivity of the thresholding parameters, we performed a parallel sensitivity analysis by repeating all searches without applying the sequence depth filter. Omitting this filter expectedly deflated observed donor prevalence across all novel targets because of the inclusion of shallowly sequenced donors where zero hit designations were driven by undersampling rather than true biological absence. Crucially, the relative hierarchy and overall distribution profiles of the evaluated target lineages remained highly consistent between the thresholded and unthresholded analyses, demonstrating that our biological classifications are robust to the chosen depth parameters (see **Supplementary Figure 7**; **Supplementary Table 19**).

As increasingly restrictive search parameters inherently reduce identified frequencies, determining which structural and genetic features are essential for broad neutralization versus merely preferred presents a critical challenge. To address this, we performed different search strategies for each bnAb class, focusing either on the heavy chain encoding VDJ genes, which often dictate epitope binding (search “VDJ”), or utilizing a more restrictive search incorporating the IGHV gene and key CDRH3 residues without or with precise positional placement (searches “V+H3” or “V+H3p”, respectively). These H3 key residues were identified either in the respective publications, or by us through structural analyses (76E1 PDB: 7X9E, ZCB11 PDB: 7XH8, ADI-42898 PDB: 7QQB) (**Supplementary Table 20**). The results revealed highly variable distribution patterns across the analyzed bnAb classes, with precursor frequencies ranging from tightly clustered donor cohorts (e.g., ADI-42898, setting: “VDJ”) to dispersed distributions (e.g., 3D1, setting: “V+H3”), reflecting the natural demographic variation of different antibody features within the evaluated naïve repertoire. Furthermore, utilizing different search stringency strategies revealed frequency shifts within single bnAb classes. For example, the median precursor frequency for ZCB11 dropped from 19.35 per million in the VDJ search to 0.63 per million in the more stringent V+H3 motif search. Conversely, we observed an increase for 76E1, reflecting that its key CDRH3 motif can be generated by multiple IGHD and IGHJ combinations, ultimately capturing more matches than a search focusing solely on specific encoding germlines.

As evaluating a bnAb as a potential germline-targeting vaccine target requires moving beyond a single universal precursor frequency threshold, we analyzed these novel findings by comparing their precursor frequency distributions alongside their corresponding observed donor prevalence. For several bnAb classes, such as 3D1 (98) or ADI-42898 (100), the data revealed broad, highly variable profiles characterized by a substantial number of zero-hit donors who met our depth threshold, indicating that a biologically relevant fraction of the population inherently lacks the requisite precursors under these defined settings (representing true biological non-responders). Specifically, ADI-42898 precursors were absent in a substantial portion of deeply sequenced donors, showing an observed donor prevalence of 54% in the VDJ search (58 out of 108 donors positive, median: 83.93 HPM) and a similar 60% prevalence under the restrictive V+H3p search, though with a drastically reduced median frequency of 11.97 HPM (25 of 42 donors positive). Similarly, the 3D1 class showed an observed donor prevalence of 38% in the VDJ search (6 of 16 positive donors, median: 4.73 HPM), which rose to 53% prevalence (64 of 121 positive donors, median: 131.81 HPM) in the V+H3 search. In contrast, applying this combined prevalence and distribution analysis highlights 76E1 (97) and ZCB11 (99) as exhibiting highly favorable precursor accessibility profiles across the analyzed cohorts, avoiding the cohort-level elicitation gaps seen in other bnAb classes.

While population-wide precursor accessibility is an essential starting point, high precursor frequency alone does not guarantee priming success or vaccine efficacy. Crucial multiparametric factors, such as the initial immunogen affinity and minimal genetic requirements for neutralization often necessitate further characterization (e.g., paratope mapping (101–103) to identify key-contact residues). Nonetheless, by evaluating the overall prevalence and distribution dynamics across healthy donors, the platform enables researchers to rapidly screen and prioritize candidate lineages based on their starting genomic accessibility in the human population.

#### 2.4.2 Use Case II - rational antibody design: using native repertoire context to scaffold isolated features and orphan chains

Antibody design frequently begins with isolated genetic or structural features, such as functional peptides or minimal structural loops, discovered through high-throughput screening (104), cross-species isolation (105), or computational design (106, 107). To successfully translate these partial sequences into viable biotherapeutics, one approach involves grafting these paratopes into the CDRs of stable antibody frameworks (63, 105, 108–111). Selecting structurally compatible, naturally occurring scaffolds for this process provides a robust starting point for rational engineering, mitigating the risk of instability and reduced therapeutic efficacy associated with entirely arbitrary feature combinations (19, 63–69, 112, 113). ABHunter facilitates this approach by contextualizing searched features within their native environment. Rather than evaluating combinations in isolation, the platform aggregates search results into various plots that illustrate how sequence features naturally co-occur. Coupled with the statistically inferred pairing model to predict cross-chain dependencies, the framework visualizes a structural landscape that guides users toward biologically grounded architectures prior to experimental synthesis. By providing these dataset distributions directly within the interactive web interface, the platform empowers researchers to visually analyze complex scaffolding pathways in real-time.

To demonstrate this capability under a simulated *de novo* design scenario where only the binding paratope is known, we performed a retrospective scaffolding analysis on the defining CDRH3 motif of the HIV-1 bnAb BG18 (62). Although the original publication provided a broader motif for precursor frequency evaluations (as mirrored in Use Case I), we applied their more restrictive CDRH3 motif definition to determine its naturally preferred structural and genetic baselines. This search-specific result aggregation generated diverse feature distributions (97,844 hits), allowing for a direct comparison against the known genetics of the clinical isolate and the bnAb class (**Figure 6B**; **Supplementary Table 21**).

Analyzing the gene usage distributions revealed a rigid requirement for the IGHD3–3 gene, which is identical to the clinically isolated BG18 antibody. This contrasts with the broader repertoire flexibility observed across the surrounding IGHV and IGHJ frameworks. While the clinically isolated BG18 antibody utilizes the IGHV4–4 and IGHJ6 genes, ABHunter identified IGHV3–23 and IGHJ4 as the most statistically enriched scaffold across the healthy human repertoire. Rather than conflicting, this genetic divergence aligns with known experimental structural biology of the BG18-class, which demonstrates that the binding paratope is predominantly IGHD gene-driven and natively tolerant of diverse surrounding IGHV and IGHJ gene families (62). Crucially, the highest alternative scaffolds identified by our platform (IGHV3–23 and IGHV1-69) are directly validated by independent experimental reports. Wet-lab characterizations by Steichen et al. demonstrated that engineered BG18-like variants designed to utilize alternative common gene families (specifically swapping IGHV4–4 for IGHV3–23 or IGHV1-69) retained potent binding affinities to the germline-targeting vaccine candidates (59). Utilizing the highest-ranking IGHV​ and IGHJ​ genes to evaluate cross-chain dependencies, the statistically inferred pairing model suggests a predictive preference for the IGLV10 light-chain family (R = 1.5), with no distinct bias for any specific IG(K/L)J​ partner (R≈1). While the original clinically isolated bnAb utilizes different genes (IGLV3–25 and IGLJ3), BG18-like type I lineages are known to natively tolerate diverse lambda and kappa chains, confirming that the specific pairing of the clinical isolate's defining paratope is not a rigid biological requirement for functional binding (62). Beyond this genetic framework, the CDR length distributions revealed that, while a 21 amino acid CDRH3 predominates, the healthy repertoire also successfully covers longer, naturally occurring configurations. This includes both the ≥22 amino acid lengths defining the BG18 class and the precise 23 amino acid lengths utilized by the clinical isolate. Occurring at a hit rate of 4.2%, this low frequency reflects the natural rarity of atypically long CDRH3 loops typical for several bnAbs (114), highlighting the fundamental elicitation bottleneck that germline-targeting vaccines are designed to overcome.

By successfully mapping the required IGHD-gene anchor and CDRH3 loop length, while revealing multiple statistically enriched options for the surrounding IGHV and IGHJ frameworks, ABHunter equips researchers with diverse, naturally occurring alternative pathways to scaffold this motif. This scaffolding capability is validated by the empirical findings of Steichen et al., who confirmed that our predicted heavy-chain alternatives, including IGHV3–23 and IGHV1-69, maintain potent functional binding (59). This demonstrates that our framework successfully maps biologically favored baseline scaffolds for a given feature, such as a motif, by converting dataset-wide distributions into usable reference values for downstream prospective testing.

#### 2.4.3 Use Case III - billion-scale repertoire analysis: executing multi-parameter scans to map global structural and genetic architectures

While the ABHunter web interface provides access to several repertoire statistics, the scale of the dataset enables the evaluation of complex immunological questions that require flexible, multi-parameter scans, such as identifying disease-associated repertoire deviations (63, 115). By installing the framework locally, researchers can execute SQL queries directly against the entire database.

This approach allows for the deep characterization of genetic and structural landscapes and their relationships, analyses previously unattainable as traditional workflows often restrict studies to isolated subsets due to the prohibitive memory requirements, limiting the ability to extract global biological signals. By bypassing these computational constraints, the local framework enables researchers to execute diverse, multi-parameter queries across the entire database in seconds. To demonstrate this capability and validate the dataset as a robust physiological reference, we performed several global analyses of the immune repertoire landscape, yielding results that align with previous reports on the healthy human baseline. We calculated length distributions for all six CDRs, revealing highly conserved signatures for CDR1 and CDR2, while CDRH3 exhibits the characteristic near-Gaussian distribution centered at 14 amino acids (1, 4, 116) (**Figure 6C**). Notably, the CDRH3 length distribution within the natively paired subset remains consistent with the broader unpaired corpus, though it exhibits a shift toward a slightly longer mean length (15 amino acids), which likely represents a sampling artifact from the restricted six-donor pool (**Supplementary Figure 1**). We further utilized the framework to map intra-chain gene pairing frequencies (**Supplementary Figure 3**), alongside family-specific CDR3 length shifts that reveal family-dependent variations in length distributions (**Supplementary Figure 4**). Together, these findings align with previous reports indicating that deterministic genetic biases govern the repertoire even prior to pairing (48, 72, 74–77). Ultimately, these dataset-wide V(D)J audits provided both the gene usage analysis (on family and family-gene level) discussed earlier, as well as the inter-chain co-occurrence data necessary for the statistically inferred pairing model (**Figure 5**; **Supplementary Figure 5**).

By executing these diverse, multi-parameter scans in a matter of seconds, the framework demonstrates a capacity for billion-scale statistical modeling that would otherwise be computationally prohibitive. Rather than restricting users to a pre-defined search mask, the direct SQL interface provides fast, local and unconstrained access to the complete dataset. This empowers researchers to easily design and run bespoke queries, rapidly compiling custom tables of gene frequencies or CDR length distributions to explore the healthy human OAS subset on their own terms.

## 3 Discussion

Identifying the natural frequencies and distributions of antibody features is essential for advanced vaccine development and therapeutic discovery, but extracting actionable biological insights from massive sequencing data remains a bottleneck. Here, we developed ABHunter, a web-accessible and locally installable framework that transforms the ~1.86-billion-sequence healthy human OAS subset into a real-time, searchable database. ABHunter’s closest functional counterpart is cAb-Rep (84). Like ABHunter, cAb-Rep combines a graphical interface, a command-line client, cloud access, and local installation with regex-based motif search. Two concrete differences remain. cAb-Rep’s corpus consists of 267.9 million V(D)J heavy chain and 72.9 million VJ light chain transcripts. ABHunter instead draws on the full ~1.86-billion-sequence healthy-human OAS subset, several orders of magnitude larger in both donor and sequence coverage. cAb-Rep also constrains search by CDR3 length alone. ABHunter allows independent length and regex-motif constraints on CDR1, CDR2, and CDR3 for the heavy and light chains separately, combined with allele-level V(D)J filtering and isotype-specific search. Approximate identity-search tools solve a related but distinct problem. KA-Search (82) and ABDiver (85) both rank candidate sequences by percentage identity to a query over a fixed positional mask. Optimized to exhaustively search any antibody region against all known antibodies (82) or V-region profiling (85), they lack ABHunter’s ability to search user-defined regex motifs with wildcards, and a graphical user interface. CompAIRR (83) addresses a third task. It quantifies sequence overlap and clonotype sharing between two user-supplied repertoires, not a fixed reference database. This makes it complementary to ABHunter’s search function rather than a replacement for it (**Supplementary Table 1**).

Using our framework, we analyzed the dataset and obtained results that align with previous reports on the healthy human immune repertoire. We could demonstrate that the observed V(D)J gene usage (21, 67, 68, 91–93) (**Figure 5A**; **Supplementary Figure 2**), as well as the identified intra- and inter-chain (72, 74–76) co-occurrence biases (**Supplementary Figures 3**, **5**, respectively), mirrored established physiological baselines. Similarly, analyzing global CDR lengths highlighted the expected structural duality between rigid CDR1/CDR2 and variable CDRH3 loops (**Figure 6C**) (1, 4, 116). We also found family-specific shifts in CDRH3 and CDRL3 lengths, indicating that germline gene usage constrains loop architecture (**Supplementary Figure 4**) (48, 77). These analyses enable the comparison of baseline repertoire and property distributions of antibodies in healthy humans against specific repertoire deviations. Characterizing these deviations provides potential clinical proxies for germline-based disease diagnosis across infectious, autoimmune, and B-cell malignant conditions (63, 115). Separately, defining the typical boundaries of antibody structures allows the rational discovery of functional outliers, such as extended CDRH3 loops often considered to have broadly neutralizing potential (114). Ultimately, rather than providing an exhaustive biological analysis of the entire dataset, these evaluations establish a foundational baseline that demonstrates the platform’s capabilities.

Having demonstrated the statistical representativeness of the paired against the unpaired dataset, we justified the usage of identified inter-chain pairing frequencies as a proxy for the entire unpaired repertoire, overcoming the limitation that paired sequences constitute only 0.03% of the dataset (**Supplementary Figure 1**). After correcting for individual gene abundance, we observed distinct deviations from stochastic assembly. Because maintaining a highly diverse antibody repertoire relies fundamentally on random V(D)J chain pairing, the global deviation from strict independence is expectedly small, as reflected by the low effect sizes (Cramér’s V ≤ 0.053; TVD ≤ 0.044). However, within this overarching stochasticity, certain specific inter-chain gene family combinations were significantly favored or disfavored despite their individual baseline frequencies (**Figure 5B**). Aligning with previous reports (64, 71, 94–96), these localized deterministic biases and non-random pairings likely reflect strict structural constraints and selection pressures imposed both during early V(D)J recombination and subsequent chain pairing (**Supplementary Figure 8**). Reportedly, these constraints are driven by fundamental physical forces at the VH-VL interface, including overarching electrostatic charge complementarity between domains, specific packing of conserved interface residues, and direct structural cross-talk between framework regions and opposing CDR3 loops. While machine learning models can predict heavy-light pairing by mapping complex structural relationships (e.g., the known influences of contact residues between CDRs and framework regions) (117–121), our approach intentionally focuses on broad, gene-level statistical observations rather than predicting full-length chain partners. By extracting these deterministic biases from the paired subset, the platform can rapidly and probabilistically suggest favored V and J partner genes following a user search. However, applying these insights to the broader unpaired platform necessitates careful interpretation. The underlying paired dataset originates from a limited, highly uneven cohort of only six donors, where three individuals contribute over 97.7% of the sequences, which inherently introduces demographic and sampling biases. As highlighted by our leave-one-donor-out analysis, individual repertoires exhibit high inter-individual variability, meaning a single donor’s specific pairing can be highly distinct. Yet, when aggregated, a more stable, overarching biological signal emerges that remains robust regardless of which donor is excluded. Consequently, while our inferred pairing provides a valuable, evidence-based approximation of population-level structural biases, it must be interpreted as a probabilistic guide rather than an absolute rule, and it cannot entirely substitute for large-scale paired sequencing across diverse demographics.

By evaluating established germline-targeting vaccine targets with our framework, we accurately reproduced HIV-1 precursor frequencies, confirming our underlying data architecture provides a statistically robust baseline (**Figure 6A**). We also applied the platform to novel Coronavirus and Orthohantavirus targets and identified that the mean numerical frequencies can mask population-level vulnerabilities. The discovery of broad zero-hit donor profiles for certain targets indicates inherent elicitation gaps within the population. As demonstrated by our sensitivity analysis, the implemented sequencing-depth threshold provides statistical confidence that these zero-hit donors are not solely driven by the undersampling artifacts common in repertoire sequencing (**Supplementary Figure 7**; **Supplementary Table 19**) (122–124). Conversely, the consistent, cross-donor prevalence of lineages like 76E1 (97) and ZCB11 (99) identifies them as promising starting points for germline-targeting immunization strategies. Furthermore, the observed frequency shifts between purely gene-based and structure-informed searches demonstrate that evaluating potential bnAb targets based solely on encoding germlines is insufficient. For example, CDRH3 motif constraints alter precursor accessibility, either restricting frequencies or expanding them through alternative recombination pathways (**Figure 6A**; **Supplementary Table 20**). Consequently, identifying key structural requirements, such as CDR length or the amino acid residues critical for binding, through computational modeling (101), empirical paratope mapping (e.g., X-ray crystallography and chemical cross-linking) (102), or a combination of these methods (103) may be a prerequisite for accurate profiling. However, we note that identifying a target with favorable population-wide prevalence does not independently guarantee its success as a germline-targeting vaccine target, as precursor frequency and immunogen affinity are interdependently limiting factors for B-cell competitive fitness and germinal center recruitment (50, 56). At the low precursor frequencies typically found in physiological human repertoires, variables such as immunogen affinity thresholds, clonal lineage size and anatomic compartmentalization are critical parallel requirements for successful priming. If starting affinity is too low, even highly abundant precursors cannot be successfully recruited and will be outcompeted by non-target endogenous B-cells during germinal center reactions. While these complex biological constraints must be evaluated downstream, ABHunter provides the essential, quantitative population-level baseline. By delivering several descriptive metrics (e.g., prevalence, mean, median, and interquartile range), our platform provides researchers with the ability to rapidly evaluate potential targets based on their fundamental genomic accessibility before undertaking downstream design efforts, preventing the optimization of germline-targeting immunogens for B-cell precursors that are inherently absent or severely restricted across the human population.

We also applied the ABHunter framework to the evaluation of rational scaffolding strategies. When engineering therapeutics around isolated features, our framework challenges the assumption that a clinical isolate represents the optimal scaffold. As it represents solely one solution from a specific patient, mapping statistically enriched, natively occurring scaffolds across the repertoire may provide evolutionary alternatives (**Figure 6B**) (59, 63, 68, 110, 113). Furthermore, analyzing global distributions allows researchers to evaluate the publicity of a defined feature. Antibody properties shared across hundreds of unrelated donors indicate a high degree of systemic immunological tolerance, whereas rare or unique properties (such as V alleles or CDR motifs) carry a higher risk of immunogenicity, such as eliciting anti-drug antibodies (69, 112). We acknowledge that our scaffolding evaluation is retrospective and focused on a single antibody class as a proof of concept. Although direct wet-lab validation of our proposed scaffolds was beyond the scope of this study, our results are supported by the independent experimental characterization of the selected bnAb class by Steichen et al., as the enriched alternatives we identified closely align with known BG18-like variants previously shown to exhibit potent affinity and neutralizing activity (59, 62). Additionally, mapping the feature distributions of known isolates illuminated biological bottlenecks, e.g., demonstrating the intrinsic disfavoring of a defining long CDRH3 loop by the recombination machinery illustrates why certain antibodies are difficult to elicit, thereby necessitating the requirement of germline-targeting strategies. By utilizing the natural repertoire as a pre-validation blueprint to evaluate structurally compatible genes and features under specific constraints, ABHunter allows researchers to isolate natively validated starting scaffolds before investing computational time into structural modeling platforms like AlphaFold or Rosetta, or undertaking wet-lab experiments.

While ABHunter provides a robust analytical framework, its current implementation is subject to several notable limitations. Primarily, while alternative unpaired and paired sequence repositories exist (70, 71, 78), often with partial data overlap, our exclusive reliance on the OAS for its standardized data structure inherently restricts the platform’s scope. Crucially, as demographic factors shape the epitope specificity of the human antibody repertoire (125), our results reflect the specific cohorts within the OAS and do not equally represent all human populations. Secondly, this dataset reliance results in a predominance of unpaired sequences, yielding biological likelihoods rather than direct functional evidence. However, as heavy-light chain-preserving sequencing techniques mature (31, 32) and the respective datasets expand, the predictive accuracy of our statistically inferred pairing model will scale accordingly, allowing for the potential incorporation of CDR3 physicochemical complementarity or machine-learning architectures (117–120). Furthermore, repertoire analysis accuracy remains intrinsically tied to the germline annotation quality provided by Olsen et al. and Kovaltsuk et al., especially regarding highly mutated sequences (126). Finally, the physical export and downloading of high-volume records remain governed by standard disk I/O limitations.

In conclusion, we present ABHunter, an open-access tool that bridges the traditional divide between ease of use and computational depth by unifying an intuitive web interface with the analytical power of an optimized framework. By executing real-time, multi-parameter scans to evaluate precursor frequencies, profile natively tolerated scaffolds, and analyze dataset-wide repertoire patterns, we demonstrate how this tool makes the massive healthy-human OAS database intuitively searchable. Ultimately, this continuously updated framework offers an evidence-based foundation to support downstream engineering efforts as the curated OAS repertoire expands.

## 4 Methods

### 4.1 Data acquisition and dataset pre-processing

Paired (heavy and light chain) and unpaired datasets from healthy, non-vaccinated human individuals were retrieved from the Observed Antibody Space (OAS) database (January 2026). The downloaded compressed, row-oriented CSV files, comprising 1,863,783,517 total datapoints, were transformed into a column-oriented Apache Parquet format utilizing ZSTD compression. During this pre-processing phase, the data were filtered to retain essential immunological columns (**Supplementary Table 22**). CDR lengths were calculated as the character length of the amino acid sequence fields (*cdr1_aa*, *cdr2_aa* and *cdr3_aa*, with *_heavy* and *_light* suffixes for paired data) following IMGT standards and appended as dedicated columns (*cdr1_length*, *cdr2_length* and *cdr3_length*, with *heavy* and *light* suffixes for paired data) to the respective sample files. Sample metadata were extracted, stored in individual Parquet file headers and aggregated into a centralized metadata.parquet file for each chain/isotype sub-directory (**Supplementary Table 23**). The final directory structure preserved a one-to-one mapping with the original OAS partitioning by chain type (Heavy, Light, Paired) and isotype (Heavy: Bulk, IGHA, IGHD, IGHE, IGHG, IGHM, Light: Bulk, Paired: All). The complete data schema for sequence and metadata files is documented in **Supplementary Tables 24** and **25**, respectively.

#### 4.1.1 Quality control and sequence filtering

While primary quality filtering was executed upstream by the OAS authors to exclude stop codons, frame-shifts, and sequences with incomplete CDR boundaries, additional curation filters were applied via the scripts during ABHunter database construction. Heavy chain variable regions (IGH) were required to contain valid, non-null IGHV, IGHD, and IGHJ germline annotations, whereas light chain variable regions (IGK/IGL) were required to contain valid IG(K/L)V and IG(K/L)J annotations. Locus cross-validation was performed, and records with mismatched data (e.g., a V-gene call mapped to a *d_call* field) were discarded. For unpaired datasets, redundant transcripts containing identical variable-region amino acid sequences originating from the same study and donor were collapsed into single entries. For paired datasets, this duplicate checking and collapsing was executed based on the unique combination of both heavy and light chain variable-region amino acid sequences within each study and donor. The entire workflow, including data collection, format conversion and all downstream quality control filtering, was automated using *scripts/update_from_oas.py*, which queries the OAS server for matching studies and *scripts/convert_to_parquet.py*, which reformats and filters the downloaded records.

#### 4.1.2 Germline annotation and standardization

Germline gene assignments (*v_call*, *d_call*, and *j_call*, with *_heavy* and *_light* suffixes for paired data), sequence alignments, numbering annotations and pre-segmented CDR amino acid sequences were inherited directly from the OAS dataset, where they were generated upstream via the Antibody Numbering and Antigen Receptor Classification (ANARCI) tool (127) applying the IMGT gene nomenclature and numbering scheme (128, 129). Calculated CDR lengths were derived from the pre-segmented amino acid fields, thereby conforming to IMGT boundary definitions.

### 4.2 Database integration and AntibodySearchEngine

Database operations were executed using DuckDB with an in-memory connection. Connection parameters are set at initialization and listed in **Supplementary Table 26**. Prepared Apache Parquet files were registered as DuckDB views via *read_parquet()*. Per chain/isotype *metadata.parquet* files were combined into a single metadata view and joined to sequence data on *file_path*. In addition to a global view over all Parquet files, ABHunter registered three chain-specific DuckDB views by assigning each file to Heavy, Light, or Paired according to its directory path, and routing search queries to the corresponding view. No secondary database indexes were created, as search performance relied on Parquet columnar storage, DuckDB predicate pushdown on parquet statistics and restriction of scans to chain-specific file subsets. Separately, paired versus unpaired column naming (*v_call* vs *v_call_heavy/v_call_light*) was detected from the Parquet schema to determine SQL column references and valid search modes. Query execution and data access were managed through a custom Python API, the AntibodySearchEngine. It mapped user-defined search parameters (V(D)J gene calls, CDR lengths, CDR motifs) to SQL *WHERE* clauses, gene filters as *LIKE* patterns, length filters as numeric conditions, and motif filters as RE2 regular expressions (motif syntax and similarity rules are described in the method section “CDR length and motif matching”). Searches were executed either as subject-level aggregations (*GROUP BY subject*) or as full sequence retrievals with an optional row limit and column subset. Per-donor hit counts were merged with *total_sequences* from metadata, and hits-per-million values were computed before returning results as a tuple of sequence dataframe, statistics dataframe, and summary dictionary. For unpaired searches, precomputed V/J pairing frequencies could be loaded separately and appended to result rows.

### 4.3 Web application

ABHunter was implemented as a Streamlit web application with four modules (Database Search, IgBLAST, Inferred Pairing, Imprint). On the Database Search page, users selected chain/isotype sub-repositories by checkbox, while selected paths were passed as *data_dirs* to AntibodySearchEngine at initialization in the main application process. Search criteria (V(D)J genes, CDR lengths, CDR motifs) were entered through chain-specific forms and executed in unpaired or paired mode. Searches were submitted to a background ProcessPoolExecutor with two worker processes. Each worker initialized an independent in-memory DuckDB connection using the parameters listed in **Supplementary Table 26**. Submitted searches were queued on the executor’s default unbounded queue when both workers were busy with no query timeout applied. Search status was polled asynchronously at 2 second intervals until the worker process returned results (via *st_autorefresh*). Database initialization, plot generation, and export preparation were executed synchronously in the main Streamlit process. After search completion, results were rendered as aggregate and per-donor hits-per-million statistics, distribution plots, and inferred pairing heatmaps using Plotly. Plot data were loaded in a separate query on the session-scoped search engine, retrieving only columns required for visualization (V/D/J gene calls, CDR length, CDR sequence), thereby bypassing the serialization overhead of full-record datasets. Results and exports (statistics CSV, frequency tables, full results tables, FASTA and plot archives) could be generated on demand. Depending on the deployment (Docker/server or local installation) and the total export file size, file delivery was dynamically routed to be served via a dedicated Nginx download endpoint, loaded into memory or saved to local disc (**Supplementary Table 27**). The web application has been tested for compatibility with the following web browsers: Safari 26.2 by Apple, Firefox 147.0.3 by Mozilla Corporation, and Google Chrome 145.0.7632.76 by Google LLC.

#### 4.3.1 Gene pattern matching

V, D, and J gene inputs were translated into SQL *LIKE* patterns, supporting family (3), gene (3–23), and allele (3-23*01) notation with optional chain prefixes. Family- and gene-level patterns were bounded to prevent partial matches (e.g., 3 to IGHV3-*, 3–30 to IGHV3-30*). Light-chain inputs without prefix were matched against both lambda and kappa loci, while L- or K-prefixed inputs restricted matching accordingly. Comma- or pipe-separated gene lists were split and combined with *OR* within each gene column and non-empty V, D, and J fields were combined with *AND*. Inputs were validated for permitted characters, gene number ranges, and disallowed gene calls.

#### 4.3.2 CDR length and motif matching

CDR length filters were applied to precomputed cdr1/2/3_length columns. Length inputs were parsed into SQL conditions supporting fixed values (e.g., 5), ranges (e.g., 2-5), and inequality operators (>, >=, <, <=), independently for CDR1, CDR2, and CDR3 on heavy and/or light chains (**Supplementary Table 28**). CDR motif filters were evaluated against cdr1/2/3_aa columns using the DuckDB RE2 regex engine (~ operator). User motif strings were translated into RE2-compatible patterns according to a restricted motif syntax (**Supplementary Table 29**). Characters outside this syntax were escaped and not accepted as input. Patterns supported single-residue wildcards, variable-length gaps, explicit residue alternatives, and fixed or ranged length quantifiers. Defined motif positions (literal amino acids and explicit residue classes) were matched exactly unless a per-CDR mismatch budget *k* > 0 was specified. *k* was capped at the number of defined positions in that motif and encoded Hamming substitution only. At most *k* defined positions could differ from the query and insertions and deletions were not allowed. In that case, up to *k* defined positions were allowed to differ from the query (wildcard tokens, including., *, and length quantifiers, were not counted toward *k*). With Similarity mode off, each mismatched position could be any amino acid. With Similarity mode on, substitutions at mismatched positions were restricted to predefined chemical-property groups (aliphatic, acidic, basic, aromatic, hydroxyl, amide, unique, **Supplementary Table 30**). Similarity mode without mismatches (*k* = 0) therefore reduced to exact matching.

#### 4.3.3 Frequency by donor plot and results

Donor-level precursor frequencies were visualized in the web application using interactive boxplots with individual donors as points (Frequency by Donor). For each donor, hits per million (HPM) were calculated as hits divided by the donor’s total sequencing-depth and multiplied by 10^6^. Frequencies of ≤0.01 per million, including zero-hit donors, were binned at a baseline value of ≤0.01 on a log_10_ axis. Which donors were included in the visualization and the corresponding statistical calculations was controlled by two independent, user-configurable filtering options: the sequence depth filter, which is detailed in the “Sequencing-depth filter and sensitivity analysis” section below, and a zero-hit toggle that allows for inclusion or exclusion of zero-hit donors. A frequency summary table was computed from the active donor subset, reporting the sequence-depth threshold, the number of displayed donors, the absolute count and percentage of zero-hit donors (or the count of excluded donors when zero-hit display was disabled) and the resulting HPM distribution as mean, median, and interquartile range (IQR, Q1 and Q3).

#### 4.3.4 IgBlast

The standalone NCBI IgBLAST application (igblastn v1.22.0) was integrated into the web application via Python subprocess invocation (130, 131). Reference human germline nucleotide sequences (IGH, IGK, and IGL loci) were obtained from the IMGT reference directory website, reformatted, and compiled into separate BLAST databases for V, D, and J genes (132). User-provided heavy- or light-chain nucleotide sequences are queried against these reference databases. Heavy chains are assigned V, D, and J genes in a single run. Light chains are queried against both kappa and lambda germline databases, and the locus with the highest V-gene bit score is retained. Gene calls are extracted from the tabular IgBLAST output (up to three hits per gene), while CDR motifs and lengths are derived from the IMGT-numbered alignment. Selected annotations are passed via Streamlit session state to populate the database search mask.

#### 4.3.5 Automatic database updating

The pipeline scripts (*scripts/update_from_oas.py* and *scripts/convert_to_parquet.py*) utilized for initial database construction were designed to support incremental updates. The scripts evaluate OAS server timestamps against the local database to identify and download only newly deposited studies. If new paired datasets are processed during an update, *scripts/inferred_pairing_model.py* is automatically triggered to recompute the frequency matrices for the statistical co-occurrence model. On the abhunter.org server, this synchronization workflow is scheduled via an automated cron job on the first day of every month, but the same pipeline scripts can be run manually by users to keep their localized databases synchronized with the OAS. To ensure version tracking across these updates, all exported search results feature an embedded timestamp indicating the OAS database snapshot present at the time of query.

### 4.4 Storage requirements and computational performance benchmark

On-disk storage requirements for the selected OAS subset were determined empirically for all files matching the dataset selection criteria (healthy, non-vaccinated human) using *scripts/calculate_file_sizes.py.* Via the OAS database index (created with scripts/update_from_oas.py), we identified 5,617 sequence files (5,052 heavy-chain, 474 light-chain, and 91 paired-chain files). For each file, the corresponding csv.gz file was downloaded from OAS, and its on-disk size was recorded. Each file was then decompressed to raw CSV format and the resulting file size was measured. Finally, the uncompressed data were converted to Parquet format retaining all OAS data columns, with study metadata stored in Parquet file-level metadata and ZSTD compression applied, after which the on-disk Parquet file size was also recorded. The optimized database was generated as explained in “Data Acquisition and Pre-processing the Dataset” section. Total storage requirements for each format were obtained by summing file sizes across all files in the subset (**Figure 3**; **Supplementary Table 4**).

Query performance was benchmarked by comparing AntibodySearchEngine (DuckDB on Parquet), a file-by-file Pandas workflow on uncompressed CSV, and the same workflow on gzipped CSV (.csv.gz). On a progressively scaled benchmark subset (37,505-217,443 sequences; search: IGHV3-30), wall-clock time was measured over five runs after two warmup runs (median reported, mean ± SD used for figures and tables), and hit-count equivalence was verified across all three implementations (**Supplementary Table 5**).

AntibodySearchEngine was further benchmarked on progressive subsets of the unpaired heavy-chain two database warmup runs at 9 progressive test points in statistics-only and full-result (limit=100) modes (Search “IGHV3-30”) (**Supplementary Table 6**). Scaling with database size was summarized by fitting a power law t ~N^b^ via ordinary least squares on log-transformed mean wall-clock times and sequence counts over the progressive heavy-chain subset.

The same IGHV3–30 Pandas/CSV workflow was also timed on the full unzipped CSV benchmark subset, with process resident set size recorded in parallel. Scaling of naive Pandas materialization beyond this subset was probed on progressive unpaired heavy-chain Parquet subsets ordered by increasing size. Each file was read with Pandas, IGHV3-30-matching rows were retained in memory, and filtered frames were concatenated. Each size point was run in a monitored subprocess and terminated if process resident set size exceeded a predefined ceiling of 6 GB (≤ 25% of the 32 GB test machine) (**Supplementary Table 7**). Query complexity was assessed on this full database at four levels: Simple (IGHV3-30), Medium (IGHV3-30 + IGHJ4), Complex (IGHV3-30 + IGHJ4 + CDRH3 length 15 aa), and Very Complex (IGHV3-30 + IGHJ4 + CDRH3 length 15 aa + CDRH3 motif AR*), each executed in statistics-only mode and in full-result mode with limit=100 and limit=1,000,000, with wall-clock time decomposed into results-fetch SQL and subject-stats (GROUP BY) SQL (**Supplementary Table 8**). Cache and concurrency behavior were additionally characterized for Simple and Complex queries in statistics-only and full results (limit=100) modes: Cold starts (fresh engine) versus warm searches on the same engine, DuckDB intra-query thread scaling, and full-database scale (**Supplementary Table 9**). All benchmarks were run on an Apple MacBook Pro (CPU: M1 Pro with 10 cores, RAM: 32 GB LPDDR5, disk: SSD) under macOS Tahoe 26.2.

### 4.5 Repertoire analysis

Repertoire-level immunological summaries were computed directly from ABHunter Parquet databases using DuckDB, separately for unpaired heavy, unpaired light, and paired partitions. All repertoire analyses followed IMGT standard, using IMGT gene nomenclature, unique numbering and CDR-IMGT region definitions (128, 132).

V(D)J gene usage: For each partition, V, D (heavy and paired heavy only), and J gene assignments were counted at family and family-gene level, retaining only chain-appropriate genes (IGHV/IGHD/IGHJ for heavy, IG(K/L)V and IG(K/L)J for light). Allele suffixes were stripped prior to counting. Distal gene designations (e.g., IGKV3D-20) were merged with the corresponding gene (here IGKV3-20). Family and family-gene identifiers follow IMGT nomenclature, corresponding to IMGT subgroup (e.g., IGHV1) and IMGT gene (e.g., IGHV1-2), respectively. J Genes lack a gene-number suffix, so family and gene identities match. Usage at both resolutions was expressed as the percentage of all valid gene assignments within each chain, gene type and dataset (heavy, light, paired). Missing expected families (IGHV1–8, IGHD1–7, IGHJ1–6; IGLV1–11, IGKV1–7, IGKJ1–5, IGLJ1–7) were assigned count zero. At family-gene level, V and D counts were retained per gene. For each V and D comparison, the gene set was the union of genes observed in unpaired or paired data, and genes present in only one dataset were assigned count zero in the other. Human J genes have no IMGT gene number distinct from the set identifier, so family- and gene-level J analysis used the same identifiers, with missing genes assigned count zero. Agreement between unpaired and paired usage profiles was assessed separately for heavy-chain V, D, and J genes and for light-chain V and J genes, at both resolutions. For each comparison, usage percentages were arranged as paired vectors of equal length. Family-level vectors were heavy V: n = 8, heavy D: n = 7, heavy J: n = 6, light V: n = 18, light J: n = 12. Family-gene-level vectors were V: n = 64, heavy D: n = 26, heavy J: n = 6, light V: n = 62, light J: n = 12. Heavy-chain correlations compared unpaired heavy-chain sequences and paired heavy-chain sequences. Light-chain correlations compared unpaired light-chain sequences with paired light-chain sequences. Pearson correlation and Spearman rank correlation were computed with *scipy.stats.pearsonr* and *scipy.stats.spearmanr*, respectively, across corresponding family or family-gene vectors. Spearman and Pearson statistics as well as exact *p* values are reported in **Supplementary Table 10**. In the main text, *p* values are summarized with conventional thresholds for readability.

CDR length distributions: CDR1, CDRH2/CDRL2, and CDRH3/CDRL3 lengths were counted across all sequences in each partition. Length frequencies were converted to percentages within each chain type and dataset.

Intra-chain gene co-occurrence: V-J, D-J (heavy only), and V-D (heavy only) co-occurrence tables were built by counting sequence-level gene pairs, filtering to valid chain-specific genes, aggregating to gene family, and row-normalizing so each V or D family summed to 100% across partner families. Analyses were performed separately for unpaired heavy, unpaired light, and paired heavy/light. Intra-chain co-occurrence profiles were compared between unpaired and paired repertoires at the gene-family level (heavy: V-D, V-J, D-J & light: V-J, κ/λ-restricted). Count vectors were normalized to probabilities. Cosine similarity and Jensen-Shannon Divergence values were calculated using *scipy.spatial.distance.cosine* and *scipy.spatial.distance.jensenshannon*, respectively, and are reported in **Supplementary Table 11**.

Gene family vs. CDR3 length associations: To assess CDR length variation by germline context, each sequence was cross-tabulated by gene family and CDR3 length (n = sequence count). Analyses were run separately for unpaired and paired data. Only sequences with non-empty CDRH3/L3 amino acid sequence and valid IGHV/IGHD/IGHJ/IG prefixes were included. Cross-tabs were exported as gene family x CDR3 length count tables. For each gene family, the weighted median CDR3 length was computed. The same procedure was applied to heavy D-gene families (IGHD1-7) and heavy/light J-gene families.

### 4.6 Statistical co-occurrence model

From the paired heavy-light repertoire, IGHV : IG(K/L)V and IGHJ : IG(K/L)J pairs were counted after excluding entries with invalid family annotations (heavy V: IGHV*; light V: IGKV*/IGLV*; heavy J: IGHJ*; light J: IGKJ*/IGLJ*), yielding 568,137 sequences. Subgroups, alleles, and normal and distal sub-loci (e.g., IGKV2 and IGKV2D) were collapsed to gene families (V genes: family name taken before the first “-” with trailing distal-gene “D” removed from chain names where present, J genes: truncation at the first “*”). This family-level aggregation was implemented to maintain compliance with Cochran’s rule for the chi-square test of independence, as subgroup-level granularity generated excessive matrix sparsity (16% absolute zero cells and 27% of cells with expected counts <5). Analysis used complete family grids: IGHV (IGHV1–IGHV8) x IG(K/L)V (IGKV1–IGKV7 + IGLV1–IGLV11) (144 pairs) and IGHJ (IGHJ1–IGHJ6) x IG(K/L) (IGKJ1–IGKJ5 + IGLJ1–IGLJ7) (72 pairs). Missing family combinations were assigned count zero. Families with a total raw count of zero were then removed (IGHV8, IGKV7 and IGLV11, IGLJ4 and IGLJ5), leaving matrices of 7×16 = 112 IGHV: IG(K/L)V pairs and 6×10 = 60 IGHJ: IG(K/L)J pairs. Pairing counts at the family level are shown in **Supplementary Tables 31**, **32**.

For pairing-correction factors, Laplace smoothing with additive constant α = 1 was applied to every cell of each active matrix (n′_i,j_ = n_i,j_ + 1, with effective total N′ = N + α × m, where m is the number of active cells). For each family pair (_i,j_), the observed paired frequency was P_obs_(_i,j_) = n′_i,j_/N′. Marginal frequencies P(_i_) and P(_j_) were computed from the same Laplace-smoothed paired data, and the expected frequency under random pairing was P_rand_(_i,j_) = P(_i_) × P(_j_). The pairing correction factor was R_i,j_ = P_obs_(_i,j_) ÷ P_rand_(_i,j_), with R _i,j_ > 1 (log_2_ R _i,j_ > 0) denoting positive pairing bias and R _i,j_ < 1 (log_2_ R _i,j_ < 0) denoting negative pairing bias relative to independence (**Supplementary Method 1**; **Supplementary Tables 33**, **34**).

Under the null hypothesis of random IGHV : IG(K/L)V and IGHJ : IG(K/L)J pairing (independence of heavy- and light-chain family assignment), deviation from random pairing was assessed with a Pearson chi-square (χ^2^) test of independence (*scipy.stats.chi2_contingency*) following Cochran’s Rule (133) (no cells with expected count <1 and cells with expected count <5 under 20%), comparing observed family co-occurrence counts to counts expected under independence. Effect size was quantified as Cramér’s V (*scipy.stats.contingency*). Departure of the observed joint distribution from the independence expectation was additionally summarized by the total variation distance (TVD). Exact *p* values, together with the χ^2^ statistic, degrees of freedom, number of cells with expected counts <5, number of cells with expected counts <1, minimum expected count, TVD and Cramér’s *V*, are reported for each contingency table in **Supplementary Table 12**. In the main text, *p* values are summarized with conventional thresholds for readability.

*Post hoc* analysis of individual family combinations used adjusted standardized residuals (134) to obtain two-tailed p-values. Multiple-testing correction used the Bonferroni procedure with threshold α/m (α = 0.05, m = number of active cells). Fold change was defined as FC(_i,j_) = n_i,j_ ÷ E_i,j_, where E_i,j_ is the expected count under independence. Combinations were classified as enriched if Bonferroni-significant, the adjusted residual was positive, and FC ≥ 1.20, and as depleted if Bonferroni-significant, the adjusted residual was negative, and FC ≤ 0.80.

For each family pair (_i,j_​), a 95% confidence interval for the Laplace-smoothed pairing correction factor R _i,j_​ was obtained with the Garwood Poisson interval for an observed-to-expected count ratio, using the same Laplace-smoothed counts and smoothed expected count as for R. The lower and upper bounds were obtained from chi-square quantiles at α = 0.05 with degrees of freedom 2O′ (lower) and 2O′ + 2 (upper), each divided by 2E′, where O′ is Laplace smoothed observed count n′_i,j_ and E′ Laplace-smoothed expected count N′ x P_rand_(_i,j_). Corresponding intervals on the log_2_ scale were obtained by transforming these endpoints (**Supplementary Tables 33**, **34**).

#### 4.6.1 Leave-one-donor-out analysis for R

R_i,j_ was calculated after excluding each of the six paired donors in turn, using the same Laplace-smoothed estimator as the full dataset (R_i,j_ = P_obs_​(_i,j_)/P_rand_(_i,j_)) on the family grid (112 IGHV×IG(K/L)V and 60 IGHJ×IG(K/L)J). For each left-out donor d, Spearman ρ was computed between the vectorized five-donor matrix R^(d)^ and (i) the full six-donor matrix R and (ii) the held-out donor’s own matrix R^(d)^. Each comparison was made on all informative cells (112 for V, 60 for J) and on raw-positive cells (n>0) (**Supplementary Tables 13**, **14**).

### 4.7 Precursor frequency analysis

For each bnAb precursor definition (**Supplementary Table 15**), search criteria were compiled from literature or, where these were incomplete, from structural analyses of the bnAb-antigen complex, to identify CDRH3 residues relevant to epitope contact. Missing germline assignments were identified with NCBI IgBLAST [https://www.ncbi.nlm.nih.gov/igblast/ (130)].

Each search was executed in the ABHunter web application against the unpaired heavy-chain IgM subset and exported per-donor statistics (subject, total_sequences_donor, hits) to CSV. Where distinct search definitions for the same bnAb class were derived from different publications, each was run and evaluated separately. For complementary criteria defining a single bnAb class within a study (long and short CDRH3 criteria for 10E8), sequence hits were summed on a per-donor basis to represent the total precursor frequency. Per-donor precursor frequency was calculated as hits per million (HPM = hits/total_sequences_donor x 10^6^) and transformed to log_10_(HPM) for plotting. Donors with HPM ≤ 0.01 were assigned log_10_ = -2 (tick label “≤0.01” in **Figure 6A**). For each bnAb class, donors were shown as strip points overlaid on a boxplot.

#### 4.7.1 Validation against HIV bnAbs

Searches were performed using the reported bnAb-class constraining features, and the resulting precursor frequencies were evaluated using the same statistical measure (mean, median, geomean) applied in each respective reference study (**Supplementary Tables 16**, **17**). No sequencing-depth threshold was applied and zero-hit donors were excluded. Published precursor frequencies were overlaid as marker symbols at log_10_(literature HPM), and agreement with ABHunter was quantified as |log_10_(HPM_literature) − log_10_(HPM_abhunter)|. Statistical concordance between reported and ABHunter-derived HIV-1 bnAb precursor frequencies was evaluated using Spearman’s rank correlation (using *scipy.stats.spearmanr*) and Bland-Altman analysis (**Supplementary Figure 6**) (135).

#### 4.7.2 Sequencing-depth filter and sensitivity analysis

For a given search, the pool-level hit frequency was computed as f = Σhits/Σtotal_sequences_donors, where Σtotal_sequences_donors was the total number of sequences across all donors. To establish a 95% confidence limit of detection under a Poisson distribution (122, 123), individual donors were included only if their donor-specific sequencing-depth was ≥ [3/f], including donors that met this depth criterion but had zero hits, except where otherwise indicated. A parallel sensitivity analysis was performed by repeating all Coronavirus and Orthohantavirus bnAb precursor searches and frequency calculations without this sequencing-depth threshold, incorporating all available donors. Absolute prevalence rates, median frequencies, and relative target rankings were calculated for both the filtered and unfiltered datasets (**Supplementary Table 19**; **Supplementary Figure 7**).

#### 4.7.3 Prevalence calculations

For Coronavirus and Orthohantavirus bnAbs, observed donor prevalence was calculated as the percentage of qualifying deep-sequenced donors with at least one precursor match, with 95% confidence intervals estimated using the Wilson score method for binomial proportions (136). Median precursor frequencies and their corresponding 95% confidence intervals were calculated exclusively from positive responders (excluding zero-hit donors) using a non-parametric exact binomial method based on sorted order statistics (136) (**Supplementary Table 18**).

### 4.8 Software and deployment

ABHunter was developed in Python (3.11) using Streamlit (1.50.0) for the web interface, DuckDB (1.4.0) for query execution, PyArrow (21.0.0) for Parquet access, and Pandas (2.3.3) for result handling. NCBI IgBLAST was integrated by subprocess invocation of the standalone igblastn application (1.22.0) with bundled human germline databases (130, 131). The application was deployed as a Docker container (Python 3.11-slim). Statistical tests were performed in Python using SciPy (1.17.1) (137).

### 4.9 Data visualization

Molecular graphics were performed with UCSF ChimeraX (Version 1.10), developed by the Resource for Biocomputing, Visualization, and Informatics at the University of California, San Francisco (138, 139). All plots were generated using Matplotlib (Version 3.7.2) (140).

## Data Availability

ABHunter is free, open-source software licensed under the MIT License. The source code and necessary configuration files are maintained in a public GitHub repository (https://github.com/SchiffnerLab/abhunter), which includes a lightweight test dataset for local validation. The ABHunter web application and server components are licensed under the GNU GPLv3 License and are publicly accessible at abhunter.org. The original raw antibody repertoire data utilized in this study were obtained from the public Observed Antibody Space (OAS) database (https://opig.stats.ox.ac.uk/webapps/oas/). To ensure both exact reproducibility of this study and access to ongoing OAS updates, the web server hosts two distinct database versions: a static snapshot of the January 2026 dataset analyzed in this manuscript, and a dynamic version that is automatically updated on the first day of every month. These updates are executed via the provided scripts/update_from_oas.py and scripts/convert_to_parquet.py files. Users wishing to build and update the database locally can run these same scripts to automate the full workflow of data collection, format conversion, and database population. Jupyter notebooks for reproducing all immunological analyses and figures reported in this paper, along with the generated data corresponding to the January 2026 OAS snapshot, are provided in the repository. Additionally, the repository includes interactive Jupyter notebooks detailing local command-line AntibodySearchEngine usage and DuckDB SQL examples. Any additional information required to reanalyze the data reported in this paper is available from the lead contact upon request.
