# Supplementary material for: Real-time exploration of the billion-scale human repertoire for precursor frequency analysis and rational antibody design
Source: Front Immunol. 2026 Oct 16;17:1958625. doi: 10.3389/fimmu.2026.1958625 (PMC13719593; doi:10.3389/fimmu.2026.1958625)
Supplement: Supplementary file 1 [file Supplementaryfile1.docx]

**Supplementary Material**

**Real-time exploration of the billion-scale human repertoire for precursor frequency analysis and rational antibody design**

Tom U. Schlegel^1^, Jannis von Blanckenburg de Riz^1,2^, Jakob R. Riccabona^1,2^, Franz Dietzmeyer^1^, Julia Koehler Leman^3^, Jens Meiler^1,2,4,5,6^, Clara T. Schoeder^1,2^, Torben Schiffner^1,7,8^

^1^ Institute for Drug Discovery, Faculty of Medicine, Leipzig University, Leipzig, Germany

^2^ Center for Scalable Data Analytics and Artificial Intelligence ScaDS.AI, Dresden/Leipzig, Germany

^3^ Open Molecular Software Foundation, Berkeley, CA, USA

^4^ School of Embedded Composite Artificial Intelligence SECAI, Dresden/Leipzig, Germany

^5^ Institute for Computer Science, Wilhelm Ostwald Institute for Physical and Theoretical Chemistry, University Leipzig, Leipzig, Germany

^6^ Department of Chemistry, Department of Pharmacology, Center for Structural Biology, Institute of Chemical Biology, Center for Applied Artificial Intelligence in Protein Dynamics, Vanderbilt University, Nashville, Tennessee, United States of America

^7^ Department of Immunology and Microbiology, The Scripps Research Institute, La Jolla, CA, USA

^8^ Vaccine and Immunotherapy Center, The Wistar Institute, Philadelphia, PA, USA

**Correspondance**

Torben Schiffner, Assistant Professor

Vaccine and Immunotherapy Center, The Wistar Institute

3601 Spruce St., Philadelphia, PA 19104, USA

Email: [tschiffner@wistar.org](mailto:tschiffner@wistar.org)

**Table of Contents**

**Supplementary Methods	4**

Supplementary Method 1: Detailed calculations for developing the statistical inferred pairing model	4

**Supplementary Figures	5**

Supplementary Figure 1: Donor distribution by chain type.	5

Supplementary Figure 2: Gene-level unpaired versus paired V(D)J usage.	6

Supplementary Figure 3: Intra-chain gene pairing frequencies across paired and unpaired datasets.	7

Supplementary Figure 4: CDR3 length distributions across heavy and light germline gene families.	9

Supplementary Figure 5: Observed pairing frequencies and difference matrices.	9

Supplementary Figure 6: Comparison of literature and ABHunter-derived precursor frequencies.	10

Supplementary Figure 7: Sensitivity analysis of the sequence depth filter on precursor frequencies and prevalence.	11

Supplementary Figure 8: Schematic representation of an antibody.	12

**Supplementary Tables	13**

Supplementary Table 1: Feature-Matrix Comparison between similar search and retrieval Tools	13

Supplementary Table 2: Example AntibodySearchEngine commands for local ABHunter queries	16

Supplementary Table 3: Example DuckDB SQL commands for local ABHunter queries	16

Supplementary Table 4: Storage footprint of OAS heavy, light, and paired datasets across file formats.	17

Supplementary Table 5: Progressive scalability versus legacy workflows	17

Supplementary Table 6: Progressive scalability on the unpaired heavy chain dataset	18

Supplementary Table 7: Progressive Pandas materialization	18

Supplementary Table 8: Search latency versus query complexity and retrieval mode	19

Supplementary Table 9: Cold versus warm cache behaviour and concurrency scaling	20

Supplementary Table 10: Correlation of intra-chain V(D)J gene usage between paired and unpaired dataset.	22

Supplementary Table 11: Similarity of intra-chain co-pairing frequencies between paired and unpaired datasets.	22

Supplementary Table 12: Statistical parameters for the heavy and light chain pairing model for both V and J.	23

Supplementary Table 13: Leave-one-donor-out Spearman correlation for the IGHV-IG(K/L) pairing-correction matrix R.	23

Supplementary Table 14: Leave-one-donor-out Spearman correlation for the IGHJ-IG(K/L)J pairing-correction matrix R.	24

Supplementary Table 15: Search parameters for determining bnAb precursor frequencies.	25

Supplementary Table 16: Comparison of selected reference bnAb frequencies from the literature.	27

Supplementary Table 17: Comparison of reported and ABHunter-derived HIV-1 bnAb precursor frequencies.	29

Supplementary Table 18: Statistical uncertainty intervals for novel precursor target evaluations	30

Supplementary Table 19: Sensitivity analysis of the sequence depth filter on precursor prevalence and frequency metrics.	31

Supplementary Table 20: Precursor frequencies for Coronavirus and Orthohantavirus bnAbs.	31

Supplementary Table 21: Genetic features of the clinical BG18 isolate and motif search parameter.	32

Supplementary Table 22: Retained and discarded columns during optimization.	33

Supplementary Table 23: Retained and discarded metadata during optimization.	36

Supplementary Table 24: Sequence column definitions.	37

Supplementary Table 25: Metadata column definitions	38

Supplementary Table 26: DuckDB configuration	38

Supplementary Table 27: Download handling for search results and plots under local install versus Docker/webserver deployment	39

Supplementary Table 28: Accepted CDR length search syntax	39

Supplementary Table 29: Mapping of ABHunter sequence-pattern input to RE2 regular expressions	40

Supplementary Table 30: Amino-acid property classes used for conservative substitution	41

Supplementary Table 31: Absolute counts of IGHV:IG(K/L)V gene family combinations.	41

Supplementary Table 32: Absolute counts of IGHJ:IG(K/L)J gene family combinations.	41

Supplementary Table 33: Laplace-smoothed pairing correction ratios (Ri,j) for IGHV:IG(K/L)V gene family combinations.	42

Supplementary Table 34: Laplace-smoothed pairing correction ratios (Ri,j) for IGHJ:IG(K/L)J gene family combinations.	45

**References	47**

# Supplementary Methods

## Supplementary Method 1: Detailed calculations for developing the statistical inferred pairing model

The following method describes the mathematical formulas used to quantify IGHV:IG(K/L)V germline co-occurrences and develop the statistical inferred pairing model. The identical approach was used to evaluate the IGHJ:IG(K/L)J pairing probabilities. Complete family grids were formed (IGHV1-IGHV8 × IGKV1-IGKV7 + IGLV1-IGLV11; IGHJ1–IGHJ6 × IGKJ1–IGKJ5 + IGLJ1–IGLJ7), with missing combinations set to count zero. Families with a total raw count of zero were removed, leaving the active matrices used below (IGHV × IG(K/L)V: 7 × 16 = 112 pairs; IGHJ×IG(K/L)J: 6×10 = 60 pairs).

1. **Laplace smoothing**

- Smoothed pair count n′i,j: raw count of paired sequences with V_H,i_ and V_L,j_ plus additive constant α = 1

$${n'}_{i,j}=n_{i,j} + a$$

- Effective total number of paired sequences after smoothing N′: raw total N plus α for each of the m active cells

$$N' = N +a \cdot m$$

2. **Paired V_H_ probability P(V_H_)**

- Frequency of each IGHV gene family V_H,i_ in the Laplace-smoothed paired data

$$P(V_{H,i})=\frac{{n'}_{i,\cdot}}{N'}=\frac{\Sigma_{j}{n'}_{i,j}}{N'}$$

3. **Paired V_H_ probability P(V_L_)**

- Frequency of each IGHV gene family V_H,i_ in the Laplace-smoothed paired data

$$P(V_{L,j})=\frac{{n'}_{\cdot,j}}{N'}=\frac{\Sigma_{i}{n'}_{i,j}}{N'}$$

4. **Observed paired IGHV:IGLV/IGKV probability P_obs_(V_H_,V_L_)**

$$P_{obs}(V_{H,i},V_{L,j})=\frac{{n'}_{i,j}}{N'}$$

- The absolute counts for each gene family pair are provided in Supplementary Table 31 (IGHV:IG(K/L)V) and Supplementary Table 32 (IGHJ/IG(K/L)J).

5. **Expected random paired V_H_:V_L_ probability P_rand_(V_H_,V_L_)**

- Product of the marginal frequencies, assuming independence

$$P_{rand}(V_{H,i},V_{L,j})=P(V_{H,i})\cdot P(V_{L,j})$$

6. **Pairing correction ratio R_i,j_**

$$R_{i,j}=\frac{P_{obs}(V_{H,i},V_{L,j})}{P_{rand}(V_{H,i},V_{L,j})}$$

- R_i,j_ > 1: preferred (positive bias)
- R_i,j_ < 1: disfavored (negative bias)
- R_i,j_ ≈ 1: close to random.
- Resulting rations (and 95% Garwood confidence intervals) are given in Supplementary Tables 33 and 34.

# Supplementary Figures

## Supplementary Figure 1: Donor distribution by chain type.

Bar plots display the absolute number of sequences assigned to individual donors across the Heavy, Light, and Paired dataset partitions. Shown are the top 25 donors with the highest sequence counts for each respective subset.

## Supplementary Figure 2: Gene-level unpaired versus paired V(D)J usage.

Relative V, D and J gene usage (%) from unpaired single-chain repertoires is compared with gene usage of paired heavy-light repertoires at family-gene resolution. Each point is one gene. Usage is the percentage of all valid gene assignments within that chain, gene type and dataset. Panels show heavy-chain V (n = 64), D (n = 26) and J (n = 6) genes and light-chain V (n = 62) and J (n = 12) genes. The dashed identity line indicates perfect agreement. Pearson r and Spearman ρ are annotated in each panel (see also Supplementary Table 10).

## Supplementary Figure 3: Intra-chain gene pairing frequencies across paired and unpaired datasets.

Relative intra-chain pairing frequencies for V×D, V×J, and D×J gene combinations are shown as heatmaps for both the unpaired (left) and natively paired (middle) sequence subsets, while the respective right panels visualize the absolute difference in relative frequencies between the two datasets. Cosine similarity and Jensen-Shannon Divergence of intra-chain pairing frequencies between the paired and unpaired datasets were also calculated (Supplementary Table 11).

## Supplementary Figure 4: CDR3 length distributions across heavy and light germline gene families.

Weighted occurrence distributions for CDRH3 and CDRL3 lengths are plotted across heavy and light gene families for the paired and unpaired datasets (V top, D middle, J bottom). The median of each population is marked. The x-axes display truncated ranges of 0-30 (CDRH3) and 5-17 (CDRL3) amino acids, representing >99.8% of the sequence repertoire.

## Supplementary Figure 5: Observed pairing frequencies and difference matrices.

**A:** Heatmaps display the raw observed pairing frequencies (P_obs_) for IGHVxIG(K/L)V (left) and IGHJxIG(K/L)J (right) combinations. **B:** Difference matrices visualize the observed minus expected frequencies (P_obs_–P_exp_) for the respective pairings.

## Supplementary Figure 6: Comparison of literature and ABHunter-derived precursor frequencies.

HIV broadly neutralizing antibody precursor frequencies (HPM, hits per million) from ABHunter are compared with published literature values on a log_10_ scale. ABHunter summary statistics were matched to the literature-reported metric (geometric mean for 10E8, median otherwise, see Supplementary Table 16). The dashed identity line indicates perfect agreement. The Spearman p value underflows double-precision floating point and is reported as p < 10^-308^. **Left:** Spearman rank correlation. The dashed identity line indicates perfect agreement. **Right:** Bland-Altman analysis: The solid horizontal line indicates the mean systemic bias. The panel shows the mean log_10_ difference and 95% limits of agreement (±1.96 SD).

## Supplementary Figure 7: Sensitivity analysis of the sequence depth filter on precursor frequencies and prevalence.

Precursor frequencies (Hits per million) for individual donors across all novel Coronavirus and Orthohantavirus target settings are compared on a log_10_ scale to demonstrate the impact of sequencing depth filtering (see also Supplementary Table 12). **Left**: Results obtained with the Poisson based limit of detection filter applied, which are identical to the novel target panels of Figure 6A. **Right**: Results obtained for the same target settings without applying any sequence depth threshold. In both panels, boxes represent the interquartile ranges and median lines calculated exclusively from positive responders, while zero hit donors are excluded from the box plots. Observed donor prevalence percentages are annotated as text directly above each column.

## Supplementary Figure 8: Schematic representation of an antibody.

**A:** Cartoon representation of an antibody variable domain (left: PDB accession: 5JW4) with a whole IgG structural model (right: PDB accession: 1IGT (1)). **B:** Illustration of V(D)J recombination which is encoding the variable segments of an antibody. V segments colored in green, D segments (heavy chain only) colored in red, C segments colored in blue.

# Supplementary Tables

## Supplementary Table 1: Feature-Matrix Comparison between similar search and retrieval Tools

| **Tool** | **Author** | **GUI for searches** | **CLI** | **Cloud version** | **Web-API** | **Speed (Website)** | **Custom data possible** | **Sequence Search** | **Sequence Region** | **Substring matching** | **length definitions** | **Gen Search** | **Isotype specific search** | **Local instal possible** | **Visualization** | **dataset** | **Pairing/Paired data support** | **Search Parameters** | **raw search/results downloadable/provided** | **Download DB** | **tool has own database** | **online version** | **registration mandatory** | **database maintained** | **Primary Focus & Target Application** |
| --- | --- | --- | --- | --- | --- | --- | --- | --- | --- | --- | --- | --- | --- | --- | --- | --- | --- | --- | --- | --- | --- | --- | --- | --- | --- |
| AHunter | Schlegel 26 | yes | yes | yes | no | seconds to minutes | yes, only locally | yes, CDR1-3 motif | CDR1-3 | yes | individually CDR1,2,3 | yes | yes | yes | yes | OAS | unpaired & paired | see manuscript | yes | yes | yes | yes | no | yes | see manuscript |
| ABSD (2) | Malesys 24 | yes | no | yes | no | seconds | yes, only locally | no | var. domain | yes | no | IGHV, IGKV/LV | no | yes | no | AbDb, AbPDB, CoV-AbDab, OAS, PDB, etc. | only paired | Species, Source | yes | yes | yes | yes | no | yes | Constructing paired datasets for AI |
| KA-Search (3) | Olsen 23 | no | yes | no | no | N/A | yes | yes, identity-based | var. domain, CDRs | no | toggle | no | no | yes | no | OAS | only unpaired | query sequence, mask, identity threshold | yes | yes | yes | no | no | yes | identity-based searching |
| CompAIRR (4) | Rognes 22 | no | yes | no | no | N/A | yes | yes | junction/CDR3 | only via indels | yes | sequence match, V, J | no | yes | no | user supplied | only heavy/light | explicit flags | yes | N/A | no | no | no | N/A | sequence overlap analysis |
| iReceptor (5) | Corrie 18 | yes | no | yes | yes | slow | yes | AA, substring | Junction/CDR3 | yes | CDR3 only | V, D, J Family/Allel | yes | yes | yes | AIRR Data Commons | AIRR Data Commons | Organism, Cell subset | yes | no | yes | yes | yes | yes | federated repository platform |
| PairedAbNGS (6) | Dudzic 25 | yes | no | yes (customer) | no | N/A | no | no | var. domain, CDR3 | no | no | V and J genes | yes | no | no | 58 bioprojects | only paired | Organism, B-cell, disease | no | yes | yes | no | yes | yes | paired sequence analysis |
| AbNGS (7) | Dudzic 24 | yes | no | yes (customer) | no | N/A | no | no | var. domain, CDR3 | no | no | V, D, J gene | yes | no | no | 220 bioprojects | only unpaired | Organism, disease | no | yes | yes | no | yes | yes | public CDR-H3 identification |
| cAb-Rep (8) | Guo 19 | yes | yes | yes | no | seconds | yes | yes | CDR3, var. domain | yes | yes | V and J gene | yes | yes | yes | 306 repertoires | only unpaired | Gene/allele, Isotype | yes | yes | yes | yes | no | no | explore curated diversity |
| OAS (9,10) | - | yes | - | yes | no | seconds | no | yes | - | - | - | yes | yes | no | no | 80 studies | unpaired & paired | Species, Chain, etc | yes | yes | yes | yes | no | yes | standardized repertoires |
| VDJServer (11) | Christley 18 | yes | no | yes | yes | slow | yes | no | var. domain, CDR3 | no | CDR3 length | yes | yes | yes | yes | AIRR Data Commons | unpaired | Project, MiAIRR metadata | yes | yes | yes | yes | yes | yes | analysis portal |
| ABDiver (12) | Młokosiewicz 22 | yes | no | yes | no | seconds | no | yes | var. domain, CDRs | yes | yes | V/J gene/allele | no | no | yes | OAS | only unpaired | Query sequence, V/J | yes | no | yes | yes | no | no | design & exploration |

Table showing the full Feature Matrix of Tools related to ABHunter.

## Supplementary Table 2: Example AntibodySearchEngine commands for local ABHunter queries

| **Search task** | **AntibodySearchEngine (Python)** |
| --- | --- |
| IGHV-gene search | engine.search(  chain_mode="heavy",  heavy_v="3-23"  ) |
| V + J + CDRH3 length + motif | engine.search(  chain_mode="heavy",  heavy_v="3-30",  heavy_j="4",  heavy_cdr3_length="15",  heavy_cdr3_motif="AR*"  ) |
| Paired IGHV×IG(K/L)V co-occurrence | (beyond ASE search mask) |

Representative AntibodySearchEngine *search()* calls for local command-line or notebook use. Parameters follow the same notation as the ABHunter web interface. Each call returns donor-level statistics and, optionally, sequence tables for downstream analysis and visualization.

## Supplementary Table 3: Example DuckDB SQL commands for local ABHunter queries

| **Search task** | **AntibodySearchEngine (Python)** |
| --- | --- |
| IGHV-gene search | SELECT COUNT(*) AS n  FROM read_parquet('data/Heavy/**/*.parquet')  WHERE v_call  LIKE 'IGHV3-23%'; |
| V + J + CDRH3 length + motif | SELECT COUNT(*) AS n  FROM read_parquet('data/Heavy/**/*.parquet')  WHERE v_call  LIKE 'IGHV3-30%' AND j_call LIKE 'IGHJ4%' AND cdr3_length = 15 AND cdr3_aa ~ '^AR'; |
| Paired IGHV×IG(K/L)V co-occurrence | SELECT v_call_heavy AS vh, v_call_light AS vl, COUNT(*) AS count  FROM read_parquet('data/Paired/**/*.parquet') GROUP BY v_call_heavy, v_call_light; |

DuckDB SQL equivalents of the AntibodySearchEngine examples in Supplementary Table 1, plus a paired IGHV×IG(K/L)V co-occurrence aggregation illustrating custom analyses beyond the standardized search mask. Queries read ABHunter Apache Parquet files via *read_parquet()*. Paths assume the local ABHunter data root. Sequence-file globs should exclude metadata.parquet.

## Supplementary Table 4: Storage footprint of OAS heavy, light, and paired datasets across file formats.

|  | **Dataset** | | | | **rel. storage optimization to CSV** | **rel. storage optimization to CSV.gz** |
| --- | --- | --- | --- | --- | --- | --- |
| **File type** | **Heavy [GB]** | **Light [GB]** | **Paired [GB]** | **Sum [GB]** |  |  |
| CSV | 7259.64 | 1210.41 | 5.61 | 8475.66 | 0 % | - 883.65 % |
| CSV.gz | 755.62 | 105.38 | 0.65 | 861.65 | 89.83 % | 0 % |
| Parquet (full db) | 675.15 | 92.68 | 0.44 | 768.27 | 90.94 % | 10.84 % |
| Parquet (optimized) | 91.69 | 14.26 | 0.06 | 106.01 | 98.75 % | 87.70 % |

Absolute sizes are reported in gigabytes for heavy-chain, light-chain, and paired partitions, together with their sum. Relative storage optimization is the percent reduction in total size versus uncompressed CSV or versus gzip-compressed CSV (CSV.gz). Positive values indicate a smaller footprint than the reference format.

## Supplementary Table 5: Progressive scalability versus legacy workflows

| **Sequences** | **Hits** | **ABHunter (s)** | **CSV (s)** | **CSV.gz (s)** | **ASE vs CSV** | **ASE vs. CSV.gz** |
| --- | --- | --- | --- | --- | --- | --- |
| 37,505 | 904 | 0.031 ± 0.001 | 1.53 ± 0.03 | 1.79 ± 0.02 | 49× | 57× |
| 69,535 | 1585 | 0.038 ± 0.002 | 2.73 ± 0.03 | 3.21 ± 0.03 | 71× | 84× |
| 95,367 | 2298 | 0.043 ± 0.003 | 3.55 ± 0.14 | 4.22 ± 0.14 | 82× | 97× |
| 140,923 | 3875 | 0.058 ± 0.006 | 5.12 ± 0.18 | 5.99 ± 0.09 | 88× | 103× |
| 217,443 | 5347 | 0.072 ± 0.005 | 7.91 ± 0.28 | 9.15 ± 0.20 | 110× | 127× |

Search latency for AntibodySearchEngine (ASE) (DuckDB/Parquet) compared with file-by-file Pandas on uncompressed CSV and gzipped CSV across progressive subset sizes of the unpaired heavy-chain corpus (query: IGHV3-30, full results). Values are replicate wall-clock times (mean ± SD, five measurement runs after two warmup runs). Hit counts were verified to be identical across backends. Speedup is ABHunter relative to each CSV workflow. Corresponds to Figure 4A.

## Supplementary Table 6: Progressive scalability on the unpaired heavy chain dataset

| **Sequences** | **Files** | **% of HC corpus** | **Stats only (s)** | **Full results, imit=100 (s)** | **Hits** |
| --- | --- | --- | --- | --- | --- |
| 106,019,019 | 4,357 | 6.7% | 0.579 ± 0.011 | 0.921 ± 0.038 | 5,217,583 |
| 211,832,230 | 4,547 | 13.4% | 0.656 ± 0.007 | 1.014 ± 0.056 | 11,045,381 |
| 317,611,627 | 4,646 | 20.0% | 0.738 ± 0.005 | 1.070 ± 0.024 | 17,017,255 |
| 423,386,602 | 4,712 | 26.7% | 0.788 ± 0.009 | 1.143 ± 0.013 | 23,469,006 |
| 530,252,742 | 4,762 | 33.5% | 0.879 ± 0.020 | 1.297 ± 0.038 | 28,952,965 |
| 740,864,359 | 4,837 | 46.7% | 1.024 ± 0.018 | 1.391 ± 0.024 | 40,065,275 |
| 951,613,757 | 4,900 | 60.0% | 1.189 ± 0.046 | 1.578 ± 0.024 | 50,994,757 |
| 1,164,453,733 | 4,955 | 73.5% | 1.491 ± 0.050 | 1.922 ± 0.082 | 62,574,546 |
| 1,481,301,998 | 5,029 | 93.5% | 1.761 ± 0.027 | 2.197 ± 0.060 | 76,386,938 |

Search latency at 9 progressive test points across subsets of the unpaired heavy-chain dataset. For each subset, times are reported for statistics-only summaries and full-result retrieval with limit=100 (query: IGHV3-30). Values are replicated wall-clock times (central tendency ± SD). Corresponds to Figure 4B.

## Supplementary Table 7: Progressive Pandas materialization

| **Sequences** | **Files** | **Time (s)** | **Peak resident set size (MB)** | **Hits** | **Status** |
| --- | --- | --- | --- | --- | --- |
| 500,445 | 2,772 | 6.40 | 223 | 32,106 | *ok* |
| 1,000,988 | 2,983 | 6.72 | 274 | 64,131 | *ok* |
| 2,000,776 | 3,182 | 8.01 | 399 | 131,779 | *ok* |
| 5,014,730 | 3,455 | 11.34 | 621 | 321,414 | *ok* |
| 10,006,057 | 3,665 | 15.47 | 961 | 604,916 | *ok* |
| 20,054,790 | 3,876 | 23.84 | 1,559 | 1,173,819 | *ok* |
| 40,083,804 | 4,085 | 41.03 | 2,993 | 2,328,063 | *ok* |
| 80,022,931 | 4,287 | 76.56 | 4,661 | 5,107,763 | *ok* |
| 100,064,658 | 4,342 | 92.87 | 5,701 | 6,372,198 | *ok* |
| 120,135,975 | 4,390 | 110.01 | 6,002 | - | *killed_memory_limit* |

Wall-clock time, peak process resident set size, retained IGHV3-30 hit count, and termination status for progressive Pandas materialization over unpaired heavy-chain Parquet subsets. Status *ok* indicates completion under the ceiling, *killed_memory_limit* indicates termination when the ceiling was exceeded.

## Supplementary Table 8: Search latency versus query complexity and retrieval mode

| **Complexity** | **Hits** | **Mode** | **Total (s)** | **Subject-stats SQL (s)** | **Results-fetch SQL (s)** |
| --- | --- | --- | --- | --- | --- |
| Simple | 79,771,390 | Stats only | 3.74 ± 0.29 | 3.73 ± 0.29 | 0.00 ± 0.00 |
| Simple | 79,771,390 | Full (limit=100) | 4.06 ± 0.21 | 3.66 ± 0.19 | 0.39 ± 0.02 |
| Simple | 79,771,390 | Full (limit=1M) | 13.55 ± 0.60 | 3.72 ± 0.11 | 8.27 ± 0.56 |
| Medium | 48,987,707 | Stats only | 5.72 ± 0.17 | 5.72 ± 0.17 | 0.00 ± 0.00 |
| Medium | 48,987,707 | Full (limit=100) | 5.97 ± 0.21 | 5.52 ± 0.21 | 0.44 ± 0.01 |
| Medium | 48,987,707 | Full (limit=1M) | 17.19 ± 0.29 | 5.86 ± 0.15 | 9.87 ± 0.28 |
| Complex | 6,937,562 | Stats only | 6.23 ± 0.20 | 6.22 ± 0.20 | 0.00 ± 0.00 |
| Complex | 6,937,562 | Full (limit=100) | 6.90 ± 0.14 | 6.08 ± 0.10 | 0.81 ± 0.05 |
| Complex | 6,937,562 | Full (limit=1M) | 38.60 ± 0.66 | 6.38 ± 0.07 | 30.79 ± 0.61 |
| Very Complex | 1,669,870 | Stats only | 20.82 ± 1.28 | 20.81 ± 1.28 | 0.00 ± 0.00 |
| Very Complex | 1,669,870 | Full (limit=100) | 23.76 ± 0.63 | 20.16 ± 0.50 | 3.59 ± 0.46 |
| Very Complex | 1,669,870 | Full (limit=1M) | 117.82 ± 1.10 | 20.58 ± 0.64 | 95.77 ± 0.98 |

Wall-clock times for four complexity levels (Simple: IGHV3-30, Medium: IGHV3-30 + IGHJ4, Complex: IGHV3-30 + IGHJ4 + CDRH3 length 15 aa, Very Complex: IGHV3-30 + IGHJ4 + CDRH3 length 15 aa + CDRH3 motif AR*). Measured in the same run under statistics-only mode and full-result retrieval with limit=100 and limit=1,000,000. Times are decomposed into result-fetch SQL and subject-stats (GROUP BY). Corresponds to Figure 4C.

## Supplementary Table 9: Cold versus warm cache behaviour and concurrency scaling

| **Query** | **Mode** | **Cold (s)** | **Warm (s)** | **Ratio** | **Hits** |
| --- | --- | --- | --- | --- | --- |
| Simple | Stats only | 8.16 ± 0.72 | 7.22 ± 0.38 | 1.13× | 79,771,390 |
| Simple | Full (limit=100) | 8.09 ± 0.74 | 7.73 ± 0.36 | 1.05× | 79,771,390 |
| Complex | Stats only | 13.13 ± 1.46 | 12.67 ± 0.40 | 1.04× | 6,937,562 |
| Complex | Full (limit=100) | 14.14 ± 0.06 | 14.25 ± 0.16 | 0.99× | 6,937,562 |
|  | | | | | |
| **Complexity** | **Mode** | **DuckDB threads** | **Mean time (s)** | **SD (s)** | **Matching hits** |
| Simple | Statistics only | 1 | 18.61 | 0.99 | 79,771,390 |
| Simple | Statistics only | 2 | 7.84 | 0.58 | 79,771,390 |
| Simple | Statistics only | 4 | 3.58 | 0.10 | 79,771,390 |
| Simple | Full results (limit=100) | 1 | 17.44 | 0.58 | 79,771,390 |
| Simple | Full results (limit=100) | 2 | 7.67 | 0.23 | 79,771,390 |
| Simple | Full results (limit=100) | 4 | 3.99 | 0.20 | 79,771,390 |
| Complex | Statistics only | 1 | 29.07 | 0.36 | 6,937,562 |
| Complex | Statistics only | 2 | 13.75 | 0.02 | 6,937,562 |
| Complex | Statistics only | 4 | 6.97 | 0.04 | 6,937,562 |
| Complex | Full results (limit=100) | 1 | 30.70 | 0.41 | 6,937,562 |
| Complex | Full results (limit=100) | 2 | 15.30 | 0.52 | 6,937,562 |
| Complex | Full results (limit=100) | 4 | 8.04 | 0.08 | 6,937,562 |
|  | | | | | |
| **Complexity** | **Mode** | **Concurrent processes** | **Mean time (s)** | **SD (s)** | **Throughput (qps)** |
| Simple | Statistics only | 1 | 7.74 | 0.19 | 0.128 |
| Simple | Statistics only | 2 | 9.10 | 0.82 | 0.218 |
| Simple | Full results (limit=100) | 1 | 7.80 | 0.33 | 0.127 |
| Simple | Full results (limit=100) | 2 | 9.08 | 0.46 | 0.218 |
| Complex | Statistics only | 1 | 13.92 | 0.29 | 0.072 |
| Complex | Statistics only | 2 | 15.56 | 0.84 | 0.128 |
| Complex | Full results (limit=100) | 1 | 13.10 | 0.40 | 0.076 |
| Complex | Full results (limit=100) | 2 | 15.36 | 0.30 | 0.129 |

Cold-start versus warm-cache latency, DuckDB thread scaling (1, 2, and 4 threads), and concurrent client throughput (1–2 independent search processes) for ABHunter queries on the full unpaired heavy-chain corpus. Simple = V gene only (IGHV3-30); Complex = V+J+CDRH3 length. Full-results mode retrieves limit=100 filtered records. Values are mean ± SD over repeated runs.

## Supplementary Table 10: Correlation of intra-chain V(D)J gene usage between paired and unpaired dataset.

| **Compared between Paired:Unpaired** | **n** | **Pearson r** | **Pearson *p*-value** | **Spearman ρ** | **Spearman *p*-value** |
| --- | --- | --- | --- | --- | --- |
| IGHV (Family) | 8 | 0.9851 | 8.11×10^-6^ | 0.9762 | 3.31×10^-5^ |
| IGHD (Family) | 7 | 0.9890 | 2.43×10^-5^ | 0.9286 | 2.52×10^-3^ |
| IGHJ (Family) | 6 | 0.9935 | 6.29×10^-5^ | 0.9429 | 4.80×10^-3^ |
| IG(K/L)V (Family) | 18 | 0.9071 | 2.07×10^-7^ | 0.9582 | 4.12×10^-10^ |
| IG/K/L)J (Family) | 12 | 0.9136 | 3.28×10^-5^ | 0.9579 | 9.71×10^-7^ |
|  | | | | | |
| **Compared between Paired:Unpaired** | **n** | **Pearson r** | **Pearson p-value** | **Spearman ρ** | **Spearman *p*-value** |
| IGHV (Family-Gene) | 64 | 0.9185 | 1.15×10^-26^ | 0.9344 | 1.74×10^-29^ |
| IGHD (Family-Gene) | 26 | 0.9301 | 6.30×10^-12^ | 0.8983 | 4.77×10^-10^ |
| IGHJ (Family-Gene) | 6 | 0.9935 | 6.29×10^-5^ | 0.9429 | 4.80×10^-3^ |
| IG(K/L)V (Family-Gene) | 62 | 0.8789 | 5.95×10^-21^ | 0.9208 | 3.27×10^-26^ |
| IG(K/L)J (Family) | 12 | 0.9136 | 7.96×10^-4^ | 0.9579 | 1.12×10^-4^ |

Values represent Pearson (r) and Spearman (ρ) correlation coefficients comparing relative family frequencies and rank order, respectively, between the paired and unpaired subset. Corresponding *p*-values are provided to indicate statistical significance (see also Supplementary Figure 2).

## Supplementary Table 11: Similarity of intra-chain co-pairing frequencies between paired and unpaired datasets.

| **Feature** | **Comparing** | **Cosine Similarity** | **Jensen-Shannon Divergence** |
| --- | --- | --- | --- |
| IGHV×IGHD | paired vs. unpaired | 0.988 | 0.095 |
| IGHV×IGHJ | paired vs. unpaired | 0.988 | 0.104 |
| IG(K/L)V×IG(K/L)J | paired vs. unpaired | 0.882 | 0.200 |
| IGHD×IGHJ | paired vs. unpaired | 0.992 | 0.071 |

Values compare the distribution of intra-chain gene pairing frequencies across datasets. Cosine similarity measures directional agreement between profiles (1 = identical), whereas Jensen-Shannon distance quantifies distributional divergence (0 = identical). Higher similarity and lower distance values indicate agreement between unpaired and natively paired intra-chain usage patterns. Intra-chain co-pairings are visualized in Supplementary Figure 3.

## Supplementary Table 12: Statistical parameters for the heavy and light chain pairing model for both V and J.

| **Feature** | **IGHV×IG(K/L)V** | **IGHJ×IG(K/L)J** |
| --- | --- | --- |
| Comparing | paired vs. random based on gene abundance | paired vs. random based on gene abundance |
| χ^2^ | 9606.2 | 1840.1 |
| Degrees of freedom | 90 | 45 |
| Cells with expected counts <5 | 2 (1.79%) | 8.33% |
| Minimum expected count | 1.17 | 0.17 |
| Cells with expected count <1 | 0 | 0 |
| *p*-value | *p* < 10^-308^ | *p* < 10^-308^ |
| Cramér’s V effect size | 0.053 | 0.025 |
| Total variation distance | 0.044 | 0.021 |

Pearson χ^2^ tests of independence for the heavy–light pairing models (IGHV×IGLV/IGKV and IGHJ×IGLJ/IGKJ), comparing observed family co-occurrence counts with counts expected under random pairing. Exact SciPy p values are reported with Cramér’s V and Total Variation Distance. Cochran’s rule (cells with expected count <5 or <1, and the minimum expected count) assess test validity. Where *scipy.stats.chi2_contingency* returns 0.0 due to floating-point underflow, *p* is reported as *p* < 10^-308^.

## Supplementary Table 13: Leave-one-donor-out Spearman correlation for the IGHV-IG(K/L) pairing-correction matrix R.

| **Left-out donor** | **Sequences left out** | **Spearman ρ vs full R (all cells)** | **Spearman ρ vs full R (informative)** |
| --- | --- | --- | --- |
| Donor-3 | 350209 (61.64%) | 0.841 | 0.847 |
| Donor-2 | 130261 (22.93%) | 0.862 | 0.880 |
| Donor-1 | 74793 (12.16%) | 0.985 | 0.986 |
| 417c | 11091 (1.95%) | 0.995 | 0.995 |
| 390c | 1012 (0.18%) | ≈1 | ≈1 |
| Healthy-1 | 771 (0.14%) | ≈1 | ≈1 |
|  | | mean=0.947  SD=0.075 | mean=0.951  SD=0.069 |
|  | | | |
| **Left-out donor** | **Sequences left out** | **Spearman ρ vs held-out R (all cells)** | **Spearman ρ vs held-out R (informative)** |
| Donor-3 | 350209 (61.64%) | 0.547 | 0.582 |
| Donor-2 | 130261 (22.93%) | 0.549 | 0.543 |
| Donor-1 | 74793 (12.16%) | 0.539 | 0.573 |
| 417c | 11091 (1.95%) | 0.236 | 0.177 |
| 390c | 1012 (0.18%) | 0.074 | 0.202 |
| Healthy-1 | 771 (0.14%) | 0.064 | 0.104 |
|  | | mean=0.335  SD=0.238 | mean=0.363  SD=0.224 |

The upper block reports Spearman ρ between each five-donor matrix and the full six-donor matrix R. The lower block reports Spearman ρ between each five-donor matrix and the corresponding held-out donor matrix. “All cells” include all 112 active family combinations. “Informative” is restricted to cells with raw count n>0 in the full six-donor reference (upper block) or in the held-out donor (lower block).

## Supplementary Table 14: Leave-one-donor-out Spearman correlation for the IGHJ-IG(K/L)J pairing-correction matrix R.

| **Left-out donor** | **Sequences left out** | **Spearman ρ vs full R  (all cells)** | **Spearman ρ vs full R  (informative)** |
| --- | --- | --- | --- |
| Donor-3 | 350209 (61.64%) | 0.696 | 0.713 |
| Donor-2 | 130261 (22.93%) | 0.803 | 0.803 |
| Donor-1 | 74793 (12.16%) | 0.864 | 0.870 |
| 417c | 11091 (1.95%) | 0.999 | 0.998 |
| 390c | 1012 (0.18%) | ≈1 | ≈1 |
| Healthy-1 | 771 (0.14%) | ≈1 | ≈1 |
|  | | mean=0.894  SD0.128 | mean=0.897  SD0.122 |
|  | | | |
| **Left-out donor** | **Sequences left out** | **Spearman ρ vs held-out R  (all cells)** | **Spearman ρ vs held-out R (informative)** |
| Donor-3 | 350209 (61.64%) | 0.306 | 0.334 |
| Donor-2 | 130261 (22.93%) | 0.287 | 0.276 |
| Donor-1 | 74793 (12.16%) | 0.349 | 0.433 |
| 417c | 11091 (1.95%) | 0.472 | 0.554 |
| 390c | 1012 (0.18%) | -0.022 | -0.001 |
| Healthy-1 | 771 (0.14%) | 0.184 | 0.206 |
|  | | mean=0.262  SD0.168 | mean=0.300  SD0.191 |

The upper block reports Spearman ρ between each five-donor matrix and the full six-donor matrix R. The lower block reports Spearman ρ between each five-donor matrix and the corresponding held-out donor matrix. “All cells” include all 60 active family combinations. “Informative” is restricted to cells with raw count n>0 in the full six-donor reference (upper block) or in the held-out donor (lower block).

## Supplementary Table 15: Search parameters for determining bnAb precursor frequencies.

| **bnAb-class** | **Virus** | **Epitope** | **Search features** |
| --- | --- | --- | --- |
| PG9 (A) (13) | HIV-1 | Apex region | IGHV: 3  IGHJ: 6  CDRH3 length: 30  CDRH3 motif: .{15}YDF.{12} |
| 10E8 (14) | HIV-1 | MPER helical peptide | Shorter CDRH3  IGHV: 3-15, 3-49, 3-72, 3-73  H3 length: 21-22  H3 motif: .{6}Y.FW* |
|  |  |  | Longer CDRH3  IGHV: 3-15, 3-49, 3-72, 3-73  H3 length: 23-24  .{7}Y.FW* |
| PG9 (B) (15) | HIV-1 | Apex region | IGHD3-3  H3 length: >=29  H3 motif: .{15}*[FI]W[ST].{8}* |
| PCT64 (A) (13) | HIV-1 | V2-Apex | IGHV: 3  IGHJ: 6  H3 length: 25  H3 motif: .{6}[YRKG][DSG]FWS.{14} |
| Apex (15) | HIV-1 | Apex region | H3 length: >=24  H3 motif: .{7}*[DE][DE]Y.{10}* |
| BG18 (Type I) (16) | HIV-1 | V3-glycan/N332 supersite | IGHD: 3-3  IGHJ: 6  H3 lenght: >=22  H3 motif: .{4-6}ITIFG[LV]VI[IT]*  Mismatches: 3 |
| PCT64 (B) (15) | HIV-1 | Apex region | IGHD: 3-3  H3 length: >=24  H3 motif: .{6}*[FI]W[ST]*.{12} |
| 76E1 (17)  PDB ID: 7X9E | Coronavirus | Fusion Peptide | VDJ search:  IGHV: 3-43  IGHD: 2-15  IGHJ: 3 |
|  |  |  | V+H3 search:  IGHV: 3-43  H3 motif: *IV.A* |
| 3D1 (18) | Coronavirus | HR1 domain | VDJ search:  IGHV: 7-4-1  IGHD: 6-19  IGHJ: 4 |
|  |  |  | V+H3 search:  IGHV: 7-4-1  H3 motif: *PV.W* |
| ZCB11 (19)  PDB ID: 7XH8 | Coronavirus | RBD in up conformation | VDJ search:  IGHV: 1-58  IGHD: 2-2  IGHJ: 3 |
|  |  |  | V+H3 search:  IGHV: 1-58  H3 motif: *RTI* |
| ADI-42898 (20)  PDB ID: 7QQB | Orthohantavirus | Gc fusion loop & main chain of Gn | VDJ search:  IGHV: 3-30-3  IGHD: 2-21  IGHJ: 6 |
|  |  |  | V+H3 search:  IGHV: 3-30-3  H3 motif: *RY.G* |
|  |  |  | V+H3p search:  IGHV: 3-30-3  H3 motif: .{5}RY.G* |

Here we show the exact search parameters used to obtain inferred bnAb precursor frequencies.

## Supplementary Table 16: Comparison of selected reference bnAb frequencies from the literature.

| **bnAb** | **Selected reference values (per million)** | **Matching ABHunter value** | **Zero hit donors** | **Absolute log_10_ difference** | **Fold Difference** |
| --- | --- | --- | --- | --- | --- |
| PG9 (A) (13) | 0.23 (median) | 0.38 (median) | excluded | 0.22 | 1.67x |
| 10E8 (14) | 14.7 (geomean) | 25.20 (geomean) | excluded | 0.23 | 1.71x |
| PG9 (B) (15) | 31.6 (median) | 27.65 (median) | excluded | 0.06 | 1.14x |
| PCT64 (A) (13) | 20.614 (median) | 26.21 (median) | excluded | 0.10 | 1.27x |
| Apex (15) | 63.12 (median) | 53.86 (median) | excluded | 0.07 | 1.17x |
| BG18 (Type I) (16) | 361 (median) | 300.64 (median) | excluded | 0.08 | 1.20x |
| PCT64 B (15) | 1288 (median) | 1345.90 (median) | excluded | 0.02 | 1.04x |
| Median: | | |  | 0.08 | 1.20x |

Here we show a comparison of selected Reference bnAb from the literature with Results obtained from ABHunter. Statistical metrics are individually matched to those reported in the corresponding literature.

## Supplementary Table 17: Comparison of reported and ABHunter-derived HIV-1 bnAb precursor frequencies.

| **bnAb** | **Reference values** | **ABHunter results** |
| --- | --- | --- |
| PG9 (A) (13) | median (all): 0.135  geomean (for >1 hit): 0.220  median (for >1 hit): 0.23 | total hits: 129  donor count: 9  mean: 1.33/M  median: 0.38/M  geomean: 0.30/M |
| 10E8 (14) | geomean: 14.7 | total hits: 16,820  donor count: 79  mean: 45.90/M  median: 26.55/M  geomean: 25.2/M |
| PG9 (B) (15) | median (no 0-hit donors, but >0 for rhesus macaque): 31.6 | total hits: 22,036  donor count: 70  mean: 67.25/M  median: 27.65/M  geomean: 27.36 |
| PCT64 (A) (13) | median (all): 20.614  geomean (for >1 hit, but here all as no 0-hit donors): 25.110 | total hits: 24,594  donor count: 85  mean; 41.87/M  median: 26.21/M  geomean: 27.32/M |
| Apex (15) | median (all, no 0-hit donors, but >0 for rhesus macaque): 63.12 | total hits: 52,281  donor count: 94  mean: 69.12/M  median: 53.86/M  geomean: 46.10/M |
| BG18 (Type I) (16) | median (all, no 0-hit donors, but >0 for rhesus macaque): 361 | total hits: 254,970  donor count: 116  mean: 430.09/M  median: 300.64/M  geomean: 274.60/JM |
| PCT64 B (15) | median (all, no 0-hit donors, but >0 for rhesus macaque): 1288 | total hits: 1,417,396  donor count: 121  mean: 1594.62/M  median: 1345.9/M  geomean: 1032.30/M |

For bnAbs with multiple reported reference values, the underscored value was selected for visualization in Figure 6A, see also Supplementary 9. Donor count represents the number of donors exceeding the total sequence count threshold and therefore used for calculating mean and median frequencies per million sequences. Zero-hit represents the number of plotted donors with 0 hit for the respective searched features. All searches were performed against the full heavy chain IgM dataset.

## Supplementary Table 18: Statistical uncertainty intervals for novel precursor target evaluations

| **bnAb (search)** | **Donors above threshold** | **Positiv Donors** | **Observed Donor Prevalence [%] [95% Wilson CI]** | **Median Precursor Frequency [HPM] [95% Exact Median CI]** |
| --- | --- | --- | --- | --- |
| 76E1 (VDJ) | 38 | 28 | 73.7 [58.0 - 85.0] | 5.74 [3.02 - 8.17] |
| 76E1 (V+H3) | 44 | 30 | 68.2 [53.4 - 80.0] | 9.15 [3.07 - 12.86] |
| 3D1 (VDJ) | 16 | 6 | 37.5 [18.5 - 61.4] | 4.73 [2.38 - 6.7] |
| 3D1 (V+H3) | 121 | 64 | 52.9 [44.0 - 61.6] | 131.81 [16.48 - 241.84] |
| ZCB11 (VDJ) | 45 | 37 | 82.2 [68.7 - 90.7] | 19.35 [12.19 - 21.55] |
| ZCB11 (V+H3) | 14 | 13 | 92.9 [68.5 - 98.7] | 0.63 [0.43 - 1.32] |
| ADI-42898 (VDJ) | 108 | 58 | 53.7 [44.3 - 62.8] | 86.93 [64.99 - 106.52] |
| ADI-42898 (V+H3) | 111 | 62 | 55.9 [46.6 - 64.7] | 97.73 [62.94 - 124.89] |
| ADI-42898 (V+H3p) | 42 | 25 | 59.5 [44.5 - 73.0] | 11.97 [4.07 - 16.45] |

Observed donor prevalence represents the percentage of positive donors within the cohort of depth-qualified donors with 95% confidence intervals estimated using the Wilson score method. Median precursor frequencies (hits per million, HPM) and corresponding 95% confidence intervals are calculated from positive donors (>0 hits) using a non-parametric exact binomial method based on sorted order statistics. Zero-hit donors were excluded from both the box plots (Figure 6A) and median calculations.

## Supplementary Table 19: Sensitivity analysis of the sequence depth filter on precursor prevalence and frequency metrics.

| **bnAb**  **(search)** | **With sequencing threshold** | | | | **Without sequencing threshold** | | | |
| --- | --- | --- | --- | --- | --- | --- | --- | --- |
|  | Total Donors | Zero-hit donors | Prevalence (%) | Median HPM | Total Donors | Zero-hit donors | Prevalence (%) | Median HPM |
| 76E1 (VDJ) | 38 | 10 | 73.68 | 5.74 | 154 | 98 | 36.36 | 15.3 |
| 76E1 (V+H3) | 44 | 14 | 68.18 | 9.15 | 154 | 101 | 34.42 | 14.58 |
| 3D1 (VDJ) | 16 | 10 | 37.5 | 4.73 | 154 | 140 | 9.09 | 5.86 |
| 3D1 (V+H3) | 121 | 57 | 52.89 | 131.81 | 154 | 88 | 42.86 | 140.57 |
| ZCB11 (VDJ) | 45 | 8 | 82.22 | 19.35 | 154 | 81 | 47.4 | 21.55 |
| ZCB11 (V+H3) | 14 | 1 | 92.86 | 0.63 | 154 | 128 | 16.88 | 1.77 |
| ADI-42898 (VDJ) | 108 | 50 | 53.7 | 86.93 | 154 | 90 | 41.56 | 81.1 |
| ADI-42898 (V+H3) | 111 | 49 | 55.86 | 97.73 | 154 | 88 | 42.86 | 97.73 |
| ADI-42898 (V+H3p) | 42 | 17 | 59.52 | 11.97 | 154 | 115 | 25.32 | 13.53 |

Listed are the donor counts, zero hit donor counts, medians (calculated exclusively from positive responders, excluding zero hit donors), and prevalence values calculated both with and without the sequence depth threshold. These metrics are shown for all Coronavirus and Orthohantavirus targets across all evaluated search settings to demonstrate classification robustness, with the corresponding donor distributions plotted in Supplementary Figure 7.

## Supplementary Table 20: Precursor frequencies for Coronavirus and Orthohantavirus bnAbs.

| **bnAb** | **VDJ search results** | **V+H3(p) search results** |
| --- | --- | --- |
| 76E1 (17) | total hits: 10,598  threshold: ≥869,582  donor count: 18 (1 zero-hit)  mean: 7.542/M  median: 3.651/M | total hits: 12,115  threshold: ≥760,696  donor count: 18 (1 zero-hit)  mean: 11.35/M  median: 7.852/M |
| 3D1 (18) | total hits: 1,295  threshold: ≥7,116,464  donor count: 14 (10 zero-hits)  mean: 0.8911/M  median: 0/M | total hits: 198,165  threshold: ≥46,506  donor count: 102 (44 zero-hits)  mean: 146.6/M  median: 2.547/M |
| ZCB11 (19) | total hits: 12,965  threshold: ≥710,824  donor count: 19 (0 zero-hits)  mean: 24.11  median: 16.23 | total hits: 739  threshold: ≥12,470,664  donor count: 13 (1 zero-hits)  mean: 0.6925/M  median 0.5015/M |
| ADI-42898 (20) | total hits: 69,197  threshold: ≥133,183  donor count: 65 (27 zero-hits)  mean: 63.93/M  median: 21.51/M | V+H3 search:  total hits: 87,986  threshold: ≥104,742  donor count: 70 (25 zero-hits)  mean: 80.93/M  median: 35.62/M  V+H3p search:  total hits: 11,602  threshold: ≥794,331  donor count: 18 (3 zero-hits)  mean: 10.15/M  median: 4.21/M |

Donor count represents the number of donors exceeding the total sequence count threshold and therefore used for calculating mean and median frequencies per million sequences. Zero-hit represents the number of plotted donors with 0 hit for the respective searched features. All searches were performed against the full heavy chain IgM dataset.

## Supplementary Table 21: Genetic features of the clinical BG18 isolate and motif search parameter.

| **Antibody** | **Features clinical isolate** | **Searched feature** |
| --- | --- | --- |
| BG18 (16) | IGHV: 4-4  IGHD: 3-3  IGHJ: 6  IGLV: L3-25  IGLJ: L3 | H3 motif: .{6-8}FGV….E* |

Listed are the genetic features of the clinical BG18 isolate and the searched motif. The search was performed on the full unpaired chain dataset (heavy + light).

## Supplementary Table 22: Retained and discarded columns during optimization.

| **Retained Features** | |
| --- | --- |
| **Unpaired (Heavy and Light)** | **Paired** |
| v_call | v_call_heavy and _light |
| d_call (heavy only) | d_call_heavy |
| j_call | j_call_heavy and _light |
| sequence_alignment_aa | sequence_alignment_aa_heavy and _light |
| v_sequence_alignment_aa | v_sequence_alignment_aa_heavy and _light |
| d_sequence_alignment_aa (heavy only) | d_sequence_alignment_aa_heavy |
| j_sequence_alignment_aa | j_sequence_alignment_aa_heavy and _light |
| v_germline_alignment_aa | v_germline_alignment_aa_heavy and _light |
| d_germline_alignment_aa (heavy only) | d_germline_alignment_aa_heavy |
| j_germline_alignment_aa | j_germline_alignment_aa_heavy and _light |
| cdr1_aa | cdr1_aa_heavy and _light |
| cdr2_aa | cdr2_aa_heavy and _light |
| cdr3_aa | cdr3_aa_heavy and _light |
|  |  |
| **Discarded Features** | |
| **Unpaired (Heavy and Light)** | **Paired** |
| sequence_id | sequence_id_heavy and _light |
| sequence | sequence_heavy and _light |
| d_call (light only) | d_call_light |
| locus | locus_heavy and _light |
| stop_codon | stop_codon_heavy and _light |
| vj_in_frame | vj_in_frame_heavy and _light |
| v_frameshift | v_frameshift_heavy and _light |
| productive | productive_heavy and _light |
| rev_comp | rev_comp_heavy and _light |
| complete_vdj | complete_vdj_heavy and _light |
| sequence_alignment | sequence_alignment_heavy and _light |
| germline_alignment | germline_alignment_heavy and _light |
| germline_alignment_aa | germline_alignment_aa_heavy and _light |
| v_alignment_start | v_alignment_start_heavy and _light |
| v_alignment_end | v_alignment_end_heavy and _light |
| d_alignment_start | d_alignment_start_heavy and _light |
| d_alignment_end | d_alignment_end_heavy and _light |
| j_alignment_start | j_alignment_start_heavy and _light |
| j_alignment_end | j_alignment_end_heavy and _light |
| v_sequence_alignment | v_sequence_alignment_heavy and _light |
| v_germline_alignment | v_germline_alignment_heavy and _light |
| v_germline_alignment_aa | v_germline_alignment_aa_heavy and _light |
| d_sequence_alignment | d_sequence_alignment_heavy and _light |
| d_germline_alignment | d_germline_alignment_heavy and _light |
| j_sequence_alignment | j_sequence_alignment_heavy and _light |
| j_sequence_alignment_aa | j_sequence_alignment_aa_heavy and _light |
| j_germline_alignment | j_germline_alignment_heavy and _light |
| j_germline_alignment_aa | j_germline_alignment_aa_heavy and _light |
| fwr1 | fwr1_heavy and _light |
| fwr1_aa | fwr1_aa_heavy and _light |
| cdr1 | cdr1_heavy and _light |
| fwr2 | fwr2_heavy and _light |
| fwr2_aa | fwr2_aa_heavy and _light |
| cdr2 | cdr2_heavy and _light |
| fwr3 | fwr3_heavy and _light |
| fwr3_aa | fwr3_aa_heavy and _light |
| fwr4 | fwr4_heavy and _light |
| fwr4_aa | fwr4_aa_heavy and _light |
| cdr3 | cdr3_heavy and _light |
| junction | junction_heavy and _light |
| junction_length | junction_length_heavy and _light |
| junction_aa | junction_aa_heavy and _light |
| junction_aa_length | junction_aa_length_heavy and _light |
| v_score | v_score_heavy and _light |
| d_score | d_score_heavy and _light |
| j_score | j_score_heavy and _light |
| v_cigar | v_cigar_heavy and _light |
| d_cigar | d_cigar_heavy and _light |
| j_cigar | j_cigar_heavy and _light |
| v_support | v_support_heavy and _light |
| d_support | d_support_heavy and _light |
| j_support | j_support_heavy and _light |
| v_identity | v_identity_heavy and _light |
| d_identity | d_identity_heavy and _light |
| j_identity | j_identity_heavy and _light |
| v_sequence_start | v_sequence_start_heavy and _light |
| v_sequence_end | v_sequence_end_heavy and _light |
| v_germline_start | v_germline_start_heavy and _light |
| v_germline_end | v_germline_end_heavy and _light |
| d_sequence_start | d_sequence_start_heavy and _light |
| d_sequence_end | d_sequence_end_heavy and _light |
| d_germline_start | d_germline_start_heavy and _light |
| d_germline_end | d_germline_end_heavy and _light |
| j_sequence_start | j_sequence_start_heavy and _light |
| j_sequence_end | j_sequence_end_heavy and _light |
| j_germline_start | j_germline_start_heavy and _light |
| j_germline_end | j_germline_end_heavy and _light |
| fwr1_start | fwr1_start_heavy and _light |
| fwr1_end | fwr1_end_heavy and _light |
| cdr1_start | cdr1_start_heavy and _light |
| cdr1_end | cdr1_end_heavy and _light |
| fwr2_start | fwr2_start_heavy and _light |
| fwr2_end | fwr2_end_heavy and _light |
| cdr2_start | cdr2_start_heavy and _light |
| cdr2_end | cdr2_end_heavy and _light |
| fwr3_start | fwr3_start_heavy and _light |
| fwr3_end | fwr3_end_heavy and _light |
| fwr4_start | fwr4_start_heavy and _light |
| fwr4_end | fwr4_end_heavy and _light |
| cdr3_start | cdr3_start_heavy and _light |
| cdr3_end | cdr3_end_heavy and _light |
| np1 | np1_heavy and _light |
| np1_length | np1_length_heavy and _light |
| np2 | np2_heavy and _light |
| np2_length | np2_length_heavy and _light |
| c_region | c_region_heavy and _light |
| Redundancy | Redundancy_heavy and _light |
| ANARCI_numbering | ANARCI_numbering_heavy and _light |
| ANARCI_status | ANARCI_status_heavy and _light |

While optimizing the dataset for memory footprint, non-essential columns were dropped. The upper section shows the remaining columns in the optimized dataset for paired and unpaired partitions. The lower part shows the discarded columns respectively.

## Supplementary Table 23: Retained and discarded metadata during optimization.

| Retained Metadata | Discarded Metadata |
| --- | --- |
| Species | Run |
| Disease | Link |
| Subject | Author |
| Vaccine | BSource |
| Isotype | Btype |
| Total sequences (only unpaired) | Longitudinal |
|  | Age |
|  | Chain |
|  | Unique Sequences |

In addition to the reduced subset of maintained columns in the optimized dataset, metadata fields that are not used in any of the search dimensions are discarded. This table shows retained and discarded metadata features.

## Supplementary Table 24: Sequence column definitions.

| **Dataset** | **Sequence Column** | **Source** | **Description** |
| --- | --- | --- | --- |
| Unpaired | v_call, d_call (heavy only), j_call | OAS | V/D/J gene assignments |
| Unpaired | sequence_alignment_aa | OAS | Full amino-acid alignment |
| Unpaired | v_sequence_alignment_aa, d_sequence_alignment_aa (heavy only), j_sequence_alignment_aa | OAS | V/D/J segment alignments (AA) |
| Unpaired | v_germline_alignment_aa, d_germline_alignment_aa (heavy only), j_germline_alignment_aa | OAS | Germline reference alignments (AA) |
| Unpaired | cdr1_aa, cdr2_aa, cdr3_aa | OAS | CDR amino-acid sequences |
| Unpaired | cdr1_length, cdr2_length, cdr3_length | Derived cdr1/2/3_aa | Length of each CDR (AA) |
| Paired | All unpaired columns + _heavy and _light suffix | OAS/derived | see Unpaired |

This table shows the data origin for all defined sequence columns in the dataset.

## Supplementary Table 25: Metadata column definitions

| **Metadata Column** | **Description** |
| --- | --- |
| file_path (str) | Relative path to sequence Parquet file (e.g. Heavy/IGHG/study.parquet) |
| chain (str) | Heavy, Light, or Paired |
| file_source (str) | Original OAS CSV filename |
| species (str) | Species from OAS metadata |
| subject (str) | Subject identifier |
| author (str) | Study author (from OAS JSON index) |
| disease (str) | Disease status |
| vaccine (str) | Vaccination status |
| isotype (str) | Immunoglobulin isotype |
| total_sequences (int) | Number of sequences in the file |

The same metadata fields are also stored as file-level Parquet metadata in each sequence file. All sample descriptors were stored as strings except *total_sequences* (integer).

## Supplementary Table 26: DuckDB configuration

| **Setting** | **Description** | **Value** |
| --- | --- | --- |
| memory_limit | maximum amount of RAM DuckDB is allowed to use, excess working memory is spilled to disk (temp_directory) | 40 GB  (ABHUNTER_DUCKDB_MEMORY_LIMIT) |
| threads | number of CPU threads DuckDB may use to run a query in parallel | 4 (ABHUNTER_DUCKDB_THREADS) |
| allocator_background_threads | if DuckDB uses background threads for memory management/cleanup | true |
| temp_directory | folder where DuckDB writes temporary files when memory_limit is exceeded(for large queries or exports) | /tmp/duckdb_tmp |
| Indexing | No CREATE INDEX sans | None (No CREATE INDEX) |
| File pruning | Searches limited to Heavy/Light/Paired file subset | Chain-specific views |
| Predicate pushdown | Filters pushed to Parquet readers where possible | Enabled |

This table shows the Default options when deploying ABHunter locally by invoking ‘*streamlit run app*’.

## Supplementary Table 27: Download handling for search results and plots under local install versus Docker/webserver deployment

| **Condition** | **Local install** | **Docker/Webserver** |
| --- | --- | --- |
| Write directory | ./downloads (or ABHUNTER_DOWNLOAD_DIR) | /app/downloads (shared volume) |
| File ≤ 1 GB | st.download_button (loaded into memory) | st.download_button (loaded into memory) |
| File > 1 GB | Saved to disk. UI shows absolute path (browser download disabled) | Saved to disk. nginx link at /downloads/{filename} |
| nginx | Not used | Separate container |
| nginx behavior | – | Content-Disposition: attachment. Post-download callback to cleanup service |
| Cleanup | Manual | cleanup_downloads.py every 300 s after nginx callback |
| Plots ≤ 1 GB | st.download_button (loaded into memory) | st.download_button (loaded into memory) |
| Plots > 1 GB | st.download_button (in memory) | Saved to disk. nginx link at /downloads/{filename} |

For each deployment mode, the table summarizes where files are written, how downloads are served for result files and plots below or above the 1 GB threshold, and whether nginx and automated cleanup are used. Local installs expose large result files by absolute path with browser download disabled. The Docker/webserver deployment serves files above 1 GB through nginx with *Content-Disposition: attachment* and removes them after download via a cleanup callback polled every 300 seconds.

## Supplementary Table 28: Accepted CDR length search syntax

| **Syntax** | **Meaning** | **Example** |
| --- | --- | --- |
| N | Exact length | 9 |
| N-M | Inclusive range | 18-22 |
| >N | Strictly greater than | >2 |
| >=N | Greater than or equal | >=29 |
| <N | Strictly less than | <5 |
| <=N | Less than or equal | <=10 |

CDR length filters accept an exact integer, an inclusive range, or a comparison operator. The same syntax applies to all heavy- and light-chain CDR length fields (CDRH1-3 and CDRL1-3).

## Supplementary Table 29: Mapping of ABHunter sequence-pattern input to RE2 regular expressions

| **Example input** | **Description** | **RE2 pattern** | **Note** |
| --- | --- | --- | --- |
| ABHNTER | Literal amino acid (ACDEFGHIKLMNPQRSTVWY) | Exact residue | Case-insensitive input, stored as uppercase |
| . | Any single residue | . | One amino-acid position |
| * | Variable-length gap | .* | Matches zero or more residues |
| [AR] | Allowed alternatives at one position | [AR] | Only standard amino-acid letters allowed inside brackets |
| {5} | Exactly 5 residues | .{5} | Applied to wildcard positions |
| .{5}* | At least 5 residues | .{5}* | Applied to wildcard positions |
| {2-4} | 2-4 residues | .{2,4} | Range quantifier |
| Similarity mode off | Exact residue only | Literal match | No group expansion |
| Similarity mode on | Chemically similar residues | Position-specific character classes | e.g. Y → [FWY], D → [DE] |
| Any other character | Literal text/escaped | Escaped literal | e.g., +, (, ) not treated as regex operators and are not supported |

User-facing pattern tokens are translated to RE2 syntax before search. Literal amino acids are matched exactly and stored uppercase. Wildcard (.), gap (*), alternative ([…]), and length ({n}, {n-m}) tokens expand as listed. With similarity mode enabled, each literal residue is replaced by its physicochemical character class (Supplementary Table 21). Characters outside this grammar are not interpreted as regex operators and are not supported.

## Supplementary Table 30: Amino-acid property classes used for conservative substitution

| **Property class** | **Residues** |
| --- | --- |
| Aliphatic | A, I, L, V |
| Acidic | D, E |
| Basic | K, R |
| Aromatic | F, W, Y |
| Hydroxyl | S, T |
| Amide | N, Q |
| Unique (no substitution) | C, G, H, M, P |

Residues are grouped by shared physicochemical properties. Unique residues (C, G, H, M, P) are not substituted by other amino acids.

## Supplementary Table 31: Absolute counts of IGHV:IG(K/L)V gene family combinations.

|  | KV1 | KV2 | KV3 | KV4 | KV5 | KV6 | KV7 | LV1 | LV2 | LV3 | LV4 | LV5 | LV6 | LV7 | LV8 | LV9 | LV10 | LV11 |
| --- | --- | --- | --- | --- | --- | --- | --- | --- | --- | --- | --- | --- | --- | --- | --- | --- | --- | --- |
| HV1 | 23806 | 6821 | 22600 | 7664 | 17 | 257 | 0 | 10298 | 11496 | 10566 | 744 | 300 | 680 | 1254 | 458 | 115 | 215 | 0 |
| HV2 | 5578 | 1235 | 3652 | 1063 | 11 | 69 | 0 | 3161 | 2508 | 3448 | 122 | 50 | 181 | 173 | 88 | 80 | 20 | 0 |
| HV3 | 74498 | 20155 | 57857 | 15874 | 76 | 985 | 0 | 25653 | 30202 | 27205 | 3295 | 847 | 2543 | 3646 | 2213 | 221 | 1468 | 0 |
| HV4 | 36890 | 6204 | 31869 | 10019 | 41 | 504 | 0 | 18465 | 17296 | 17382 | 730 | 250 | 913 | 1012 | 749 | 195 | 343 | 0 |
| HV5 | 5572 | 1883 | 6448 | 1546 | 3 | 105 | 0 | 3063 | 4041 | 3043 | 160 | 51 | 258 | 293 | 219 | 33 | 14 | 0 |
| HV6 | 900 | 401 | 972 | 464 | 0 | 12 | 0 | 696 | 575 | 275 | 7 | 26 | 10 | 43 | 5 | 7 | 11 | 0 |
| HV7 | 1696 | 281 | 1246 | 643 | 3 | 32 | 0 | 1577 | 1229 | 1545 | 109 | 51 | 96 | 112 | 36 | 6 | 9 | 0 |
| HV8 | 0 | 0 | 0 | 0 | 0 | 0 | 0 | 0 | 0 | 0 | 0 | 0 | 0 | 0 | 0 | 0 | 0 | 0 |

Values represent the absolute number of observed sequence counts for each heavy- and light-chain V-gene family combination within the paired dataset.

## Supplementary Table 32: Absolute counts of IGHJ:IG(K/L)J gene family combinations.

|  | KJ1 | KJ2 | KJ3 | KJ4 | KJ5 | LJ1 | LJ2 | LJ3 | LJ4 | LJ5 | LJ6 | LJ7 |
| --- | --- | --- | --- | --- | --- | --- | --- | --- | --- | --- | --- | --- |
| HJ1 | 1995 | 1529 | 548 | 1641 | 480 | 636 | 1359 | 1234 | 0 | 0 | 0 | 18 |
| HJ2 | 2473 | 2198 | 895 | 2872 | 945 | 1318 | 3055 | 1807 | 0 | 0 | 0 | 60 |
| HJ3 | 16935 | 14168 | 5255 | 12010 | 4409 | 7758 | 14292 | 10459 | 0 | 0 | 4 | 248 |
| HJ4 | 56801 | 44514 | 17435 | 45404 | 18095 | 24987 | 47567 | 38352 | 0 | 0 | 4 | 1259 |
| HJ5 | 11402 | 10333 | 3940 | 11810 | 4506 | 6167 | 11716 | 8675 | 0 | 0 | 1 | 306 |
| HJ6 | 16271 | 13748 | 6579 | 14541 | 6220 | 7641 | 17166 | 11698 | 0 | 0 | 1 | 397 |

Values represent the absolute number of observed sequence counts for each heavy- and light-chain J-gene family combination within the paired dataset.

## Supplementary Table 33: Laplace-smoothed pairing correction ratios (R_i,j_) for IGHV:IG(K/L)V gene family combinations.

| IGHV Family | IGLV/IGKV Family | Pair Count | R [CI] | log_2_ R [CI] |
| --- | --- | --- | --- | --- |
| IGHV2 | IGLV9 | 81 | 3.23 [2.57 - 4.02] | 1.69 [1.36 - 2.01] |
| IGHV6 | IGLV5 | 27 | 2.19 [1.45 - 3.19] | 1.13 [0.53 - 1.67] |
| IGHV7 | IGLV5 | 52 | 2.15 [1.61 - 2.82] | 1.1 [0.68 - 1.5] |
| IGHV2 | IGKV5 | 12 | 2.01 [1.04 - 3.51] | 1.01 [0.06 - 1.81] |
| IGHV7 | IGKV5 | 4 | 1.66 [0.45 - 4.24] | 0.73 [-1.15 - 2.08] |
| IGHV7 | IGLV1 | 1578 | 1.64 [1.56 - 1.72] | 0.71 [0.64 - 0.79] |
| IGHV6 | IGKV4 | 465 | 1.6 [1.46 - 1.76] | 0.68 [0.55 - 0.81] |
| IGHV7 | IGLV3 | 1546 | 1.59 [1.51 - 1.67] | 0.67 [0.6 - 0.74] |
| IGHV6 | IGLV9 | 8 | 1.55 [0.67 - 3.05] | 0.63 [-0.58 - 1.61] |
| IGHV3 | IGLV10 | 1469 | 1.5 [1.42 - 1.58] | 0.58 [0.51 - 0.66] |
| IGHV2 | IGLV3 | 3449 | 1.44 [1.39 - 1.49] | 0.53 [0.48 - 0.57] |
| IGHV6 | IGLV1 | 697 | 1.42 [1.32 - 1.53] | 0.51 [0.4 - 0.62] |
| IGHV6 | IGKV2 | 402 | 1.4 [1.26 - 1.54] | 0.48 [0.34 - 0.62] |
| IGHV7 | IGLV4 | 110 | 1.39 [1.14 - 1.68] | 0.48 [0.19 - 0.75] |
| IGHV3 | IGLV4 | 3296 | 1.36 [1.31 - 1.4] | 0.44 [0.39 - 0.49] |
| IGHV7 | IGLV6 | 97 | 1.35 [1.1 - 1.65] | 0.44 [0.13 - 0.72] |
| IGHV2 | IGLV1 | 3162 | 1.33 [1.29 - 1.38] | 0.41 [0.36 - 0.46] |
| IGHV5 | IGLV2 | 4042 | 1.27 [1.24 - 1.31] | 0.35 [0.31 - 0.39] |
| IGHV3 | IGLV8 | 2214 | 1.25 [1.2 - 1.3] | 0.32 [0.26 - 0.38] |
| IGHV5 | IGLV8 | 220 | 1.24 [1.08 - 1.41] | 0.31 [0.11 - 0.5] |
| IGHV1 | IGKV4 | 7665 | 1.2 [1.17 - 1.23] | 0.26 [0.23 - 0.3] |
| IGHV7 | IGLV2 | 1230 | 1.19 [1.13 - 1.26] | 0.26 [0.17 - 0.34] |
| IGHV3 | IGLV7 | 3647 | 1.19 [1.15 - 1.23] | 0.25 [0.2 - 0.3] |
| IGHV4 | IGLV9 | 196 | 1.17 [1.02 - 1.35] | 0.23 [0.02 - 0.43] |
| IGHV5 | IGLV6 | 259 | 1.17 [1.04 - 1.33] | 0.23 [0.05 - 0.41] |
| IGHV4 | IGLV1 | 18466 | 1.17 [1.15 - 1.18] | 0.22 [0.2 - 0.24] |
| IGHV3 | IGKV2 | 20156 | 1.16 [1.14 - 1.18] | 0.22 [0.2 - 0.24] |
| IGHV3 | IGLV6 | 2544 | 1.16 [1.11 - 1.2] | 0.21 [0.15 - 0.27] |
| IGHV5 | IGKV6 | 106 | 1.14 [0.94 - 1.38] | 0.19 [-0.1 - 0.47] |
| IGHV3 | IGLV5 | 848 | 1.14 [1.07 - 1.22] | 0.19 [0.09 - 0.29] |
| IGHV7 | IGLV7 | 113 | 1.13 [0.93 - 1.36] | 0.18 [-0.1 - 0.44] |
| IGHV7 | IGKV4 | 644 | 1.13 [1.04 - 1.22] | 0.18 [0.06 - 0.29] |
| IGHV1 | IGLV7 | 1255 | 1.12 [1.06 - 1.18] | 0.16 [0.08 - 0.24] |
| IGHV1 | IGLV5 | 301 | 1.11 [0.99 - 1.24] | 0.15 [-0.02 - 0.31] |
| IGHV6 | IGLV2 | 576 | 1.1 [1.01 - 1.19] | 0.14 [0.02 - 0.25] |
| IGHV5 | IGKV3 | 6449 | 1.1 [1.07 - 1.13] | 0.14 [0.1 - 0.17] |
| IGHV7 | IGKV6 | 33 | 1.1 [0.75 - 1.54] | 0.13 [-0.41 - 0.62] |
| IGHV4 | IGLV3 | 17383 | 1.09 [1.07 - 1.11] | 0.12 [0.1 - 0.14] |
| IGHV5 | IGLV9 | 34 | 1.09 [0.75 - 1.52] | 0.12 [-0.41 - 0.6] |
| IGHV5 | IGKV2 | 1884 | 1.08 [1.03 - 1.13] | 0.11 [0.05 - 0.18] |
| IGHV1 | IGKV2 | 6822 | 1.08 [1.05 - 1.1] | 0.11 [0.07 - 0.14] |
| IGHV4 | IGKV4 | 10020 | 1.07 [1.05 - 1.09] | 0.1 [0.07 - 0.12] |
| IGHV3 | IGKV6 | 986 | 1.07 [1.0 - 1.13] | 0.09 [0.0 - 0.18] |
| IGHV3 | IGKV1 | 74499 | 1.07 [1.06 - 1.07] | 0.09 [0.08 - 0.1] |
| IGHV1 | IGKV3 | 22601 | 1.06 [1.05 - 1.07] | 0.08 [0.06 - 0.1] |
| IGHV4 | IGKV5 | 42 | 1.06 [0.76 - 1.43] | 0.08 [-0.39 - 0.52] |
| IGHV3 | IGKV5 | 77 | 1.04 [0.82 - 1.3] | 0.05 [-0.29 - 0.38] |
| IGHV5 | IGLV1 | 3064 | 1.03 [1.0 - 1.07] | 0.05 [-0.0 - 0.1] |
| IGHV2 | IGLV6 | 182 | 1.03 [0.88 - 1.19] | 0.04 [-0.18 - 0.25] |
| IGHV4 | IGLV2 | 17297 | 1.02 [1.01 - 1.04] | 0.03 [0.01 - 0.05] |
| IGHV1 | IGLV9 | 116 | 1.02 [0.84 - 1.22] | 0.03 [-0.25 - 0.29] |
| IGHV4 | IGKV6 | 505 | 1.02 [0.93 - 1.11] | 0.03 [-0.1 - 0.15] |
| IGHV5 | IGLV3 | 3044 | 1.02 [0.98 - 1.06] | 0.03 [-0.02 - 0.08] |
| IGHV4 | IGKV3 | 31870 | 1.02 [1.01 - 1.03] | 0.02 [0.01 - 0.04] |
| IGHV6 | IGKV3 | 973 | 1.0 [0.94 - 1.07] | 0.01 [-0.09 - 0.1] |
| IGHV1 | IGLV2 | 11497 | 1.0 [0.98 - 1.02] | -0.0 [-0.03 - 0.02] |
| IGHV2 | IGKV1 | 5579 | 0.99 [0.97 - 1.02] | -0.01 [-0.05 - 0.03] |
| IGHV3 | IGKV3 | 57858 | 0.99 [0.98 - 1.0] | -0.02 [-0.03 - -0.0] |
| IGHV2 | IGLV2 | 2509 | 0.99 [0.95 - 1.03] | -0.02 [-0.08 - 0.04] |
| IGHV4 | IGKV1 | 36891 | 0.99 [0.98 - 1.0] | -0.02 [-0.04 - -0.01] |
| IGHV1 | IGLV3 | 10567 | 0.97 [0.95 - 0.99] | -0.04 [-0.07 - -0.01] |
| IGHV1 | IGLV1 | 10299 | 0.96 [0.94 - 0.97] | -0.07 [-0.09 - -0.04] |
| IGHV3 | IGLV2 | 30203 | 0.96 [0.94 - 0.97] | -0.07 [-0.08 - -0.05] |
| IGHV5 | IGLV7 | 294 | 0.96 [0.85 - 1.07] | -0.07 [-0.24 - 0.1] |
| IGHV2 | IGKV6 | 70 | 0.94 [0.73 - 1.19] | -0.09 [-0.45 - 0.25] |
| IGHV1 | IGKV1 | 23807 | 0.93 [0.92 - 0.95] | -0.1 [-0.12 - -0.08] |
| IGHV3 | IGLV3 | 27206 | 0.91 [0.9 - 0.92] | -0.13 [-0.15 - -0.11] |
| IGHV3 | IGKV4 | 15875 | 0.91 [0.89 - 0.92] | -0.14 [-0.16 - -0.12] |
| IGHV2 | IGKV2 | 1236 | 0.89 [0.84 - 0.94] | -0.18 [-0.26 - -0.1] |
| IGHV5 | IGKV4 | 1547 | 0.88 [0.84 - 0.93] | -0.18 [-0.25 - -0.11] |
| IGHV3 | IGLV1 | 25654 | 0.87 [0.86 - 0.88] | -0.2 [-0.22 - -0.19] |
| IGHV6 | IGLV7 | 44 | 0.86 [0.63 - 1.16] | -0.21 [-0.67 - 0.22] |
| IGHV2 | IGLV5 | 51 | 0.85 [0.64 - 1.12] | -0.23 [-0.65 - 0.17] |
| IGHV1 | IGLV6 | 681 | 0.85 [0.79 - 0.91] | -0.24 [-0.35 - -0.13] |
| IGHV6 | IGKV6 | 13 | 0.85 [0.45 - 1.45] | -0.24 [-1.15 - 0.54] |
| IGHV1 | IGLV4 | 745 | 0.84 [0.78 - 0.9] | -0.25 [-0.36 - -0.15] |
| IGHV6 | IGKV5 | 1 | 0.81 [0.02 - 4.53] | -0.3 [-5.6 - 2.18] |
| IGHV5 | IGKV1 | 5573 | 0.79 [0.77 - 0.82] | -0.33 [-0.37 - -0.29] |
| IGHV4 | IGLV8 | 750 | 0.79 [0.73 - 0.85] | -0.34 [-0.44 - -0.24] |
| IGHV6 | IGKV1 | 901 | 0.78 [0.73 - 0.83] | -0.36 [-0.46 - -0.27] |
| IGHV2 | IGKV3 | 3653 | 0.78 [0.75 - 0.8] | -0.37 [-0.41 - -0.32] |
| IGHV4 | IGLV6 | 914 | 0.78 [0.73 - 0.83] | -0.37 [-0.46 - -0.27] |
| IGHV1 | IGKV6 | 258 | 0.76 [0.67 - 0.86] | -0.39 [-0.57 - -0.21] |
| IGHV2 | IGKV4 | 1064 | 0.76 [0.71 - 0.8] | -0.4 [-0.49 - -0.32] |
| IGHV7 | IGKV1 | 1697 | 0.75 [0.71 - 0.78] | -0.42 [-0.49 - -0.36] |
| IGHV6 | IGLV10 | 12 | 0.74 [0.38 - 1.29] | -0.44 [-1.39 - 0.37] |
| IGHV3 | IGLV9 | 222 | 0.71 [0.62 - 0.81] | -0.49 [-0.69 - -0.3] |
| IGHV1 | IGLV8 | 459 | 0.71 [0.65 - 0.78] | -0.49 [-0.63 - -0.36] |
| IGHV2 | IGLV7 | 174 | 0.7 [0.6 - 0.82] | -0.5 [-0.73 - -0.29] |
| IGHV5 | IGLV5 | 52 | 0.7 [0.52 - 0.92] | -0.52 [-0.94 - -0.13] |
| IGHV7 | IGLV9 | 7 | 0.69 [0.28 - 1.42] | -0.54 [-1.85 - 0.51] |
| IGHV4 | IGKV2 | 6205 | 0.67 [0.65 - 0.68] | -0.58 [-0.62 - -0.55] |
| IGHV1 | IGKV5 | 18 | 0.67 [0.39 - 1.05] | -0.59 [-1.34 - 0.07] |
| IGHV5 | IGLV4 | 161 | 0.66 [0.56 - 0.77] | -0.6 [-0.83 - -0.37] |
| IGHV4 | IGLV10 | 344 | 0.66 [0.59 - 0.73] | -0.61 [-0.77 - -0.46] |
| IGHV7 | IGKV3 | 1247 | 0.65 [0.62 - 0.69] | -0.61 [-0.69 - -0.53] |
| IGHV7 | IGLV8 | 37 | 0.64 [0.45 - 0.88] | -0.64 [-1.15 - -0.18] |
| IGHV4 | IGLV5 | 251 | 0.63 [0.56 - 0.71] | -0.66 [-0.85 - -0.49] |
| IGHV2 | IGLV4 | 123 | 0.63 [0.52 - 0.75] | -0.67 [-0.93 - -0.41] |
| IGHV2 | IGLV8 | 89 | 0.62 [0.5 - 0.77] | -0.68 [-1.0 - -0.38] |
| IGHV4 | IGLV7 | 1013 | 0.62 [0.58 - 0.66] | -0.7 [-0.79 - -0.61] |
| IGHV1 | IGLV10 | 216 | 0.6 [0.53 - 0.69] | -0.73 [-0.93 - -0.53] |
| IGHV4 | IGLV4 | 731 | 0.56 [0.52 - 0.6] | -0.83 [-0.94 - -0.73] |
| IGHV6 | IGLV3 | 276 | 0.56 [0.5 - 0.63] | -0.84 [-1.01 - -0.67] |
| IGHV5 | IGKV5 | 4 | 0.54 [0.15 - 1.38] | -0.89 [-2.77 - 0.46] |
| IGHV7 | IGKV2 | 282 | 0.5 [0.44 - 0.56] | -1.0 [-1.18 - -0.84] |
| IGHV7 | IGLV10 | 10 | 0.31 [0.15 - 0.58] | -1.67 [-2.73 - -0.79] |
| IGHV6 | IGLV6 | 11 | 0.3 [0.15 - 0.54] | -1.73 [-2.73 - -0.89] |
| IGHV2 | IGLV10 | 21 | 0.27 [0.16 - 0.41] | -1.91 [-2.6 - -1.3] |
| IGHV6 | IGLV8 | 6 | 0.2 [0.07 - 0.44] | -2.29 [-3.74 - -1.17] |
| IGHV6 | IGLV4 | 8 | 0.2 [0.09 - 0.39] | -2.33 [-3.54 - -1.35] |
| IGHV5 | IGLV10 | 15 | 0.15 [0.09 - 0.25] | -2.71 [-3.55 - -1.99] |

Values represent the calculated laplace-smoothed pairing correction ratios (R_i,j_) for each heavy- and light-chain V-gene (IGHV:IG(K/L)V) family combination with its 95% Garwood confidence interval, and log_2_ R with the corresponding transformed 95% confidence interval. The table is sorted in descending order from highest to lowest R value.

## Supplementary Table 34: Laplace-smoothed pairing correction ratios (R_i,j_) for IGHJ:IG(K/L)J gene family combinations.

| IGHJ Family | IGLJ/IGKJFamily | Pair Count | R [CI] | log_2_ R [CI] |
| --- | --- | --- | --- | --- |
| IGHJ1 | IGLJ6 | 1 | 3.76 [0.1 - 20.94] | 1.91 [-3.39 - 4.39] |
| IGHJ2 | IGLJ6 | 1 | 2.27 [0.06 - 12.66] | 1.18 [-4.12 - 3.66] |
| IGHJ3 | IGLJ6 | 5 | 2.08 [0.67 - 4.84] | 1.05 [-0.57 - 2.28] |
| IGHJ2 | IGKJ4 | 2873 | 1.18 [1.14 - 1.23] | 0.24 [0.19 - 0.29] |
| IGHJ2 | IGLJ2 | 3056 | 1.17 [1.13 - 1.21] | 0.22 [0.17 - 0.27] |
| IGHJ6 | IGKJ3 | 6580 | 1.14 [1.12 - 1.17] | 0.19 [0.16 - 0.23] |
| IGHJ1 | IGKJ1 | 1996 | 1.13 [1.08 - 1.18] | 0.18 [0.12 - 0.24] |
| IGHJ1 | IGKJ4 | 1642 | 1.12 [1.06 - 1.17] | 0.16 [0.09 - 0.23] |
| IGHJ5 | IGLJ7 | 307 | 1.1 [0.98 - 1.23] | 0.14 [-0.02 - 0.3] |
| IGHJ5 | IGKJ4 | 11811 | 1.1 [1.08 - 1.12] | 0.14 [0.12 - 0.17] |
| IGHJ3 | IGKJ2 | 14169 | 1.09 [1.07 - 1.11] | 0.12 [0.1 - 0.15] |
| IGHJ6 | IGLJ2 | 17167 | 1.09 [1.07 - 1.1] | 0.12 [0.1 - 0.14] |
| IGHJ6 | IGKJ5 | 6221 | 1.08 [1.06 - 1.11] | 0.11 [0.08 - 0.15] |
| IGHJ5 | IGKJ5 | 4507 | 1.07 [1.04 - 1.1] | 0.1 [0.06 - 0.14] |
| IGHJ1 | IGKJ2 | 1530 | 1.06 [1.01 - 1.12] | 0.09 [0.02 - 0.16] |
| IGHJ3 | IGKJ1 | 16936 | 1.06 [1.05 - 1.08] | 0.09 [0.07 - 0.11] |
| IGHJ3 | IGLJ1 | 7759 | 1.06 [1.04 - 1.09] | 0.09 [0.05 - 0.12] |
| IGHJ4 | IGLJ7 | 1260 | 1.06 [1.0 - 1.12] | 0.08 [0.0 - 0.16] |
| IGHJ5 | IGLJ1 | 6168 | 1.05 [1.02 - 1.08] | 0.07 [0.03 - 0.11] |
| IGHJ6 | IGLJ7 | 398 | 1.05 [0.95 - 1.15] | 0.06 [-0.08 - 0.21] |
| IGHJ4 | IGKJ1 | 56802 | 1.04 [1.03 - 1.04] | 0.05 [0.04 - 0.06] |
| IGHJ5 | IGLJ6 | 2 | 1.03 [0.12 - 3.73] | 0.04 [-3.0 - 1.9] |
| IGHJ1 | IGLJ3 | 1235 | 1.03 [0.97 - 1.09] | 0.04 [-0.04 - 0.12] |
| IGHJ4 | IGLJ3 | 38353 | 1.02 [1.01 - 1.04] | 0.04 [0.02 - 0.05] |
| IGHJ5 | IGLJ2 | 11717 | 1.02 [1.0 - 1.03] | 0.02 [-0.0 - 0.05] |
| IGHJ4 | IGKJ5 | 18096 | 1.01 [0.99 - 1.02] | 0.01 [-0.01 - 0.03] |
| IGHJ3 | IGKJ3 | 5256 | 1.01 [0.98 - 1.03] | 0.01 [-0.03 - 0.05] |
| IGHJ3 | IGLJ2 | 14293 | 1.0 [0.98 - 1.01] | -0.0 [-0.03 - 0.02] |
| IGHJ4 | IGLJ1 | 24988 | 0.99 [0.98 - 1.01] | -0.01 [-0.03 - 0.01] |
| IGHJ4 | IGKJ2 | 44515 | 0.99 [0.98 - 1.0] | -0.01 [-0.02 - 0.0] |
| IGHJ6 | IGKJ4 | 14542 | 0.99 [0.98 - 1.01] | -0.01 [-0.03 - 0.01] |
| IGHJ4 | IGKJ4 | 45405 | 0.99 [0.98 - 1.0] | -0.01 [-0.02 - 0.0] |
| IGHJ2 | IGKJ5 | 946 | 0.99 [0.93 - 1.06] | -0.01 [-0.11 - 0.08] |
| IGHJ5 | IGLJ3 | 8676 | 0.99 [0.97 - 1.01] | -0.01 [-0.04 - 0.02] |
| IGHJ2 | IGLJ1 | 1319 | 0.99 [0.94 - 1.04] | -0.02 [-0.1 - 0.06] |
| IGHJ5 | IGKJ2 | 10334 | 0.99 [0.97 - 1.0] | -0.02 [-0.05 - 0.01] |
| IGHJ6 | IGLJ3 | 11699 | 0.98 [0.96 - 0.99] | -0.03 [-0.06 - -0.01] |
| IGHJ4 | IGKJ3 | 17436 | 0.97 [0.96 - 0.99] | -0.04 [-0.06 - -0.02] |
| IGHJ2 | IGLJ7 | 61 | 0.97 [0.74 - 1.24] | -0.05 [-0.44 - 0.31] |
| IGHJ4 | IGLJ2 | 47568 | 0.96 [0.96 - 0.97] | -0.05 [-0.06 - -0.04] |
| IGHJ3 | IGLJ3 | 10460 | 0.96 [0.94 - 0.98] | -0.06 [-0.08 - -0.03] |
| IGHJ6 | IGKJ2 | 13749 | 0.96 [0.94 - 0.97] | -0.06 [-0.09 - -0.04] |
| IGHJ1 | IGKJ3 | 549 | 0.95 [0.87 - 1.04] | -0.07 [-0.19 - 0.05] |
| IGHJ6 | IGLJ1 | 7642 | 0.95 [0.93 - 0.97] | -0.07 [-0.11 - -0.04] |
| IGHJ2 | IGKJ3 | 896 | 0.94 [0.88 - 1.0] | -0.09 [-0.19 - 0.0] |
| IGHJ5 | IGKJ3 | 3941 | 0.94 [0.91 - 0.97] | -0.09 [-0.14 - -0.05] |
| IGHJ6 | IGKJ1 | 16272 | 0.93 [0.91 - 0.94] | -0.11 [-0.13 - -0.09] |
| IGHJ2 | IGKJ2 | 2199 | 0.92 [0.89 - 0.96] | -0.11 [-0.17 - -0.05] |
| IGHJ2 | IGLJ3 | 1808 | 0.91 [0.87 - 0.95] | -0.14 [-0.2 - -0.07] |
| IGHJ3 | IGKJ4 | 12011 | 0.9 [0.89 - 0.92] | -0.15 [-0.17 - -0.12] |
| IGHJ5 | IGKJ1 | 11403 | 0.89 [0.87 - 0.91] | -0.17 [-0.2 - -0.14] |
| IGHJ1 | IGLJ2 | 1360 | 0.86 [0.81 - 0.91] | -0.22 [-0.3 - -0.14] |
| IGHJ2 | IGKJ1 | 2474 | 0.85 [0.82 - 0.88] | -0.24 [-0.29 - -0.18] |
| IGHJ3 | IGKJ5 | 4410 | 0.85 [0.82 - 0.87] | -0.24 [-0.29 - -0.2] |
| IGHJ1 | IGKJ5 | 481 | 0.83 [0.76 - 0.91] | -0.26 [-0.39 - -0.13] |
| IGHJ1 | IGLJ1 | 637 | 0.79 [0.73 - 0.85] | -0.34 [-0.46 - -0.23] |
| IGHJ6 | IGLJ6 | 2 | 0.75 [0.09 - 2.72] | -0.41 [-3.45 - 1.44] |
| IGHJ3 | IGLJ7 | 249 | 0.72 [0.63 - 0.82] | -0.47 [-0.66 - -0.29] |
| IGHJ4 | IGLJ6 | 5 | 0.6 [0.2 - 1.41] | -0.73 [-2.35 - 0.49] |
| IGHJ1 | IGLJ7 | 19 | 0.5 [0.3 - 0.78] | -1.01 [-1.74 - -0.36] |

Values represent the calculated laplace-smoothed pairing correction ratios (R_i,j_) for each heavy- and light-chain J-gene (IGHJ:IG(K/L)J) family combination with its 95% Garwood confidence interval, and log_2_ R with the corresponding transformed 95% confidence interval. The table is sorted in descending order from highest to lowest R value.

# References

1.	Harris LJ, Larson SB, Hasel KW, McPherson A. Refined Structure of an Intact IgG2a Monoclonal Antibody,. *Biochemistry* (1997) 36:1581–1597. doi: 10.1021/bi962514+

2.	Malesys S, Torchet R, Saunier B, Maillet N. AntiBody Sequence Database. *NAR Genom Bioinform* (2024) 6: doi: 10.1093/nargab/lqae171

3.	Olsen TH, Abanades B, Moal IH, Deane CM. KA-Search, a method for rapid and exhaustive sequence identity search of known antibodies. *Scientific Reports 2023 13:1* (2023) 13:11612-. doi: 10.1038/s41598-023-38108-7

4.	Rognes T, Scheffer L, Greiff V, Sandve GK. CompAIRR: ultra-fast comparison of adaptive immune receptor repertoires by exact and approximate sequence matching. *Bioinformatics* (2022) 38:4230–4232. doi: 10.1093/bioinformatics/btac505

5.	Corrie BD, Marthandan N, Zimonja B, Jaglale J, Zhou Y, Barr E, Knoetze N, Breden FMW, Christley S, Scott JK, et al. iReceptor: A platform for querying and analyzing antibody/B-cell and T-cell receptor repertoire data across federated repositories. *Immunol Rev* (2018) 284:24–41. doi: 10.1111/imr.12666

6.	Dudzic P, Chomicz D, Bielska W, Jaszczyszyn I, Zieliński M, Janusz B, Wróbel S, Le Pannérer MM, Philips A, Ponraj P, et al. Conserved heavy/light contacts and germline preferences revealed by a large-scale analysis of natively paired human antibody sequences and structural data. *Communications Biology 2025 8:1* (2025) 8:1110-. doi: 10.1038/s42003-025-08388-y

7.	Dudzic P, Chomicz D, Kończak J, Satława T, Janusz B, Wrobel S, Gawłowski T, Jaszczyszyn I, Bielska W, Demharter S, et al. Large-scale data mining of four billion human antibody variable regions reveals convergence between therapeutic and natural antibodies that constrains search space for biologics drug discovery. *MAbs* (2024) 16: doi: 10.1080/19420862.2024.2361928

8.	Guo Y, Chen K, Kwong PD, Shapiro L, Sheng Z. cAb-Rep: A Database of Curated Antibody Repertoires for Exploring Antibody Diversity and Predicting Antibody Prevalence. *Front Immunol* (2019) 10: doi: 10.3389/fimmu.2019.02365

9.	Kovaltsuk A, Leem J, Kelm S, Snowden J, Deane CM, Krawczyk K. Observed Antibody Space: A Resource for Data Mining Next-Generation Sequencing of Antibody Repertoires. *The Journal of Immunology* (2018) 201:2502–2509. doi: 10.4049/jimmunol.1800708

10.	Olsen TH, Boyles F, Deane CM. Observed Antibody Space: A diverse database of cleaned, annotated, and translated unpaired and paired antibody sequences. *Protein Science* (2022) 31:141–146. doi: 10.1002/pro.4205

11.	Christley S, Scarborough W, Salinas E, Rounds WH, Toby IT, Fonner JM, Levin MK, Kim M, Mock SA, Jordan C, et al. VDJServer: A cloud-based analysis portal and data commons for immune repertoire sequences and rearrangements. *Front Immunol* (2018) 9:348868. doi: 10.3389/fimmu.2018.00976

12.	Młokosiewicz J, Deszyński P, Wilman W, Jaszczyszyn I, Ganesan R, Kovaltsuk A, Leem J, Galson JD, Krawczyk K. AbDiver: a tool to explore the natural antibody landscape to aid therapeutic design. *Bioinformatics* (2022) 38:2628–2630. doi: 10.1093/bioinformatics/btac151

13.	Willis JR, Berndsen ZT, Ma KM, Steichen JM, Schiffner T, Landais E, Liguori A, Kalyuzhniy O, Allen JD, Baboo S, et al. Human immunoglobulin repertoire analysis guides design of vaccine priming immunogens targeting HIV V2-apex broadly neutralizing antibody precursors. *Immunity* (2022) 55:2149. doi: 10.1016/j.immuni.2022.09.001

14.	Schiffner T, Phung I, Ray R, Irimia A, Tian M, Swanson O, Lee JH, Lee CCD, Marina-Zárate E, Cho SY, et al. Vaccination induces broadly neutralizing antibody precursors to HIV gp41. *Nature Immunology 2024 25:6* (2024) 25:1073–1082. doi: 10.1038/s41590-024-01833-w

15.	Ma KM, Sutton HJ, Pratap PP, Steichen JM, Carnathan D, Quinn J, Kalyuzhniy O, Liguori A, Agrawal S, Baboo S, et al. HIV broadly neutralizing antibody precursors to the Apex epitope induced in nonhuman primates. *Sci Immunol* (2025) 10: doi: https://doi.org/10.1126/sciimmunol.adt6660

16.	Steichen JM, Phung I, Salcedo E, Ozorowski G, Willis JR, Baboo S, Liguori A, Cottrell CA, Torres JL, Madden PJ, et al. Vaccine priming of rare HIV broadly neutralizing antibody precursors in nonhuman primates. *Science (1979)* (2024) 384:754–756. doi: 10.1126/science.adj8321

17.	Sun X, Yi C, Zhu Y, Ding L, Xia S, Chen X, Liu M, Gu C, Lu X, Fu Y, et al. Neutralization mechanism of a human antibody with pan-coronavirus reactivity including SARS-CoV-2. *Nature Microbiology 2022 7:7* (2022) 7:1063–1074. doi: 10.1038/s41564-022-01155-3

18.	Yan L, Wang F, Hill M, Brun J, Liang Z, Shi X, Zhang L, He X, Li Y, Huang Q, et al. A broadly neutralizing antibody recognizes a unique epitope with a signature motif common across coronaviruses. *Nature Communications 2025 16:1* (2025) 16:7580-. doi: 10.1038/s41467-025-63101-1

19.	Zhou B, Zhou R, Tang B, Chan JFW, Luo M, Peng Q, Yuan S, Liu H, Mok BWY, Chen B, et al. A broadly neutralizing antibody protects Syrian hamsters against SARS-CoV-2 Omicron challenge. *Nature Communications 2022 13:1* (2022) 13:3589-. doi: 10.1038/s41467-022-31259-7

20.	Mittler E, Serris A, Esterman ES, Florez C, Polanco LC, O’Brien CM, Slough MM, Tynell J, Gröning R, Sun Y, et al. Structural and mechanistic basis of neutralization by a pan-hantavirus protective antibody. *Sci Transl Med* (2023) 15: doi: 10.1126/scitranslmed.adg1855
